# Acid/Base-Steered Cascade Cyclization: An Efficient One-Pot Access to Diverse Isobenzofuranone and Isoindolobenzoxazinone Derivatives

**DOI:** 10.3390/molecules28031443

**Published:** 2023-02-02

**Authors:** Ran Shi, Xiangmin Wang, Xuesi Zhang, Sen Chen, Zu-Li Wang, Huijing Qi, Xin-Ming Xu

**Affiliations:** 1 College of Chemistry and Chemical Engineering, Yantai University, Yantai 264005, China; 2 National Engineering Research Center of Low-Carbon Processing and Utilization of Forest Biomass, Nanjing Forestry University, Nanjing 210037, China; 3 College of Chemistry and Pharmaceutical Sciences, Qingdao Agricultural University, Qingdao 266109, China

**Keywords:** isoindolobenzoxazinones, isobenzofuranones, isatoic anhydrides, cascade cyclization

## Abstract

We herein report the acid/base-steered two distinct reaction pathways of 2-acylbenzoic acids with isatoic anhydrides. In the presence of Na_2_CO_3_, the cascade process consists of the cyclization of 2-acetylbenzoic acid and nucleophilic ring-opening reaction of isatoic anhydride to furnish isobenzofuranone derivatives with high efficiency. However, *p*-toluenesulfonic acid can promote the product isobenzofuranones to undergo sequential intramolecular rearrangment, nucleophilic addition and cyclization reaction to produce diverse isoindolobenzoxazinones in good yields. The synthetic utility of this method was further demonstrated by the gram-scale preparation of the desired products and the facile transformations of the resulting products.

## 1. Introduction

**Figure 1:**
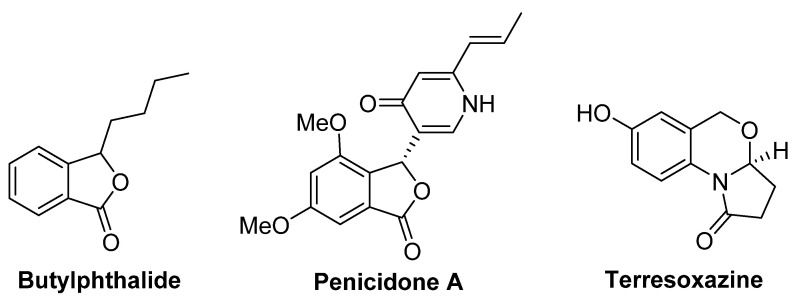
Some representative polyheterocyclic molecules.

Polyheterocyclic scaffolds, as a kind of recognized privileged structure, are widely found in numerous natural products and biologically active compounds. Because of their restricted conformational flexibilities, polyheterocyclic molecules often exhibit the powerful ability to bind to diverse receptors through a variety of non-covalent interactions [1], which allows them to show significant biological activities and play very important roles in chemical biology and drug discovery [2,3,4,5,6]. Among various rigid polyheterocycles, isobenzofuranone and isoindolobenzoxazinone are two prominent substructures because of their broad existence in pharmaceuticals, agrochemicals and biologically active natural products [7], and display a wide range of biological and pharmaceutical properties, which include antibacterial [8,9], antimalarial [10,11,12], anticancer [13,14], anticonvulsant [15,16], and antiulcerogenic activities [17]. For instance, Butylphthalide is a commercial drug for the treatment of acute ischemic stroke (Figure 1), which can increase ischaemic perfusion and reduce the death of nerve cells [18]. Moreover, Penicidone A is the first group of isobenzofuranone analogues isolated from the culture of *Penicillium* sp. and exhibit moderate cytotoxicity against many cancer cell lines [19]. Terresoxazine, a natural product possessing pyrrolobenzoxazine core structure, was isolated from the plant *Tribulus terrestris*, used for a long time in the treatment of a number of diseases in Chinese folk medicine [20]. In addition, these dominant skeletons are also employed as multifunctional ingredients in the total synthesis of natural products and organic materials, and undergo all kinds of chemical transformations [21,22,23,24].

As a result of these practical applications, construction of these polyheterocyclic scaffolds has drawn considerable attention from both academic and industrial organizations. Recently, Mal and co-workers have reviewed the strategies for the synthesis of isobenzofuranones including the lactonization of 2-(hydroxymethyl)-benzoic acids or their analogues that is the most easily conceivable access to phthalides, the reduction of phthalic anhydrides, the oxidation of phthalans and other reactions [25]. On the other hand, the most widely-used and straightforward approaches to isoindolobenzoxazinone derivatives are the Brønsted or Lewis acid-catalyzed cascade cyclization of 2-formylbenzoic acid with anthranilamides [26,27] or anthranilic acid [28,29,30]. Moreover, SanMartin, Zhao et al. also demonstrated that such similar polyheterocyclic scaffold can be constructed through transition metal-catalyzed cascade reactions between alkyne-containing carboxylic acids and anthranilic acids, anthranilamides or functionalized amines [31,32,33,34,35]. Despite tremendous achievements, it is still challenging and highly desirable to develop new, efficient, environmentally benign and cost-effective approaches to diverse polyheterocyclic compounds.

**Scheme 1:**
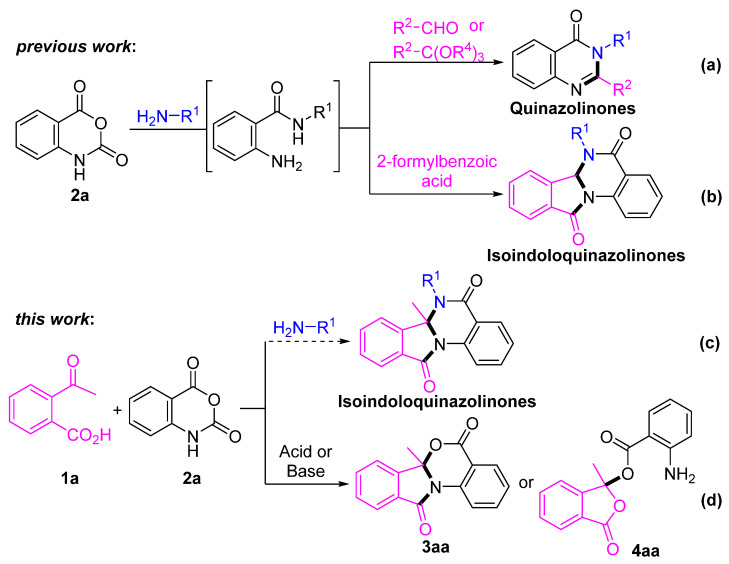
The reaction of isatoic anhydride for accessing to polyheterocyclic skeletons. (**a**) Multi-component reaction of isatoic anhydride, amine and aldehyde/orthoester. (**b**) Multi-component reaction of isatoic anhydride, amine and 2-formylbenzoic acid. (**c**) Our original design of the multi-component reaction. (**d**) This work.

Isatoic anhydride is a cheap and commercially available compound that is a raw material for the synthesis of pharmaceuticals, agrochemicals and dyes, as well as an intermediate in the production of herbicide, Bentazone. The chemistry of isatoic anhydride and its reaction with amines has been investigated in detail [36,37,38,39]. We found that isatoic anhydride undergoes ring opening upon heating with various amines to produce 2-amino-*N*-alkylbenzamides, which can react with aldehydes [40,41,42] or orthoesters [43,44,45,46] and 2-formylbenzoic acid [47,48,49] to produce quinazolinones and isoindoloquinazolinones, respectively (Scheme 1a,b). Continuing with our research efforts to develop novel efficient and eco-friendly accesses to the construction of polyheterocyclic skeletons from easily available starting materials [50,51,52,53,54,55,56], we undertook the current study with the aim to construct polyheterocyclic compounds. We envisioned that the multi-component reaction of 2-acetylbenzoic acid, amines and isatoic anhydride would yield functionalized isoindoloquinazolinones (Scheme 1c). Unfortunately, the experiment did not proceed as assumed and did not yield the desired product, but provided the 3-substituted isobenzofuranones. Reported herein is our systematic study on the cascade reaction of 2-acetylbenzoic acid and isatoic anhydride with the discovery of the base/acid-steered divergent reaction pathways leading selectively to diverse isoindolobenzoxazinone and isobenzofuranone derivatives, respectively (Scheme 1d).

## 2. Results and Discussion

At the outset of this study, we attempted the multi-component reaction of 2-acetylbenzoic acid (**1a**), benzylamine and isatoic anhydride (**2a**), and envisioned that isatoic anhydride (**2a**) undergoes ring opening reaction with benzylamine to produce 2-amino-*N*-benzylbenzamide, which then reacts with 2-acetylbenzoic acid (**1a**) to form isoindoloquinazolinones. Surprisingly, instead of the expected product, 1-methyl-3-oxo-1,3-dihydroisobenzofuran-1-yl 2-aminobenzoate (**4aa**) was isolated as major product in 90% yield (entry 1, Table 1). It’s structure was confirmed unambiguously through the NMR spectra and X-ray single crystal diffraction (see the Supplementary Materials). The result indicates that benzylamine is not involved in the designed reaction and the 3-substituted isobenzofuranone **4aa** is generated most probably from sequential cyclization of 2-acetylbenzoic acid (**1a**) and nucleophilic ring-opening reaction of isatoic anhydride (**2a**) attacked by the product of previous step. We speculate that this is attributed to the lower reactivity of 2-acetylbenzoic acid with 2-amino-*N*-benzylbenzamide than 2-formylbenzoic acid and the incidental cyclization of 2-acetylbenzoic acid under basic conditions [57,58]. Then the experiment was performed in the absence of benzylamine and generated product **4aa** in comparable yield, which confirmed our hypothesis (entry 2, Table 1).

In order to further improve the productivity of **4aa**, we investigated the effect of the amount of Na_2_CO_3_ and a variety of bases on the cascade reaction. The results show that the dosage and type of base have no significant influence and all tested bases could promote the reaction well affording the target product with excellent yield (Table 1, entries 3–6). Interestingly, when this reaction was carried out under acidic conditions, no product **4aa** was obtained (Table 1, entries 7–10), but 6a-methyl-5*H*-benzo[4,5][1,3]oxazino[2,3-*a*]isoindole-5,11(6*aH*)-dione (**3aa**) was produced in 45% yield in the presence of anhydrous *p*-toluenesulfonic acid (entry 7, Table 1). Compound **3aa** might be formed by the cascade reaction of 2a with 2-aminobenzoic acid that might derive from isatoic anhydride hydrolysis or thermal decomposition [59]. Subsequently, the amount of *p*-toluenesulfonic acid was scrutinized and the productivity of this cascade cyclization was increased slightly when 0.8 equivalent of *p*-toluenesulfonic acid was used (entry 12, Table 1). It is noteworthy that the reaction temperature played a vital role in the cascade process. Increasing reaction temperature can improve the efficiency of the reaction, and the chemical yield of **3aa** was drastically improved to 87% when the reaction was executed at 140 °C (entry 17, Table 1). Moreover, the influence of the ratio of reagents was also investigated, and the result show that reducing the amount of isatoic anhydride **2a** would decrease the yield of product, but increasing the amount had no obvious effect (Table 1, entries 19–20). Briefly, the most efficient synthesis of 3-substituted isobenzofuranone **4aa** and isoindolobenzoxazinone **3aa** was to conduct the cascade reaction of 2-acetylbenzoic acid (**1a**) with isatoic anhydride (**2a**) in the presence of Na_2_CO_3_ (20 mol%) or anhydrous *p*-toluenesulfonic acid (80 mol%) in toluene at 110 °C or 140 °C, respectively (Table 1, entry 2 and 17).

**Scheme 2:**
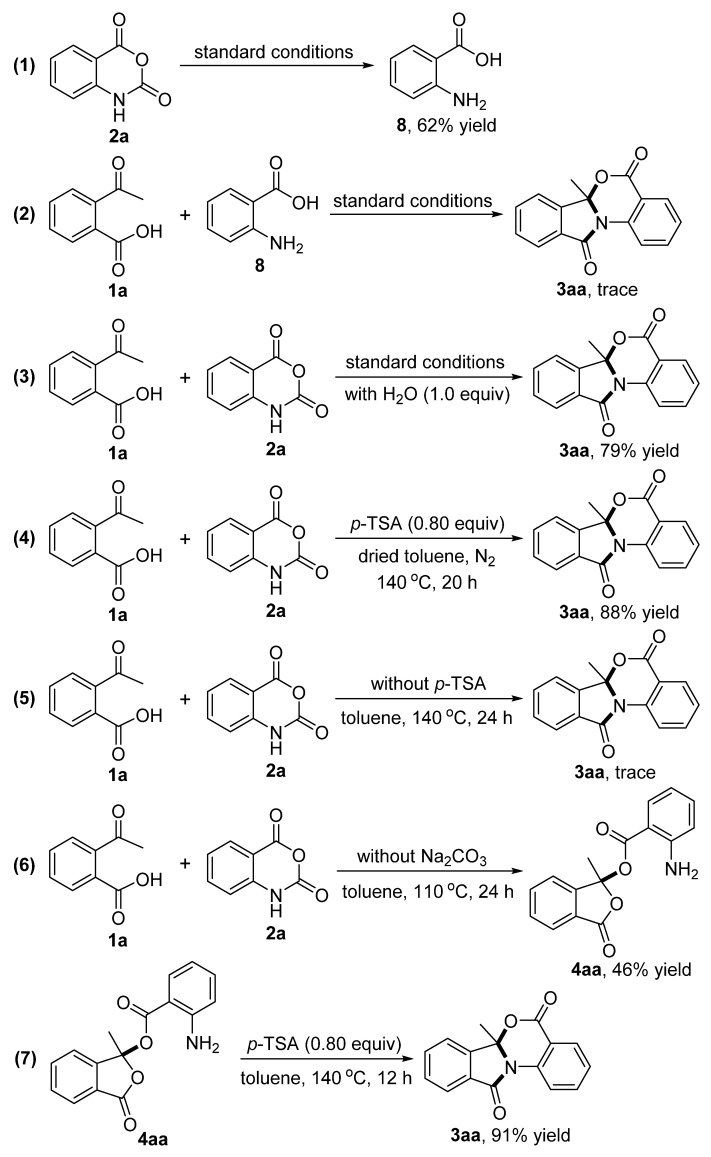
Mechanism studies. (**1**) The decomposition of isatoic anhydride. (**2**) The cyclization of 2-aminobenzoic acid with 2-acetylbenzoic acid. (**3**) The model reaction with one equivalent H_2_O. (**4**) The model reaction was performed in dried toluene. (**5**) The model reaction without *p*-toluenesulfonic acid. (**6**) The model reaction without Na_2_CO_3_. (**7**) The reaction of compound **4aa** in the presence of anhydrous *p*-toluenesulfonic acid.

**Scheme 3:**
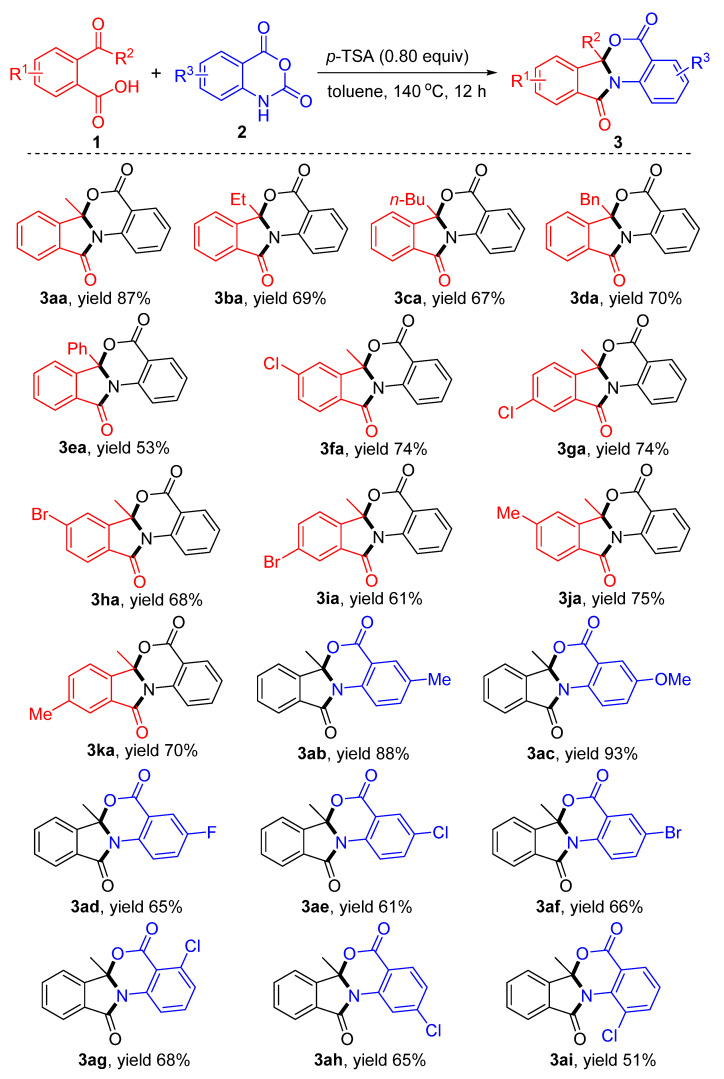
One-pot synthesis of various isoindolobenzoxazinones. Reaction conditions: 2-acylbenzoic acids (1.0 mmol), isatoic anhydrides (1.20 mmol), TsOH (0.80 mmol) and toluene (5.0 mL), stirred at 140 °C in oil bath for 12 h.

**Scheme 4:**
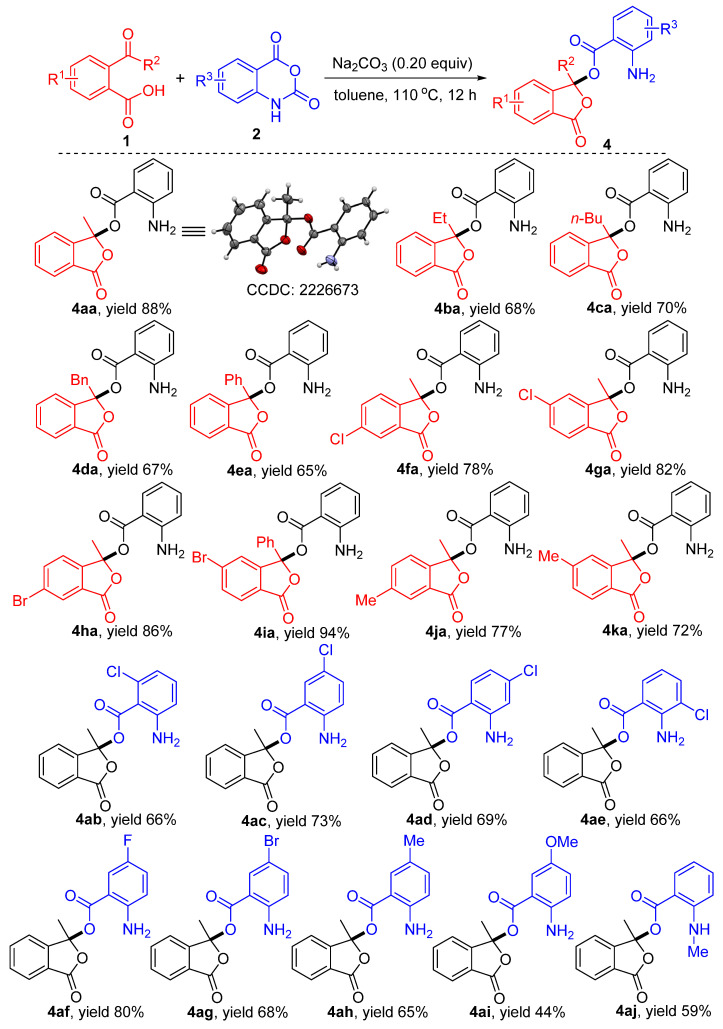
One-pot synthesis of diverse isobenzofuranones. Reaction conditions: 2-acylbenzoic acids (1.0 mmol), isatoic anhydrides (1.20 mmol), Na_2_CO_3_ (0.20 mmol) and toluene (5.0 mL), stirred at 110 °C in oil bath for 12 h.

**Scheme 5:**
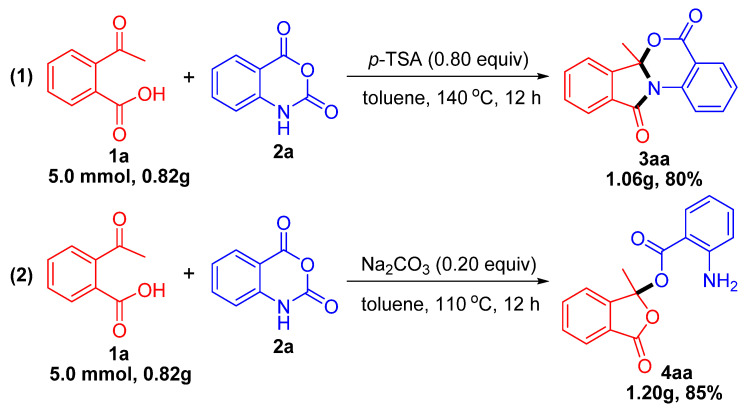
Display the application of this method. (**1**) The gram-scale synthesis of product **3aa**. (**2**) The gram-scale synthesis of product **4aa**.

**Scheme 6:**
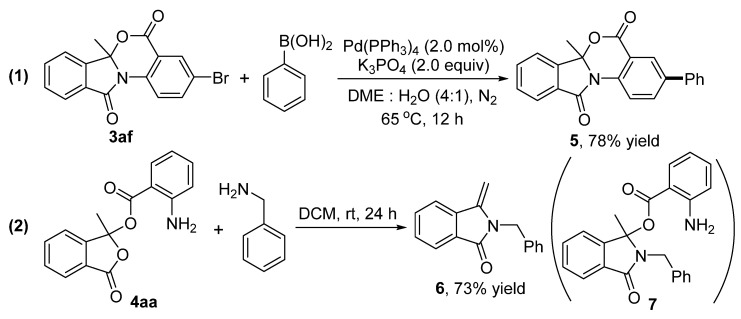
The chemical transformations of resulting products. (**1**) The Suzuki-Miyaura coupling reaction of product **3af** with phenylboronic acid. (**2**) The reaction of product **4aa** with an equivalent benzylamine.

**Scheme 7:**
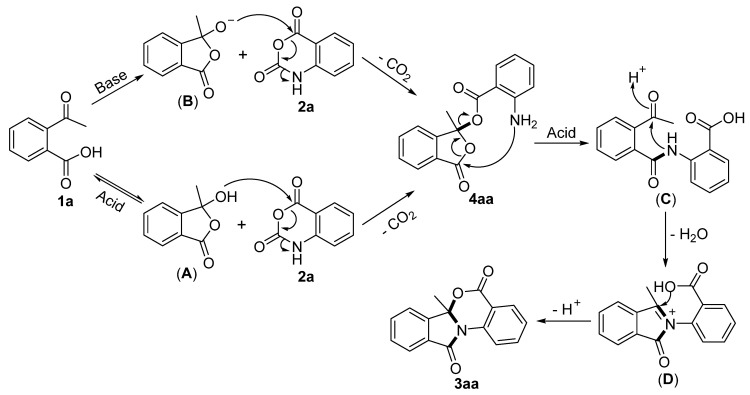
The proposed reaction mechanism.

To further verify the reaction mechanism, some control experiments were carried out. Isatoic anhydride (**2a**) was decomposed into 2-aminobenzoic acid **8** in 62% yield under standard conditions, which could not react with 2-acetylbenzoic acid (**1a**) to deliver the desired product **3aa** (Scheme 2, (1) and (2)). The result indicated that 2-aminobenzoic acid was not the intermediate of this process, which is inconsistent with our hypothesis above. In addition, to check the effect of water present in the solvent, the reaction was performed with one equivalent H_2_O under standard conditions or using dried toluene treated with sodium as the solvent (Scheme 2, (3) and (4)). Additional water would decrease the yield, while the yield of this reaction performed in dried toluene was improved slightly. Unexpectedly, the model reaction can take place without Na_2_CO_3_ to afford the target product **4aa** in 46% yield, but the corresponding product **3aa** was not obtained in the absence of anhydrous *p*-TSA (Scheme 2, (5) and (6)). Furthermore, compound **4aa** could be converted into compound **3aa** in high efficiency when it was heated at 140 °C in toluene with 0.8 equivalent of anhydrous *p*-toluenesulfonic acid (Scheme 2, (7)).

With both optimized conditions in hand, the reaction scope for the synthesis of isoindolobenzoxazinone derivatives was firstly surveyed. A number of substituted 2-acylbenzoic acids and isatoic anhydrides underwent smoothly the cascade reaction under standard conditions to give functionalized isoindolobenzoxazinones (Scheme 3). When the R^2^ group in 2-acylbenzoic acid was evaluated, the desired products **3aa**–**3ea** were obtained in moderate to good yields, but the R^2^ group has significant effect on the cascade process. The results indicate that the larger the bulk of R^2^ group, the lower the yield of product, which may be caused by steric hindrance. Introduction of substituents like halogen or methyl on the benzene ring of 2-acetylbenzoic acid has no obvious effect on the reaction and the corresponding products **3fa**–**3ka** can be produced in 61–75% yield. Moreover, the position and type of substituent on the isatoic anhydride were also inspected and electron-rich substituent exhibited a beneficial effect as the yield of product increased with the increase of electron-donating power of the substituent on phenyl ring. The variation in the chloro substitution pattern had a distinct effect on the outcomes of the reaction. The yields are similar when the chlorine atom is at 5-, 6- and 7-position of isatoic anhydride, but had a drastic decrease using 8-chloro-2*H*-benzo[d][1,3]oxazine-2,4(1*H*)-dione as the substrate, most likely due to the steric hindrance of chlorine atom.

Subsequently, we scrutinized the scope of base-catalyzed cascade reaction of 2-acylbenzoic acids with isatoic anhydrides as depicted in Scheme 4. A variety of substituted 2-acylbenzoic acids reacted efficiently with isatoic anhydride under the catalysis of Na_2_CO_3_ (0.2 equv) to produce diverse isobenzofuranones in high efficiency. For instance, benzoic acids with different acyl group at ortho-position **1a**–**1e** successfully achieved the cascade process to provide the desired products **4aa**–**4ea** in similar yield, which did not show the steric effect as evident as the acid-catalyzed reaction. However, in comparison to the previous reaction, the synthesis of isobenzofuranone derivatives from 2-acylbenzoic acids that contain halogen or methyl on the benzene ring appeared more efficient and the corresponding products **4fa**–**4ka** were synthesized in the yields from 72% to 94%. In addition, various substituted isatoic anhydrides were also transformed into isobenzofuranones in moderate to good yield. On the contrary, electron-rich substituent exhibited a detrimental effect as the yield of product decreased with the increase of electron-donating power of the substituent on phenyl ring. For example, the yield of fluorine-substituted product **4af** was 80%, compared with 44% for methoxy-substituted **4ai**. Moreover, the variation of the position of the chlorine atom on the phenyl ring had a marginal effect on the cascade reaction. Furthermore, *N*-methyl-isatoic anhydride was also found to be suitable substrate, which undergo the cascade reaction with 2-acetylbenzoic acid to give the corresponding product **4aj** in 59% yield.

To display the potential applications of this method, the gram-scale cascade reaction was performed. For example, when 2-acetylbenzoic acid (**1a**, 5.0 mmol) was employed in the gram-scale reaction under two different optimal conditions, the acid-promoted reaction afforded 1.06 g of the isoindolobenzoxazinone derivative **3aa** in 80% yield and the base-catalyzed reaction produced 1.20 g of the isobenzofuranone derivative **4aa** in 85% yield (Scheme 5). This result indicated that the base/acid-steered cascade reaction can be effectively scaled up with slightly lower efficiency.

To further demonstrate the utility of the synthetic protocol, some chemical transformations of products were attempted. In the presence of Pd(PPh_3_)_4_ and tripotassium phosphate, product **3af** underwent the Suzuki-Miyaura coupling reaction with phenylboronic acid in mixed solvent and compound **5** was obtained in 78% yield (Scheme 6, (1)). Moreover, product **4aa** was treated with an equivalent benzylamine in the hope of producing isoindolinone **7**, but which yielded enamide **6** in 73% yield instead. (Scheme 6, (2)). These resulting compounds are useful in organic synthesis, which can be further converted into the variant of bioactive molecules or natural products through many other reactions.

Based on the above experimental results and previous literatures, the plausible mechanism was proposed in Scheme 7. In the presence of base, 2-acetylbenzoic acid (**1a**) turns into the intermediate **B**, followed by the nucleophilic attack on the isatoic anhydride (**2a**) to form product **4aa** and release carbon dioxide. Compound **A** and 2-acetylbenzoic acid (**1a**) are a pair of ring-chain tautomers that exist in an equilibrium in solution, and the former can also react with isatoic anhydride (**2a**) to provide compound **4aa**. That is why the reaction could take place in the absence of Na_2_CO_3_. *p*-Toluenesulfonic acid not only catalyzed the equilibrium reaction, but also promoted the product **4aa** to continue the subsequent reaction. In the presence of acid, compound **4aa** undergo an intramolecular rearrangement to generate the intermediate **C**, followed by the consecutive nucleophilic addition and dehydration reaction to produce the *N*-acyliminium intermediate **D**. Subsequently, the *N*-acyliminium was trapped by carboxyl group within the molecule to afford the final product **3aa** with elimination of a proton.

In summary, we have provided practical methods for selective synthesis of both isoindolobenzoxazinone and isobenzofuranone derivatives from the cascade reaction of 2-acylbenzoic acids with isatoic anhydrides simply using different catalysts. This one-pot synthetic strategy evaded the use of metal catalyst and occurred efficiently with good tolerance of functional groups. The cascade reaction is very promising because that could construct diverse polyheterocyclic skeletons in gram-scale, which are widely found in natural and pharmaceutical compounds. Moreover, the resulting products can undergo a variety of chemical transformations to turn into various synthetic analogues. At present, the biological applications of these compounds and other new reactions of 2-acylbenzoic acids are in progress in our laboratory, and the results will be reported in due course.

## 3. Materials and Methods

### 3.1. General Information

All chemicals were commercially available for direct use unless otherwise stated. Dried toluene was treated with sodium according to standard procedures prior to use. Flash column chromatography was performed on silica gel (100–200). Reactions were monitored using pre-coated, glass-backed silica gel plates and visualized by means of UV irradiation (254 nm) or KmnO_4_. The ^1^H NMR and ^13^C NMR spectra were recorded on a Bruker AV500 spectrometer at ambient temperature. Chemical shifts are reported in ppm with either tetramethylsilane or the residual solvent resonance used as an internal standard. High-resolution mass spectra (HRMS) were measured on a quadrupole time-of-flight mass spectrometer (Q-TOF-MS) using electrospray ionization (ESI) as an ionization method. Crystallographic data were collected on a Rigaku XtaLAB Synergy (Cu) X-ray single crystal diffractometer. All yields reported are isolated yields.

### 3.2. General Procedure for the Synthesis of Isoindolobenzoxazinones ***3***

In a 10 mL reaction tube, substituted 2-acylbenzoic acid **1** (1.0 mmol), substituted isatoic anhydrides **2** (1.20 mmol), TsOH (137.8 mg, 0.80 mmol) and toluene (5.0 mL) were mixed. Then, the reaction tube was capped with a septum and allowed to stir at 140 °C in a pre-heated oil bath for 12 h. After being cooled to room temperature, the solvent was evaporated in vacuo and the residue was purified by column chromatography eluted with a mixture of petroleum ether and ethyl acetate (2:1) to give the pure target product.

6a-methyl-5H-benzo[4,5][1,3]oxazino[2,3-a]isoindole-5,11(6aH)-dione (**3aa**). White solid (231 mg, 87% yield). m.p. 143–145 °C; ^1^H NMR (500 MHz, Chloroform-*d*) δ 8.16 (dd, *J* = 7.8, 1.6 Hz, 1H), 8.12 (dd, *J* = 8.2, 1.1 Hz, 1H), 7.96 (dt, *J* = 7.5, 1.0 Hz, 1H), 7.79–7.72 (m, 3H), 7.67–7.62 (m, 1H), 7.37 (td, *J* = 7.6, 1.1 Hz, 1H), 1.95 (s, 3H); ^13^C NMR (126 MHz, Chloroform-*d*) δ 164.9, 162.1, 144.3, 136.3, 135.9, 134.1, 131.1, 130.8, 130.1, 125.7, 124.9, 122.7, 121.5, 115.7, 92.7, 24.5; HRMS (ESI) *m*/*z*: [M + H]^+^ Calcd. for C_16_H_12_NO_3_ 266.0812; found 266.0817. The characterization data is in accordance with that reported in the literature [60].

6a-ethyl-5H-benzo[4,5][1,3]oxazino[2,3-a]isoindole-5,11(6aH)-dione (**3ba**). White solid (193 mg, 69% yield). m.p. 142–144 °C; ^1^H NMR (500 MHz, Chloroform-*d*) δ 8.18–8.10 (m, 2H), 7.99–7.93 (m, 1H), 7.79–7.69 (m, 3H), 7.68–7.61 (m, 1H), 7.36 (td, *J* = 7.6, 1.2 Hz, 1H), 2.49–2.40 (m, 1H), 2.39–2.29 (m, 1H), 0.58 (td, *J* = 7.4, 1.3 Hz, 3H); ^13^C NMR (126 MHz, Chloroform-*d*) δ 165.4, 162.2, 142.5, 136.1, 135.9, 134.0, 131.2, 131.1, 130.7, 125.7, 124.7, 122.7, 121.3, 116.0, 95.8, 29.6, 8.0; HRMS (ESI) *m*/*z*: [M + H]^+^ Calcd. for C_17_H_14_NO_3_ 280.0968; found 280.0974.

6a-butyl-5H-benzo[4,5][1,3]oxazino[2,3-a]isoindole-5,11(6aH)-dione (**3ca**). White solid (206 mg, 67% yield). m.p. 109–111 °C; ^1^H NMR (500 MHz, Chloroform-*d*) δ 8.15 (dd, *J* = 7.8, 1.5 Hz, 1H), 8.12 (dd, *J* = 8.2, 1.0 Hz, 1H), 7.96 (dt, *J* = 7.7, 1.0 Hz, 1H), 7.80–7.69 (m, 3H), 7.67–7.62 (m, 1H), 7.37 (td, *J* = 7.6, 1.1 Hz, 1H), 2.44–2.36 (m, 1H), 2.30–2.25 (m, 1H), 1.19–1.06 (m, 2H), 1.05–0.94 (m, 1H), 0.82–0.72 (m, 1H), 0.69 (t, *J* = 7.3 Hz, 3H); ^13^C NMR (126 MHz, Chloroform-*d*) δ 165.3, 162.2, 143.0, 136.2, 135.9, 134.0, 131.1, 131.0, 130.7, 125.7, 124.8, 122.7, 121.3, 116.0, 95.3, 36.1, 25.7, 22.1, 13.8; HRMS (ESI) *m*/*z*: [M + Na]^+^ Calcd. for C_19_H_17_NO_3_Na 330.1101; found 330.1109.

6a-benzyl-5H-benzo[4,5][1,3]oxazino[2,3-a]isoindole-5,11(6aH)-dione (**3da**). White solid (239 mg, 70% yield). m.p. 145–147 °C; ^1^H NMR (500 MHz, Chloroform-*d*) δ 8.19–8.12 (m, 2H), 7.80 (td, *J* = 7.9, 1.6 Hz, 1H), 7.75–7.67 (m, 3H), 7.54 (td, *J* = 7.2, 1.6 Hz, 1H), 7.38 (td, *J* = 7.6, 1.1 Hz, 1H), 7.11–7.06 (m, 1H), 7.05–7.00 (m, 2H), 6.69–6.63 (m, 2H), 3.62 (d, *J* = 13.9 Hz, 1H), 3.55 (d, *J* = 13.9 Hz, 1H); ^13^C NMR (126 MHz, Chloroform-*d*) δ 164.9, 162.0, 142.5, 136.6, 136.2, 133.6, 132.3, 131.0, 130.83, 130.77, 130.0, 128.2, 127.6, 125.6, 124.5, 123.2, 121.1, 115.8, 94.6, 42.8; HRMS (ESI) *m*/*z*: [M + H]^+^ Calcd. for C_22_H_16_NO_3_ 342.1125; found 342.1121.

6a-phenyl-5H-benzo[4,5][1,3]oxazino[2,3-a]isoindole-5,11(6aH)-dione (**3ea**). White solid (173 mg, 53% yield). m.p. 189–191 °C; ^1^H NMR (500 MHz, Chloroform-*d*) δ 8.17 (d, *J* = 8.1 Hz, 1H), 8.03–7.96 (m, 2H), 7.69 (td, *J* = 7.8, 1.6 Hz, 1H), 7.63 (td, *J* = 7.4, 1.4 Hz, 1H), 7.59 (td, *J* = 7.4, 1.3 Hz, 1H), 7.55–7.50 (m, 3H), 7.35–7.28 (m, 3H), 7.26–7.22 (m, 1H); ^13^C NMR (126 MHz, Chloroform-*d*) δ 165.9, 162.5, 145.1, 137.5, 136.7, 136.0, 134.2, 130.9, 130.8, 129.7, 129.4, 125.8, 125.7, 124.9, 123.7, 121.3, 116.3, 94.6; HRMS (ESI) *m*/*z*: [M + H]^+^ Calcd. for C_21_H_14_NO_3_ 328.0968; found 328.0964. The characterization data is in accordance with that reported in the literature [25].

8-chloro-6a-methyl-5H-benzo[4,5][1,3]oxazino[2,3-a]isoindole-5,11(6aH)-dione (**3fa**). White solid (221 mg, 74% yield). m.p. 213–215 °C; ^1^H NMR (500 MHz, Chloroform-*d*) δ 8.16 (dd, *J* = 7.8, 1.5 Hz, 1H), 8.09 (dd, *J* = 8.3, 1.2 Hz, 1H), 7.89 (d, *J* = 8.1 Hz, 1H), 7.80–7.73 (m, 2H), 7.62 (dd, *J* = 8.1, 1.7 Hz, 1H), 7.38 (td, *J* = 7.7, 1.1 Hz, 1H), 1.95 (s, 3H); ^13^C NMR (126 MHz, Chloroform-*d*) δ 163.8, 161.6, 145.8, 140.6, 136.05, 136.03, 131.7, 130.9, 128.5, 126.1, 125.9, 123.4, 121.5, 115.6, 92.1, 24.4; HRMS (ESI) *m*/*z*: [M + Na]^+^ Calcd. for C_16_H_10_ClNO_3_Na 322.0241, 324.0212; found 322.0245, 324.0217.

9-chloro-6a-methyl-5H-benzo[4,5][1,3]oxazino[2,3-a]isoindole-5,11(6aH)-dione (3ga). White solid (222 mg, 74% yield). m.p. 162–164 °C; 1H NMR (500 MHz, Chloroform-d) δ 8.16 (dd, J = 7.9, 1.5 Hz, 1H), 8.09 (dd, J = 8.1, 1.0 Hz, 1H), 7.93 (t, J = 1.2 Hz, 1H), 7.77 (td, J = 7.8, 1.6 Hz, 1H), 7.74–7.67 (m, 2H), 7.39 (td, J = 7.6, 1.1 Hz, 1H), 1.94 (s, 3H); 13C NMR (126 MHz, Chloroform-*d*) δ 163.5, 161.8, 142.5, 137.6, 136.1, 136.0, 134.2, 132.0, 130.9, 126.0, 125.0, 124.0, 121.5, 115.6, 92.3, 24.5; HRMS (ESI) *m*/*z*: [M + Na]^+^ Calcd. for C_16_H_10_ClNO_3_Na 322.0241, 324.0212; found 322.0246, 324.0217.

8-bromo-6a-methyl-5H-benzo[4,5][1,3]oxazino[2,3-a]isoindole-5,11(6aH)-dione (3ha). White solid (234 mg, 68% yield). m.p. 219–221 °C; 1H NMR (500 MHz, Chloroform-d) δ 8.16 (dd, J = 7.9, 1.5 Hz, 1H), 8.09 (dd, J = 8.2, 1.1 Hz, 1H), 7.92 (d, J = 1.5 Hz, 1H), 7.82 (d, J = 8.1 Hz, 1H), 7.80–7.74 (m, 2H), 7.38 (td, J = 7.7, 1.1 Hz, 1H), 1.95 (s, 3H); 13C NMR (126 MHz, Chloroform-d) δ 163.9, 161.6, 145.9, 136.1, 136.0, 134.6, 130.9, 129.0, 128.9, 126.3, 126.2, 125.9, 121.5, 115.6, 92.1, 24.4; HRMS (ESI) m/z: [M + Na]+ Calcd. for C16H10BrNO3Na 365.9736, 367.9716; found 365.9730, 367.9709.

9-bromo-6a-methyl-5H-benzo[4,5][1,3]oxazino[2,3-a]isoindole-5,11(6aH)-dione (3ia). White solid (210 mg, 61% yield). m.p. 163–164 °C; 1H NMR (500 MHz, Chloroform-d) δ 8.18–8.12 (m, 1H), 8.12–8.05 (m, 2H), 7.88–7.84 (m, 1H), 7.79–7.72 (m, 1H), 7.64 (dd, J = 8.1, 1.6 Hz, 1H), 7.42–7.34 (m, 1H), 1.93 (s, 3H); 13C NMR (126 MHz, Chloroform-d) δ 163.3, 161.7, 142.9, 137.1, 136.0, 135.9, 132.1, 130.8, 128.0, 126.0, 125.4, 124.2, 121.5, 115.6, 92.4, 24.4; HRMS (ESI) m/z: [M + Na]+ Calcd. for C16H10BrNO3Na 365.9736, 367.9716; found 365.9730, 367.9710.

6a,8-dimethyl-5H-benzo[4,5][1,3]oxazino[2,3-a]isoindole-5,11(6aH)-dione (3ja). White solid (209 mg, 75% yield). m.p. 189–191 °C; 1H NMR (500 MHz, Chloroform-d) δ 8.18–8.07 (m, 2H), 7.83 (d, J = 7.7 Hz, 1H), 7.75 (td, J = 7.7, 6.9, 1.3 Hz, 1H), 7.56 (s, 1H), 7.44 (d, J = 7.7 Hz, 1H), 7.39–7.31 (m, 1H), 2.54 (s, 3H), 1.93 (s, 3H); 13C NMR (126 MHz, Chloroform-d) δ 164.97, 162.22, 145.36, 144.72, 136.45, 135.89, 131.97, 130.73, 127.51, 125.49, 124.69, 123.16, 121.46, 115.67, 92.58, 24.44, 22.27; HRMS (ESI) m/z: [M + H]+ Calcd. for C17H14NO3 280.0968; found 280.0975.

6a,9-dimethyl-5H-benzo[4,5][1,3]oxazino[2,3-a]isoindole-5,11(6aH)-dione (3ka). White solid (195 mg, 70% yield). m.p. 150–152 °C; 1H NMR (500 MHz, Chloroform-d) δ 8.15 (dd, J = 7.8, 1.5 Hz, 1H), 8.11 (d, J = 8.1 Hz, 1H), 7.79–7.72 (m, 2H), 7.64 (d, J = 7.8 Hz, 1H), 7.54 (dd, J = 7.7, 1.5 Hz, 1H), 7.36 (td, J = 7.6, 1.1 Hz, 1H), 2.51 (s, 3H), 1.93 (s, 3H); 13C NMR (126 MHz, Chloroform-d) δ 165.08, 162.32, 141.75, 141.63, 136.40, 135.89, 134.96, 130.75, 130.30, 125.57, 125.03, 122.42, 121.49, 115.73, 92.68, 24.51, 21.67; HRMS (ESI) m/z: [M + H]+ Calcd. for C17H14NO3 280.0968; found 280.0976.

3,6a-dimethyl-5H-benzo[4,5][1,3]oxazino[2,3-a]isoindole-5,11(6aH)-dione (3ab). White solid (246 mg, 88% yield). m.p. 145–147 °C; 1H NMR (500 MHz, Chloroform-d) δ 7.99 (d, J = 8.3 Hz, 1H), 7.97–7.92 (m, 2H), 7.78–7.71 (m, 2H), 7.64 (td, J = 7.2, 1.7 Hz, 1H), 7.57 (dd, J = 8.3, 2.0 Hz, 1H), 2.44 (s, 3H), 1.94 (s, 3H); 13C NMR (126 MHz, Chloroform-d) δ 164.8, 162.4, 144.3, 136.8, 135.7, 134.0, 133.9, 131.0, 130.7, 130.3, 124.8, 122.7, 121.4, 115.5, 92.7, 24.4, 21.1; HRMS (ESI) m/z: [M + H]+ Calcd. for C17H14NO3 280.0968; found 280.0966.

3-methoxy-6a-methyl-5H-benzo[4,5][1,3]oxazino[2,3-a]isoindole-5,11(6aH)-dione (3ac). White solid (275 mg, 93% yield). m.p. 158–160 °C; 1H NMR (500 MHz, Chloroform-d) δ 8.01 (d, J = 8.9 Hz, 1H), 7.97–7.93 (m, 1H), 7.77–7.70 (m, 2H), 7.66–7.59 (m, 2H), 7.32 (dd, J = 8.9, 2.9 Hz, 1H), 3.89 (s, 3H), 1.94 (s, 3H); 13C NMR (126 MHz, Chloroform-d) δ 164.9, 162.3, 157.3, 144.2, 133.9, 131.0, 130.3, 129.8, 124.7, 123.7, 123.1, 122.6, 116.6, 113.0, 92.9, 56.0, 24.2; HRMS (ESI) m/z: [M + Na]+ Calcd. for C17H13NO4Na 318.0737; found 318.0744.

3-fluoro-6a-methyl-5H-benzo[4,5][1,3]oxazino[2,3-a]isoindole-5,11(6aH)-dione (3ad). White solid (184 mg, 65% yield). m.p. 194–196 °C; 1H NMR (500 MHz, Chloroform-d) δ 8.11 (dd, J = 9.0, 4.6 Hz, 1H), 7.96 (dt, J = 7.6, 1.0 Hz, 1H), 7.84 (dd, J = 8.0, 2.9 Hz, 1H), 7.78–7.73 (m, 2H), 7.69–7.63 (m, 1H), 7.51–7.45 (m, 1H), 1.95 (s, 3H); 13C NMR (126 MHz, Chloroform-d) δ 164.9, 161.21, 161.19, 160.7, 158.8, 144.1, 134.2, 132.6, 132.5, 131.2, 129.9, 124.9, 123.64, 123.58, 123.5, 123.3, 122.7, 117.3, 117.2, 117.0, 116.8, 93.0, 24.4; HRMS (ESI) m/z: [M + H]+ Calcd. for C16H11FNO3 284.0717; found 284.0712.

3-chloro-6a-methyl-5H-benzo[4,5][1,3]oxazino[2,3-a]isoindole-5,11(6aH)-dione (3ae). White solid (183 mg, 61% yield). m.p. 168–171 °C; 1H NMR (500 MHz, Chloroform-d) δ 8.14 (d, J = 2.4 Hz, 1H), 8.09 (d, J = 8.7 Hz, 1H), 7.97 (dt, J = 7.6, 0.9 Hz, 1H), 7.78–7.75 (m, 2H), 7.71 (dd, J = 8.7, 2.5 Hz, 1H), 7.69–7.63 (m, 1H), 1.95 (s, 3H); 13C NMR (126 MHz, Chloroform-d) δ 164.8, 161.1, 144.2, 136.0, 134.8, 134.3, 131.33, 131.27, 130.4, 129.9, 125.0, 123.0, 122.7, 116.9, 92.9, 24.5; HRMS (ESI) m/z: [M + Na]+ Calcd. for C16H10ClNO3Na 322.0241, 324.0212; found 322.0248, 324.0218.

3-bromo-6a-methyl-5H-benzo[4,5][1,3]oxazino[2,3-a]isoindole-5,11(6aH)-dione (3af). White solid (227 mg, 66% yield). m.p. 165–167 °C; 1H NMR (500 MHz, Chloroform-d) δ 8.29 (t, J = 2.1 Hz, 1H), 8.03 (dd, J = 8.7, 1.4 Hz, 1H), 7.98–7.94 (m, 1H), 7.86 (dt, J = 8.7, 1.9 Hz, 1H), 7.76 (d, J = 4.2 Hz, 2H), 7.69–7.63 (m, 1H), 1.95 (s, 3H); 13C NMR (126 MHz, Chloroform-d) δ 164.7, 160.9, 144.2, 138.8, 135.2, 134.3, 133.4, 131.3, 129.9, 125.0, 123.1, 122.7, 118.7, 117.2, 92.8, 24.5; HRMS (ESI) m/z: [M + Na]+ Calcd. for C16H10BrNO3Na 365.9736, 367.9716; found 365.9740, 367.9722.

4-chloro-6a-methyl-5H-benzo[4,5][1,3]oxazino[2,3-a]isoindole-5,11(6aH)-dione (3ag). White solid (204 mg, 68% yield). m.p. 212–215 °C; 1H NMR (500 MHz, Chloroform-d) δ 8.06 (dd, J = 8.2, 1.1 Hz, 1H), 7.97 (dt, J = 7.6, 0.9 Hz, 1H), 7.79–7.73 (m, 2H), 7.69–7.60 (m, 2H), 7.42 (dd, J = 8.1, 1.1 Hz, 1H), 1.96 (s, 3H); 13C NMR (126 MHz, Chloroform-d) δ 164.7, 158.8, 144.1, 138.2, 137.5, 135.3, 134.3, 131.2, 129.8, 129.0, 125.0, 122.8, 120.5, 113.9, 92.1, 23.9; HRMS (ESI) m/z: [M + Na]+ Calcd. for C16H10ClNO3Na 322.0241, 324.0212; found 322.0249, 324.0218.

2-chloro-6a-methyl-5H-benzo[4,5][1,3]oxazino[2,3-a]isoindole-5,11(6aH)-dione (3ah). Light yellow solid (195 mg, 65% yield). m.p. 210–213 °C; 1H NMR (500 MHz, Chloroform-d) δ 8.16 (t, J = 1.9 Hz, 1H), 8.09 (dd, J = 8.5, 1.3 Hz, 1H), 7.98–7.96 (m, 1H), 7.78–7.74 (m, 2H), 7.70–7.63 (m, 1H), 7.33 (dt, J = 8.5, 1.6 Hz, 1H), 1.95 (s, 3H); 13C NMR (126 MHz, Chloroform-d) δ 164.7, 161.4, 144.2, 142.4, 137.1, 134.4, 132.0, 131.2, 129.7, 126.2, 125.0, 122.7, 121.5, 113.9, 92.8, 24.6; HRMS (ESI) m/z: [M + Na]+ Calcd. for C16H10ClNO3Na 322.0241, 324.0212; found 322.0248, 324.0219.

1-chloro-6a-methyl-5H-benzo[4,5][1,3]oxazino[2,3-a]isoindole-5,11(6aH)-dione (3ai). White solid (153 mg, 51% yield). m.p. 176–178 °C; ^1^H NMR (500 MHz, Chloroform-*d*) δ 8.08 (dd, *J* = 7.8, 1.4 Hz, 1H), 7.97 (d, *J* = 7.5 Hz, 1H), 7.81 (dd, *J* = 8.1, 1.4 Hz, 1H), 7.78–7.73 (m, 2H), 7.70–7.64 (m, 1H), 7.42 (t, *J* = 7.9 Hz, 1H), 1.97 (s, 3H); ^13^C NMR (126 MHz, Chloroform-*d*) δ 164.7, 161.0, 144.1, 137.0, 134.3, 134.1, 131.3, 130.3, 130.0, 129.1, 128.0, 125.1, 122.8, 120.5, 93.8, 22.9; HRMS (ESI) *m*/*z*: [M + Na]^+^ Calcd. for C_16_H_10_ClNO_3_Na 322.0241, 324.0212; found 322.0246, 324.0217.

### 3.3. General Procedure for the Synthesis of Isobenzofuranones ***4***

A solution of substituted 2-acylbenzoic acid **1** (1.0 mmol), substituted isatoic anhydrides **2** (1.20 mmol) and Na_2_CO_3_ (21.2 mg, 0.20 mmol) in toluene (5.0 mL) was held at reflux in oil bath for 12 h. After being cooled to room temperature, the solvent was evaporated in vacuo and the residue was purified by column chromatography eluted with a mixture of petroleum ether and ethyl acetate (2:1) to give the pure target product.

1-methyl-3-oxo-1,3-dihydroisobenzofuran-1-yl 2-aminobenzoate (**4aa**). White solid (249 mg, 88% yield). m.p. 117–120 °C; ^1^H NMR (500 MHz, Chloroform-*d*) δ 7.93 (dt, *J* = 7.5, 1.0 Hz, 1H), 7.85 (dd, *J* = 8.1, 1.6 Hz, 1H), 7.69 (td, *J* = 7.5, 1.2 Hz, 1H), 7.63–7.57 (m, 2H), 7.30–7.22 (m, 1H), 6.66–6.60 (m, 1H), 6.59 (d, *J* = 8.3 Hz, 1H), 5.53 (br, 2H), 2.08 (s, 3H); ^13^C NMR (126 MHz, Chloroform-*d*) δ 168.0, 165.4, 151.2, 148.4, 134.9, 134.6, 131.5, 130.6, 127.2, 125.6, 121.9, 116.8, 116.3, 109.8, 105.5, 25.7; HRMS (ESI) *m*/*z*: [M + H]^+^ Calcd. for C_16_H_14_NO_4_ 284.0917; found 284.0925.

1-ethyl-3-oxo-1,3-dihydroisobenzofuran-1-yl 2-aminobenzoate (**4ba**). Light yellow solid (202 mg, 68% yield). m.p. 103–105 °C; ^1^H NMR (500 MHz, Chloroform-*d*) δ 7.93 (dt, *J* = 7.6, 0.9 Hz, 1H), 7.88 (dd, *J* = 8.2, 1.6 Hz, 1H), 7.68 (td, *J* = 7.5, 1.1 Hz, 1H), 7.59 (td, *J* = 7.5, 1.0 Hz, 1H), 7.56 (dt, *J* = 7.6, 0.9 Hz, 1H), 7.29–7.24 (m, 1H), 6.68–6.62 (m, 1H), 6.61 (d, *J* = 8.3 Hz, 1H), 4.86 (br, 2H), 2.51 (dq, *J* = 14.8, 7.4 Hz, 1H), 2.25 (dq, *J* = 14.7, 7.4 Hz, 1H), 1.04 (t, *J* = 7.4 Hz, 3H); ^13^C NMR (126 MHz, Chloroform-*d*) δ 168.3, 165.4, 151.0, 147.4, 134.9, 134.5, 131.3, 130.5, 127.9, 125.5, 121.9, 117.0, 116.5, 110.0, 107.4, 31.8, 7.3; HRMS (ESI) *m*/*z*: [M + H]^+^ Calcd. for C_17_H_16_NO_4_ 298.1074; found 298.1080.

1-butyl-3-oxo-1,3-dihydroisobenzofuran-1-yl 2-aminobenzoate (**4ca**). White solid (227 mg, 70% yield). m.p. 112–114 °C; ^1^H NMR (500 MHz, Chloroform-*d*) δ 7.93 (dt, *J* = 7.5, 1.0 Hz, 1H), 7.87 (dd, *J* = 8.1, 1.6 Hz, 1H), 7.68 (td, *J* = 7.5, 1.1 Hz, 1H), 7.63–7.54 (m, 2H), 7.30–7.25 (m, 1H), 6.66 (t, *J* = 7.7 Hz, 1H), 6.62 (d, *J* = 8.3 Hz, 1H), 6.10–4.35 (br, 1H), 2.53–2.45 (m, 1H), 2.23–2.15 (m, 1H), 1.59–1.49 (m, 1H), 1.43–1.26 (m, 3H), 0.91 (t, *J* = 7.2 Hz, 3H); ^13^C NMR (126 MHz, Chloroform-*d*) δ 168.3, 165.4, 150.8, 147.7, 134.9, 134.5, 131.4, 130.5, 127.8, 125.6, 122.0, 117.1, 116.6, 110.2, 107.2, 38.3, 24.9, 22.6, 14.0; HRMS (ESI) *m*/*z*: [M + H]^+^ Calcd. for C_19_H_20_NO_4_ 326.1387; found 326.1380.

1-benzyl-3-oxo-1,3-dihydroisobenzofuran-1-yl 2-aminobenzoate (**4da**). White solid (240 mg, 67% yield). m.p. 139–142 °C; ^1^H NMR (500 MHz, Chloroform-*d*) δ 7.87 (dd, *J* = 8.1, 1.6 Hz, 1H), 7.79 (d, *J* = 7.6 Hz, 1H), 7.63 (td, *J* = 7.5, 1.2 Hz, 1H), 7.52 (td, *J* = 7.5, 0.9 Hz, 1H), 7.43 (d, *J* = 7.6 Hz, 1H), 7.31–7.27 (m, 1H), 7.25–7.17 (m, 5H), 6.68 (ddd, *J* = 8.2, 7.0, 1.1 Hz, 1H), 6.65 (d, *J* = 8.3 Hz, 1H), 3.65 (d, *J* = 2.9 Hz, 2H); ^13^C NMR (126 MHz, Chloroform-*d*) δ 167.7, 165.3, 150.4, 146.9, 135.0, 134.2, 132.5, 131.4, 131.1, 130.5, 128.3, 127.9, 127.6, 125.5, 122.5, 117.3, 117.0, 110.4, 106.2, 44.7; HRMS (ESI) *m*/*z*: [M + H]^+^ Calcd. for C_22_H_18_NO_4_ 360.1230; found 360.1235.

3-oxo-1-phenyl-1,3-dihydroisobenzofuran-1-yl 2-aminobenzoate (**4ea**). White solid (224 mg, 65% yield). m.p. 186–188 °C; ^1^H NMR (500 MHz, Chloroform-*d*) δ 8.12 (d, *J* = 8.2 Hz, 1H), 7.95 (dd, *J* = 7.7, 4.1 Hz, 2H), 7.65 (t, *J* = 7.8 Hz, 1H), 7.61–7.52 (m, 2H), 7.51–7.44 (m, 3H), 7.34–7.15 (m, 4H); ^13^C NMR (126 MHz, Chloroform-*d*) δ 165.8, 162.5, 145.1, 137.5, 136.7, 135.9, 134.2, 130.84, 130.82, 129.7, 129.4, 125.7, 125.6, 124.9, 123.7, 121.3, 116.3, 94.6; HRMS (ESI) *m*/*z*: [M + H]^+^ Calcd. for C_21_H_16_NO_4_ 346.1074; found 346.1084.

5-chloro-1-methyl-3-oxo-1,3-dihydroisobenzofuran-1-yl 2-aminobenzoate (**4fa**). White solid (247 mg, 78% yield). m.p. 102–1035 °C; ^1^H NMR (500 MHz, Chloroform-*d*) δ 7.87 (d, *J* = 1.9 Hz, 1H), 7.84 (dd, *J* = 8.2, 1.6 Hz, 1H), 7.64 (dd, *J* = 8.1, 1.9 Hz, 1H), 7.54 (d, *J* = 8.1 Hz, 1H), 7.29–7.24 (m, 1H), 6.67–6.57 (m, 2H), 5.51 (br, 2H), 2.07 (s, 3H); ^13^C NMR (126 MHz, Chloroform-*d*) δ 166.5, 165.4, 151.2, 146.6, 136.9, 135.1, 134.8, 131.4, 129.1, 125.6, 123.2, 116.9, 116.4, 109.5, 105.2, 25.6; HRMS (ESI) *m*/*z*: [M + Na]^+^ Calcd. for C_16_H_12_ClNO_4_Na 340.0347, 342.0318; found 340.0345, 342.0314.

6-chloro-1-methyl-3-oxo-1,3-dihydroisobenzofuran-1-yl 2-aminobenzoate (**4ga**). White solid (260 mg, 82% yield). m.p. 127–129 °C; ^1^H NMR (500 MHz, Chloroform-*d*) δ 7.88–7.80 (m, 2H), 7.60–7.54 (m, 2H), 7.30–7.26 (m, 1H), 6.68–6.57 (m, 2H), 5.57 (s, 2H), 2.07 (s, 3H); ^13^C NMR (126 MHz, Chloroform-*d*) δ 166.9, 165.4, 151.2, 150.1, 141.3, 135.1, 131.4, 131.2, 126.8, 125.7, 122.5, 117.0, 116.5, 109.5, 104.6, 25.7; HRMS (ESI) *m*/*z*: [M + Na]^+^ Calcd. for C_16_H_12_ClNO_4_Na 340.0347, 342.0318; found 340.0343, 342.0312.

5-bromo-1-methyl-3-oxo-1,3-dihydroisobenzofuran-1-yl 2-aminobenzoate (**4ha**). Light yellow solid (310 mg, 86% yield). m.p. 159–161 °C; ^1^H NMR (500 MHz, Chloroform-*d*) δ 8.17 (dd, *J* = 7.9, 1.5 Hz, 1H), 8.12–8.07 (m, 2H), 7.87 (dd, *J* = 8.1, 1.8 Hz, 1H), 7.77 (td, *J* = 7.7, 1.6 Hz, 1H), 7.64 (d, *J* = 8.1 Hz, 1H), 7.43–7.35 (m, 1H), 1.94 (s, 3H); ^13^C NMR (126 MHz, Chloroform-*d*) δ 163.3, 161.8, 142.9, 137.1, 136.1, 135.9, 132.1, 130.9, 128.0, 126.0, 125.4, 124.2, 121.5, 115.6, 92.4, 24.4; HRMS (ESI) *m*/*z*: [M + Na]^+^ Calcd. for C_16_H_12_BrNO_4_Na 383.9842, 385.9821; found 383.9849, 385.9826.

6-bromo-1-methyl-3-oxo-1,3-dihydroisobenzofuran-1-yl 2-aminobenzoate (**4ia**). Light yellow solid (339 mg, 94% yield). m.p. 138–138 °C; ^1^H NMR (500 MHz, Chloroform-*d*) δ 7.85 (dd, *J* = 8.0, 1.5 Hz, 1H), 7.80–7.75 (m, 2H), 7.73 (dd, *J* = 8.0, 1.6 Hz, 1H), 7.33–7.27 (m, 1H), 6.68 (t, *J* = 8.1 Hz, 2H), 2.07 (s, 3H); ^13^C NMR (126 MHz, Chloroform-*d*) δ 167.0, 165.4, 150.9, 150.1, 135.2, 134.1, 131.4, 129.8, 126.9, 126.2, 125.5, 117.1, 116.7, 109.7, 104.6, 25.7; HRMS (ESI) *m*/*z*: [M + Na]^+^ Calcd. for C_16_H_12_BrNO_4_Na 383.9842, 385.9821; found 383.98497, 385.9826.

1,5-dimethyl-3-oxo-1,3-dihydroisobenzofuran-1-yl 2-aminobenzoate (**4ja**). White solid (229 mg, 77% yield). m.p. 155–157 °C; ^1^H NMR (500 MHz, Chloroform-*d*) δ 7.87 (dd, *J* = 8.1, 1.5 Hz, 1H), 7.80 (d, *J* = 8.1 Hz, 1H), 7.39 (t, *J* = 3.6 Hz, 2H), 7.30–7.26 (m, 1H), 6.70–6.62 (m, 2H), 2.49 (s, 3H), 2.06 (s, 3H); ^13^C NMR (126 MHz, Chloroform-*d*) δ 168.0, 165.4, 150.7, 148.9, 146.0, 134.9, 131.6, 131.5, 125.4, 124.6, 122.4, 117.1, 116.7, 110.3, 105.3, 25.7, 22.3; HRMS (ESI) *m*/*z*: [M + Na]^+^ Calcd. for C_17_H_15_NO_4_Na 320.0893; found 320.0886.

1,6-dimethyl-3-oxo-1,3-dihydroisobenzofuran-1-yl 2-aminobenzoate (**4ka**). White solid (214 mg, 72% yield). m.p. 1537–139 °C; ^1^H NMR (500 MHz, Chloroform-*d*) δ 7.85 (dd, *J* = 8.2, 1.6 Hz, 1H), 7.71 (s, 1H), 7.52–7.46 (m, 2H), 7.30–7.22 (m, 1H), 6.68–6.60 (m, 2H), 5.50 (br, 2H), 2.48 (s, 3H), 2.06 (s, 3H); ^13^C NMR (126 MHz, Chloroform-*d*) δ 168.1, 165.5, 150.8, 145.8, 141.1, 135.6, 134.9, 131.5, 127.4, 125.7, 121.8, 117.0, 116.6, 110.1, 105.6, 25.7, 21.5; HRMS (ESI) *m*/*z*: [M + Na]^+^ Calcd. for C_17_H_15_NO_4_Na 320.0893; found 320.0889.

1-methyl-3-oxo-1,3-dihydroisobenzofuran-1-yl 2-amino-6-chlorobenzoate (**4ab**). White solid (209 mg, 66% yield). m.p. 128–130 °C; ^1^H NMR (500 MHz, Chloroform-*d*) δ 7.93 (dt, *J* = 7.5, 1.0 Hz, 1H), 7.72 (td, *J* = 7.5, 1.1 Hz, 1H), 7.66 (dt, *J* = 7.8, 0.9 Hz, 1H), 7.61 (td, *J* = 7.5, 1.0 Hz, 1H), 7.07 (t, *J* = 8.1 Hz, 1H), 6.72 (dd, *J* = 7.8, 1.0 Hz, 1H), 6.53 (dd, *J* = 8.4, 1.0 Hz, 1H), 4.48 (br, 2H), 2.07 (s, 3H); ^13^C NMR (126 MHz, Chloroform-*d*) δ 167.9, 164.7, 149.9, 147.9, 134.8, 134.2, 132.8, 130.8, 125.6, 122.1, 119.6, 115.2, 113.0, 106.0, 25.6; HRMS (ESI) *m*/*z*: [M + Na]^+^ Calcd. for C_16_H_12_ClNO_4_Na 340.0347, 342.0318; found 340.0353, 342.0324.

1-methyl-3-oxo-1,3-dihydroisobenzofuran-1-yl 2-amino-5-chlorobenzoate (**4ac**). White solid (231 mg, 73% yield). m.p. 138–140 °C; ^1^H NMR (500 MHz, Chloroform-*d*) δ 7.96–7.90 (m, 1H), 7.80 (d, *J* = 2.6 Hz, 1H), 7.71 (td, *J* = 7.3, 1.1 Hz, 1H), 7.61 (t, *J* = 7.2 Hz, 2H), 7.20 (dd, *J* = 8.8, 2.6 Hz, 1H), 6.55 (d, *J* = 8.8 Hz, 1H), 5.58 (br, 2H), 2.09 (s, 3H); ^13^C NMR (126 MHz, Chloroform-*d*) δ 167.9, 164.5, 149.8, 148.1, 134.9, 134.8, 130.7, 130.4, 127.2, 125.6, 121.9, 120.6, 118.3, 110.4, 105.6, 25.7; HRMS (ESI) *m*/*z*: [M + Na]^+^ Calcd. for C_16_H_12_ClNO_4_Na 340.0347, 342.0318; found 340.0353, 342.0325.

1-methyl-3-oxo-1,3-dihydroisobenzofuran-1-yl 2-amino-4-chlorobenzoate (**4ad**). White solid (219 mg, 69% yield). m.p. 125–126 °C; ^1^H NMR (500 MHz, Chloroform-*d*) δ 7.95–7.89 (m, 1H), 7.76 (d, *J* = 8.6 Hz, 1H), 7.70 (td, *J* = 7.5, 1.1 Hz, 1H), 7.63–7.58 (m, 2H), 6.62–6.55 (m, 2H), 5.54 (br, 2H), 2.08 (s, 3H); ^13^C NMR (126 MHz, Chloroform-*d*) δ 167.9, 164.9, 151.9, 148.2, 140.9, 134.7, 132.8, 130.7, 127.1, 125.6, 121.9, 116.8, 116.1, 108.3, 105.5, 25.6; HRMS (ESI) *m*/*z*: [M + Na]^+^ Calcd. for C_16_H_12_ClNO_4_Na 340.0347, 342.0318; found 340.0352, 342.0323.

1-methyl-3-oxo-1,3-dihydroisobenzofuran-1-yl 2-amino-3-chlorobenzoate (**4ae**). White solid (210 mg, 66% yield). m.p. 130–132 °C; ^1^H NMR (500 MHz, Chloroform-*d*) δ 7.99–7.91 (m, 1H), 7.82 (dd, *J* = 8.2, 1.5 Hz, 1H), 7.70 (td, *J* = 7.5, 1.1 Hz, 1H), 7.65–7.57 (m, 2H), 7.40 (dd, *J* = 7.7, 1.5 Hz, 1H), 6.58 (t, *J* = 7.9 Hz, 1H), 6.10 (br, 2H), 2.09 (s, 3H); ^13^C NMR (126 MHz, Chloroform-*d*) δ 167.8, 165.1, 148.1, 147.3, 134.7, 134.6, 130.7, 130.2, 127.1, 125.6, 121.9, 120.4, 115.7, 110.9, 105.6, 25.6; HRMS (ESI) *m*/*z*: [M + Na]^+^ Calcd. for C_16_H_12_ClNO_4_Na 340.0347, 342.0318; found 340.0352, 342.0324.

1-methyl-3-oxo-1,3-dihydroisobenzofuran-1-yl 2-amino-5-fluorobenzoate (**4af**). White solid (241 mg, 80% yield). m.p. 127–129 °C; ^1^H NMR (500 MHz, Chloroform-*d*) δ 7.96–7.90 (m, 1H), 7.71 (td, *J* = 7.3, 1.1 Hz, 1H), 7.61 (t, *J* = 7.3 Hz, 2H), 7.52 (dd, *J* = 9.6, 3.0 Hz, 1H), 7.07–7.01 (m, 1H), 6.56 (dd, *J* = 9.0, 4.5 Hz, 1H), 5.42 (br, 2H), 2.09 (s, 3H); ^13^C NMR (126 MHz, Chloroform-*d*) δ 167.9, 164.6 (d, *J*_C-F_ = 3.8 Hz), 153.8 (d, *J*_C-F_ = 235.6 Hz), 148.0 (d, *J*_C-F_ = 34.0 Hz), 134.7, 130.7, 127.2, 125.6, 123.2, 123.0, 121.9, 118.1 (d, *J*_C-F_ = 7.6 Hz), 116.2 (d, *J*_C-F_ = 23.9 Hz), 109.5, 105.5, 25.6; HRMS (ESI) *m*/*z*: [M + Na]^+^ Calcd. for C_16_H_12_FNO_4_Na 324.0643; found 324.0645.

1-methyl-3-oxo-1,3-dihydroisobenzofuran-1-yl 2-amino-5-bromobenzoate (**4ag**). White solid (245 mg, 68% yield). m.p. 125–128 °C; ^1^H NMR (500 MHz, Chloroform-*d*) δ 7.96–7.90 (m, 2H), 7.71 (td, *J* = 7.4, 1.1 Hz, 1H), 7.64–7.58 (m, 2H), 7.32 (dd, *J* = 8.8, 2.4 Hz, 1H), 6.50 (d, *J* = 8.8 Hz, 1H), 4.89 (br, 2H), 2.09 (s, 3H); ^13^C NMR (126 MHz, Chloroform-*d*) δ 167.9, 164.4, 150.1, 148.1, 137.6, 134.8, 133.4, 130.7, 127.2, 125.6, 121.9, 118.6, 111.1, 107.3, 105.6, 25.7; HRMS (ESI) *m*/*z*: [M + Na]^+^ Calcd. for C_16_H_12_BrNO_4_Na 383.9842, 385.9821; found 383.9836, 385.9816.

1-methyl-3-oxo-1,3-dihydroisobenzofuran-1-yl 2-amino-5-methylbenzoate (**4ah**). Light yellow oil (193 mg, 65% yield). ^1^H NMR (500 MHz, Chloroform-*d*) δ 7.91 (dd, *J* = 7.6, 1.1 Hz, 1H), 7.68 (td, *J* = 7.5, 1.1 Hz, 1H), 7.65–7.56 (m, 3H), 7.09 (dd, *J* = 8.4, 2.2 Hz, 1H), 6.55 (d, *J* = 8.4 Hz, 1H), 5.19 (br, 2H), 2.23 (s, 3H), 2.09 (s, 3H); ^13^C NMR (126 MHz, Chloroform-*d*) δ 168.0, 165.4, 148.7, 148.4, 136.1, 134.6, 130.9, 130.5, 127.1, 125.7, 125.5, 122.0, 117.2, 109.8, 105.5, 25.6, 20.4; HRMS (ESI) *m*/*z*: [M + H]^+^ Calcd. for C_17_H_16_NO_4_ 298.1074; found 298.1082.

1-methyl-3-oxo-1,3-dihydroisobenzofuran-1-yl 2-amino-5-methoxybenzoate (**4ai**). Light yellow solid (138 mg, 44% yield). m.p. 159–162 °C; ^1^H NMR (500 MHz, Chloroform-*d*) δ 8.01 (d, *J* = 8.9 Hz, 1H), 7.95 (d, *J* = 7.4 Hz, 1H), 7.78–7.71 (m, 2H), 7.67–7.60 (m, 2H), 7.33 (dd, *J* = 8.9, 2.9 Hz, 1H), 3.89 (s, 3H), 1.94 (s, 3H); ^13^C NMR (126 MHz, Chloroform-*d*) δ 164.9, 162.3, 157.3, 144.2, 133.9, 131.0, 130.3, 129.8, 124.7, 123.7, 123.1, 122.6, 116.6, 113.0, 92.9, 56.0, 24.2; HRMS (ESI) *m*/*z*: [M + Na]^+^ Calcd. for C_17_H_15_NO_5_Na 336.0842; found 336.0846.

1-methyl-3-oxo-1,3-dihydroisobenzofuran-1-yl 2-(methylamino)benzoate (**4aj**). White solid (175 mg, 59% yield). m.p. 135–137 °C; ^1^H NMR (500 MHz, Chloroform-*d*) δ 7.96–7.88 (m, 2H), 7.69 (td, *J* = 7.4, 1.1 Hz, 1H), 7.64–7.56 (m, 2H), 7.41–7.35 (m, 1H), 6.63 (d, *J* = 8.6 Hz, 1H), 6.60 (t, *J* = 7.6 Hz, 1H), 2.80 (s, 3H), 2.07 (s, 3H); ^13^C NMR (126 MHz, Chloroform-*d*) δ 168.0, 166.0, 152.7, 148.5, 135.5, 134.6, 131.9, 130.5, 127.2, 125.5, 121.8, 114.4, 111.0, 108.8, 105.4, 29.5, 25.8; HRMS (ESI) *m*/*z*: [M + Na]^+^ Calcd. for C_17_H_15_NO_4_Na 320.0893; found 320.0898.

### 3.4. The Procedure for the Suzuki-Miyaura Coupling Reaction

In a flame-dried 10 mL reaction tube, Pd(PPh_3_)_4_ (2.0 mol %, 7.0 mg), K_3_PO_4_ (0.60 mmol, 127 mg, 2.0 equiv) and DME (5 mL) were mixed. The solution was degassed with argon for 10 min. After that, product 3af (0.30 mmol, 103 mg, 1.0 equiv), Phenyl boronic acid (0.36 mmol, 44 mg, 1.2 equiv) and water (1.25 mL) were added. Then, the reaction tube was capped with a septum and allowed to stir at 65 °C in a pre-heated oil bath for 12 h. The reaction was monitored by TLC and upon completion of the reaction, the reaction mixture was cooled to room temperature and diluted with water (10 mL) and extracted with ethyl acetate (3 × 30 mL). Further, the combined organic layer was washed with brine (50 mL) and dried over anhydrous Na_2_SO_4_. The organic layer was concentrated under reduced pressure and the residue was purified by flash chromatography on silica gel to give pure desired product 5.

6a-methyl-3-phenyl-5H-benzo[4,5][1,3]oxazino[2,3-a]isoindole-5,11(6aH)-dione (**5**). White solid (80 mg, 78% yield). m.p. 166–168 °C; ^1^H NMR (500 MHz, Chloroform-*d*) δ 8.40 (d, *J* = 2.2 Hz, 1H), 8.20 (d, *J* = 8.5 Hz, 1H), 8.03–7.96 (m, 2H), 7.82–7.73 (m, 2H), 7.70–7.62 (m, 3H), 7.49 (t, *J* = 7.7 Hz, 2H), 7.41 (t, *J* = 7.3 Hz, 1H), 2.00 (s, 3H); ^13^C NMR (126 MHz, Chloroform-*d*) δ 164.9, 162.2, 144.4, 139.2, 138.9, 135.3, 134.5, 134.1, 131.1, 130.2, 129.2, 129.0, 128.2, 127.1, 124.9, 122.7, 121.9, 116.0, 92.8, 24.6; HRMS (ESI) *m*/*z* [M + Na]^+^ Calcd. for C_22_H_15_NO_3_Na 364.0944; found 364.0937.

### 3.5. The Procedure for the Reaction of ***4aa*** with Benzylamine

To a solution of 4aa (85 mg, 0.30 mmol, 1.0 equiv) in 3 mL DCM, benzylamine (36 mg, 0.33 mmol, 1.1 equiv) was added. Then, the contents were stirred at room temperature for 24 h. Upon completion of the reaction, it was quenched with 5 mL water and extracted with ethyl acetate (3 × 10 mL). The combined organic layer was dried over Na_2_SO_4_ and the organic layer was collected and concentrated under reduced pressure. The residue was purified by a silica gel column chromatography using ethyl acetate/hexane (1:3) as the eluent to obtain 6.

2-benzyl-3-methyleneisoindolin-1-one (**6**). White solid (52 mg, 73% yield). ^1^H NMR (500 MHz, Chloroform-*d*) δ 7.89 (d, *J* = 7.2 Hz, 1H), 7.67 (d, *J* = 7.9 Hz, 1H), 7.59 (td, *J* = 7.5, 1.2 Hz, 1H), 7.52 (td, *J* = 7.5, 1.1 Hz, 1H), 7.34–7.21 (m, 5H), 5.15 (d, *J* = 2.3 Hz, 1H), 5.01 (s, 2H), 4.79 (d, *J* = 2.3 Hz, 1H); ^13^C NMR (126 MHz, Chloroform-*d*) δ 167.4, 141.7, 137.0, 136.5, 132.2, 129.7, 129.3, 128.8, 127.5, 127.2, 123.5, 120.0, 90.2, 43.3. The NMR data is the same as previously reported in the literature [61,62].

## Figures and Tables

**Table 1:** Optimization of the cascade cyclization reaction of **1a** and **2a** ^1^.

Entry	Cat. (equiv)	Temp. (°C)	Solvent (0.2 M)	3aa ^2^ (%)	4aa ^2^ (%)
1 ^3^	Na_2_CO_3_ (0.2)	110	toluene	--	90
2	Na_2_CO_3_ (0.2)	110	toluene	--	88
3	Na_2_CO_3_ (1.0)	110	toluene	--	87
4	K_2_CO_3_ (1.0)	110	toluene	--	85
5	Cs_2_CO_3_ (1.0)	110	toluene	--	82
6	NaHCO_3_ (1.0)	110	toluene	trace	79
7	*p*-TSA (1.0)	110	toluene	45	n.d. ^4^
8	AcOH (1.0)	110	toluene	trace	n.d. ^4^
9	HCl (1.0)	110	toluene	trace	n.d. ^4^
10	CF_3_SO_3_H (1.0)	110	toluene	trace	n.d. ^4^
11	*p*-TSA (2.0)	110	toluene	36	n.d. ^4^
12	*p*-TSA (0.8)	110	toluene	56	n.d. ^4^
13	*p*-TSA (0.6)	110	toluene	28	n.d. ^4^
14	*p*-TSA (0.4)	110	toluene	23	n.d. ^4^
15	*p*-TSA (0.8)	120	toluene	70	n.d. ^4^
16	*p*-TSA (0.8)	130	toluene	83	n.d. ^4^
17	*p*-TSA (0.8)	140	toluene	87	n.d. ^4^
18	*p*-TSA (0.8)	150	toluene	84	n.d. ^4^
19 ^5^	*p*-TSA (0.8)	140	toluene	81	n.d. ^4^
20 ^6^	*p*-TSA (0.8)	140	toluene	86	n.d. ^4^

^1^ Reaction conditions: **1a** (1.0 mmol), **2a** (1.2 mmol), catalysts and temperature (see the appropriate column), toluene (5.0 mL), 12 h. ^2^ Yield of isolated product. ^3^ With 1.2 equivalent of benzylamine. ^4^ Not determined. ^5^ With 1.0 equivalent of **2a**. ^6^ With 1.5 equivalent of **2a**.

## Data Availability

Not applicable.

## Supplementary Materials

The following supporting information can be downloaded at: https://www.mdpi.com/article/10.3390/molecules28031443/s1. The crystallographic data of **4aa** and NMR (^1^H, ^13^C) spectra of all new compounds. Figure S1. X-ray molecular structure of **4aa**. The molecular structure is depicted in an ellipsoid style at 50% probability level.

## Sample Availability

Samples of all obtained compounds are available from the authors.
