# Supplementary material for: Acid/Base-Steered Cascade Cyclization: An Efficient One-Pot Access to Diverse Isobenzofuranone and Isoindolobenzoxazinone Derivatives
Source: Molecules. 2023 Feb 2;28(3):1443. doi: 10.3390/molecules28031443 (PMC9921644; doi:10.3390/molecules28031443)
Supplement: Supplementary file 1 [file molecules-28-01443-s001.zip › molecules-2170446-supplementary.pdf]

Acid/Base-Steered Cascade Cyclization: An Efficient One-Pot Access to Diverse Isobenzofuranone and Isoindolobenzoxazinone Derivatives

Supplementary Materials

Ran Shi[1], Xiangmin Wang[1,*], Xuesi Zhang[1], Sen Chen[1], Zu-Li Wang[2,3,*], Huijing Qi[1], Xin-Ming Xu[1,*]

[1] College of Chemistry and Chemical Engineering, Yantai University, Yantai 264005, China

[2] National Engineering Research Center of Low-Carbon Processing and Utilization of Forest Biomass, Nanjing Forestry University, Nanjing 210037, China

[3] College of Chemistry and Pharmaceutical Sciences, Qingdao Agricultural University, Qingdao 266109, China

* Correspondence: ydwangxm@ytu.edu.cn (X. W.); 201201055@qau.edu.cn (Z.-L. W.); xuxm18@ytu.edu.cn (X.-M. X.)

1. General Information S2

2. Crystallographic Data of 4aa S2

3. Mechanism Studies S3

4. Copies of ^{1}H and ^{13}C NMR Spectra S7

1. General information

All chemicals were commercially available for direct use unless otherwise stated. Dried was treated with sodium according to standard procedures prior to use. Flash column chromatography was performed on silica gel (100-200). Reactions were monitored using pre-coated, glass-backed silica gel plates and visualized by means of UV irradiation (254 nm) or $KMnO_4$, phosphomolybdic acid, ninhydrin, and pancaldi solution. ^1H NMR and ^{13}C NMR spectra were recorded using 500 MHz spectrometers at ambient temperature. Chemical shifts are reported in ppm with either tetramethylsilane or the residual solvent resonance used as an internal standard. Abbreviations are used in the description of NMR data as follows: chemical shift (δ, ppm), multiplicity (s = singlet, d = doublet, t = triplet, m = multiplet), coupling constant (*J*, Hz). High-resolution mass spectra (HRMS) were measured on a quadrupole time-of-flight mass spectrometer (Q-TOF-MS) using electrospray ionization (ESI) as an ionization method. Crystallographic data were collected on a Rigaku XtaLAB Synergy (Cu) X-ray single crystal diffractometer. All yields reported were isolated yields.

2. Crystallographic Data of 4aa

High quality single crystals of **4aa** were cultivated from the evaporation of a solution of **4aa** in the mixture of EtOAc and *n*-hexane. As depicted below, the molecular structures was determined by X-ray diffraction analysis.

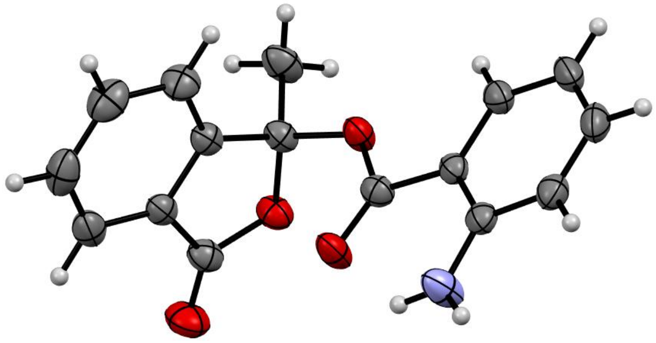

Figure S1. X-ray molecular structure of **4aa**. The molecular structure is depicted in an ellipsoid style at 50% probability level.

2.1 Crystallographic data and structure refinement of 4aa

CCDC Number	2226673
Empirical formula	$C_{16}H_{13}NO_4$
Formula weight	283.27
Temperature	173.0 K
Wavelength	0.71073 Å
Crystal system	Tetragonal
Space group	P 41 21 2
a	9.6989 (6) Å
b	9.6989 (6) Å
c	29.7180 (2) Å
α	90 °
β	90 °
γ	90 °
Volumn	2795.5 (4) $Å^3$
Z	8
Density (calculated)	1.346
Absorption coefficient	0.098 mm^{-1}
F(000)	1184.0
Radiation type	MoK\a
Completeness to theta =30.761°	1.46/0.87
R (reflections)	0.0467 (2569)
wR2 (reflections)	0.0936 (3363)

3. Mechanism Studies

3.1 The Reaction of Isatoic Anhydride under Standard Conditions

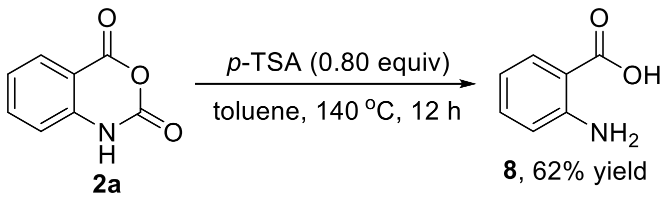

In a 5 mL reaction tube, isatoic anhydride **2a** (0.50 mmol, 82 mg), TsOH (0.4 mmol, 68.9 mg) and toluene (2.5 mL) were mixed. Then, the reaction tube was capped with a septum and allowed to stir at 140 °C in a pre-heated oil bath for 12 h. After being cooled to room temperature, the solvent was evaporated in vacuo and the residue was purified by column chromatography eluted with a mixture of petroleum ether and ethyl acetate (1:1) to give the desired 2-aminobenzoic acid in 62% yield.

3.2 The Reaction of 2-Acetylbenzoic Acid and 2-Aminobenzoic Acid

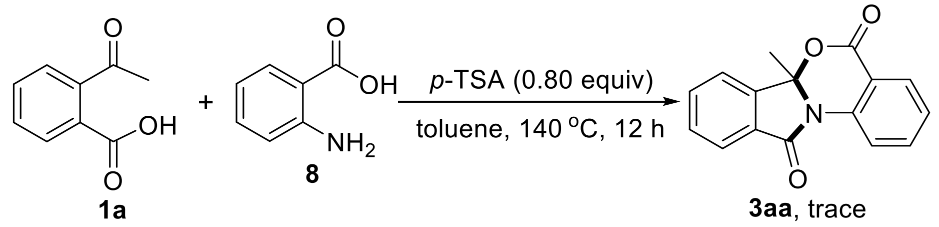

In a 5 mL reaction tube, 2-acetylbenzoic acid **1a** (0.5 mmol, 82.1 mg), 2-aminobenzoic acid **8** (0.5 mmol, 68.6 mg), TsOH (0.4 mmol, 68.9 mg) and toluene (2.5 mL) were mixed. Then, the reaction tube was capped with a septum and allowed to stir at 140 °C in a pre-heated oil bath for 12 h. The target product **3aa** was not obtained and plenty of starting material remained.

3.3 The Reaction with an Additional Equivlent H_2O

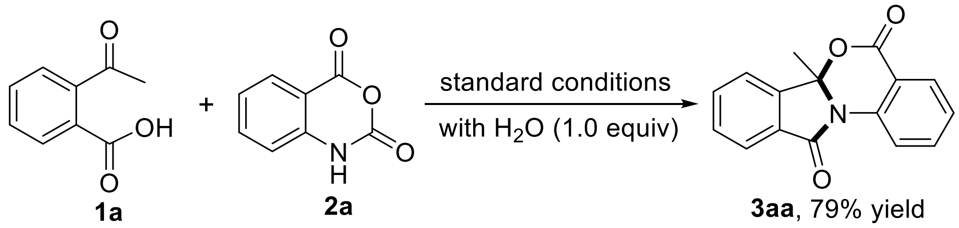

In a 5 mL reaction tube, 2-acetylbenzoic acid **1a** (0.5 mmol, 82.1 mg), isatoic anhydride **2a** (0.6 mmol, 97.8 mg), TsOH (0.4 mmol, 68.9 mg), H_2O (0.5 mmol, 9.0 mg) and toluene (2.5 mL) were mixed. Then, the reaction tube was capped with a septum and allowed to stir at 140 °C in a pre-heated oil bath for 12 h. After being cooled to room temperature, the solvent was evaporated in vacuo and the residue was purified by column chromatography eluted with a mixture of petroleum ether and ethyl acetate (2:1) to give the target product in 79% yield.

3.4 The Reaction in Dried Toluene

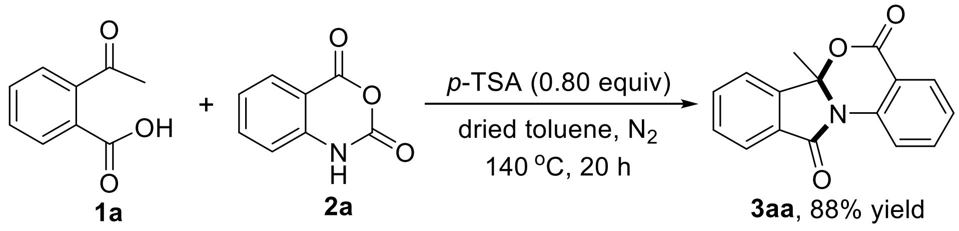

In a 5 mL reaction tube, 2-acetylbenzoic acid **1a** (0.5 mmol, 82.1 mg), isatoic anhydride **2a** (0.6 mmol, 97.8 mg), TsOH (0.4 mmol, 68.9 mg), and dried toluene (2.5 mL) were mixed. Then, the reaction tube was capped with a septum and allowed to stir at 140 °C in a pre-heated oil bath for 12 h. After being cooled to room temperature, the solvent was evaporated in vacuo and the residue was purified by column chromatography eluted with a mixture of petroleum ether and ethyl acetate (2:1) to affored the target product in 88% yield.

3.5 The Reaction without *p*-TSA

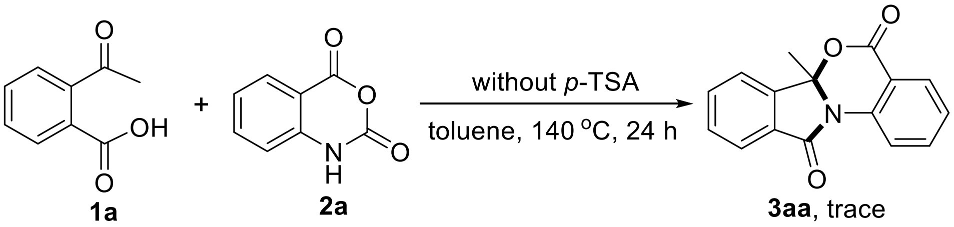

In a 5 mL reaction tube, 2-acetylbenzoic acid **1a** (0.5 mmol, 82.1 mg), isatoic anhydride **2a** (0.6 mmol, 97.8 mg) and toluene (2.5 mL) were mixed. Then, the reaction tube was capped with a septum and allowed to stir at 140 °C in a pre-heated oil bath for 24 h. The target product **3aa** was not obtained and plenty of starting material remained.

3.6 The Reaction without Na_2CO_3

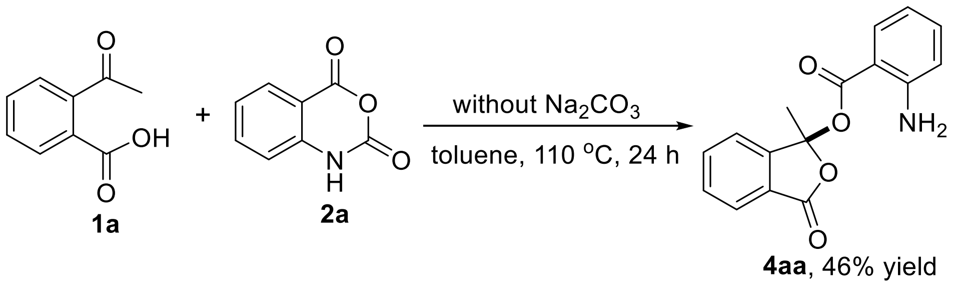

A solution of 2-acetylbenzoic acid **1a** (0.5 mmol, 82.1 mg) and isatoic anhydride **2a** (0.6 mmol, 97.8 mg) in toluene (2.5 mL) was held at reflux in oil bath for 12 h. After being cooled to room temperature, the solvent was evaporated in vacuo and the residue was purified by column chromatography eluted with a mixture of petroleum ether and ethyl acetate (2:1) to give the pure target product in 46% yield. .

3.7 The Transformation of 4aa at High Temperature with TsOH

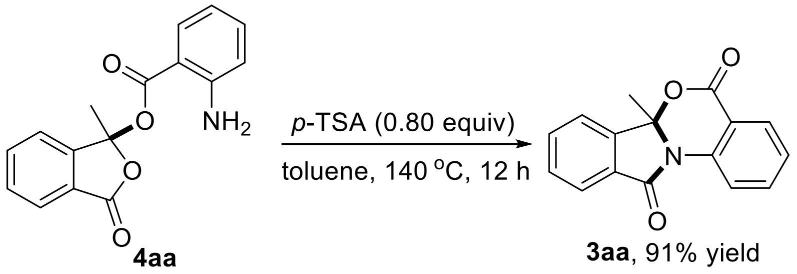

In a 5 mL reaction tube, compound **4aa** (0.5 mmol, 141.6 mg), TsOH (0.4 mmol, 68.9 mg) and toluene (2.5 mL) were mixed. Then, the reaction tube was capped with a septum and allowed to stir at 140 °C in a pre-heated oil bath for 12 h. After being cooled to room temperature, the solvent was evaporated in vacuo and the residue was purified by column chromatography eluted with a mixture of petroleum ether and ethyl acetate (2:1) to give the target product in 91% yield.

4. Copies of ^1H NMR and ^{13}C NMR Spectra of Products

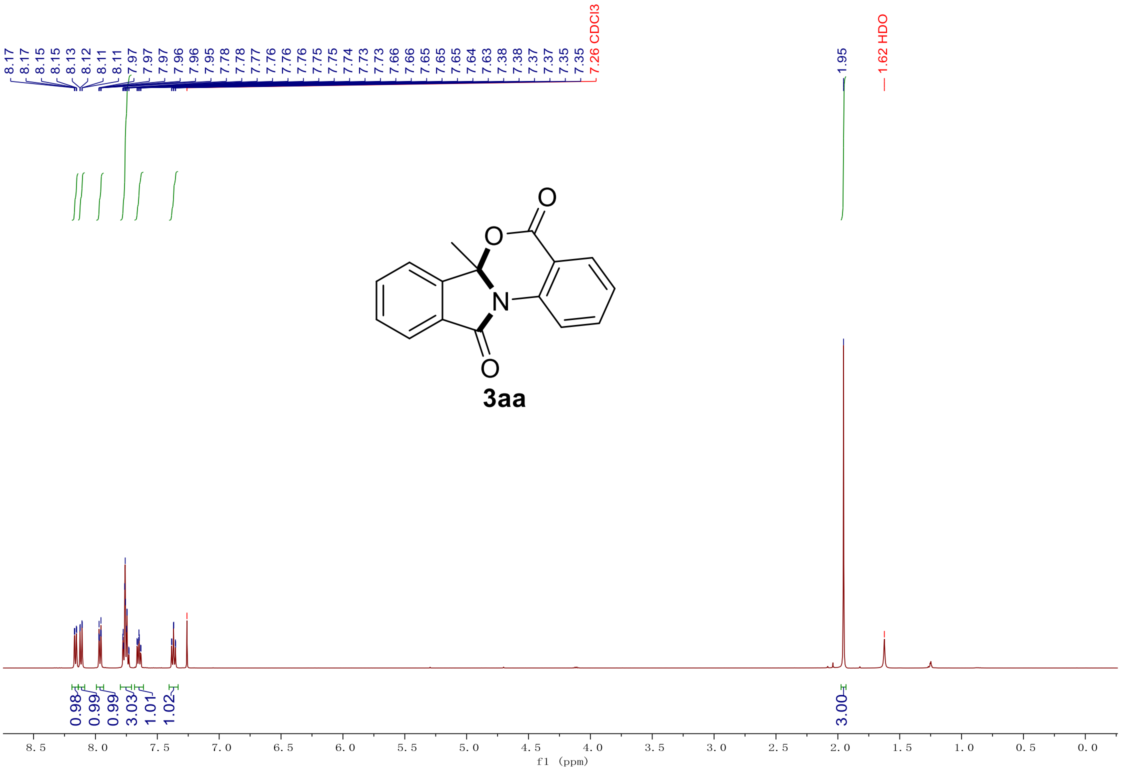

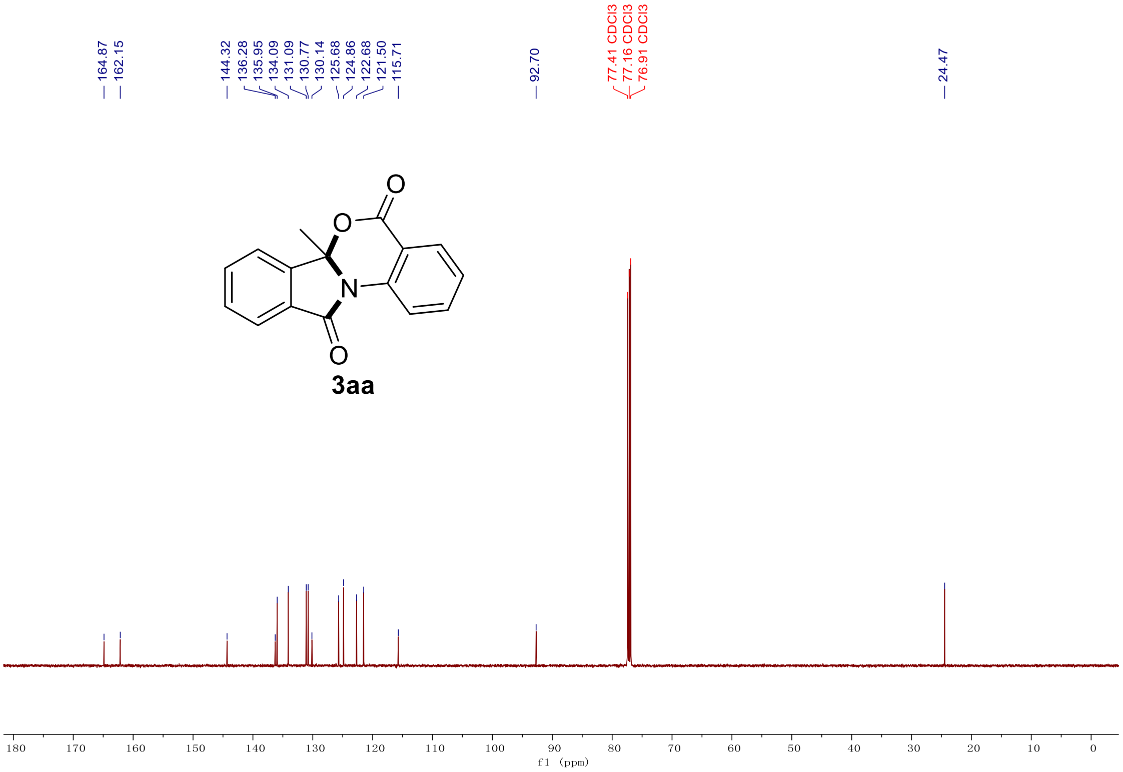

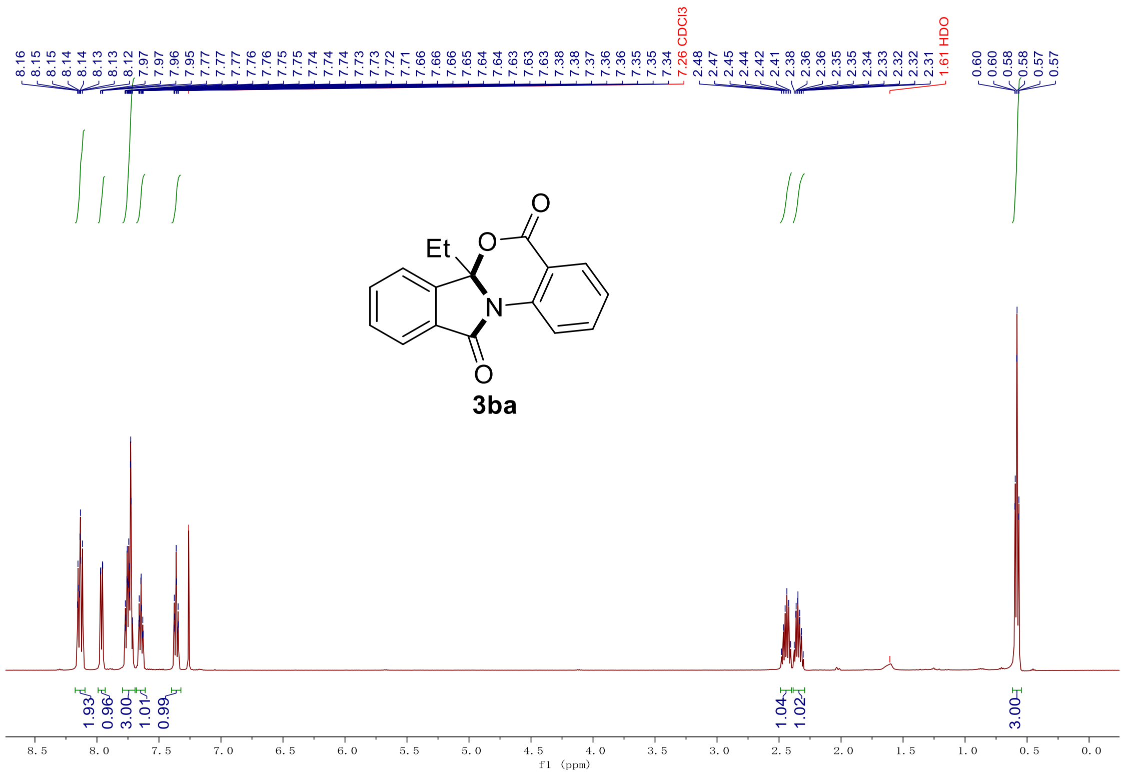

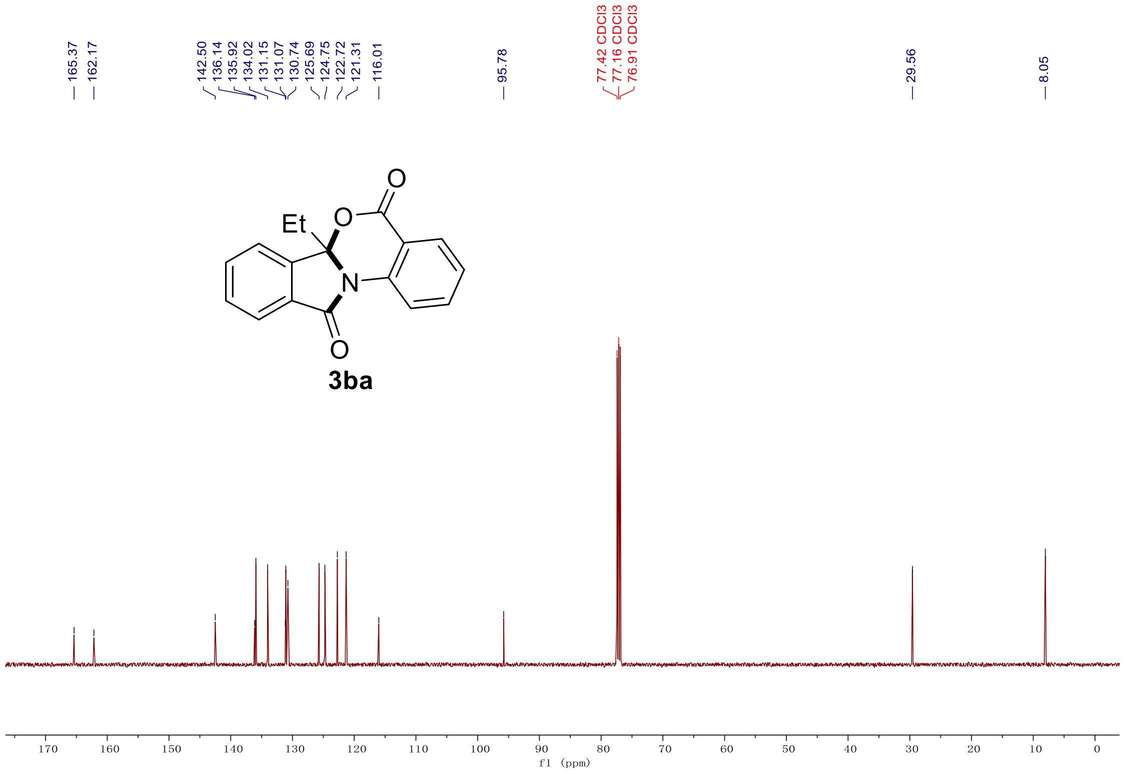

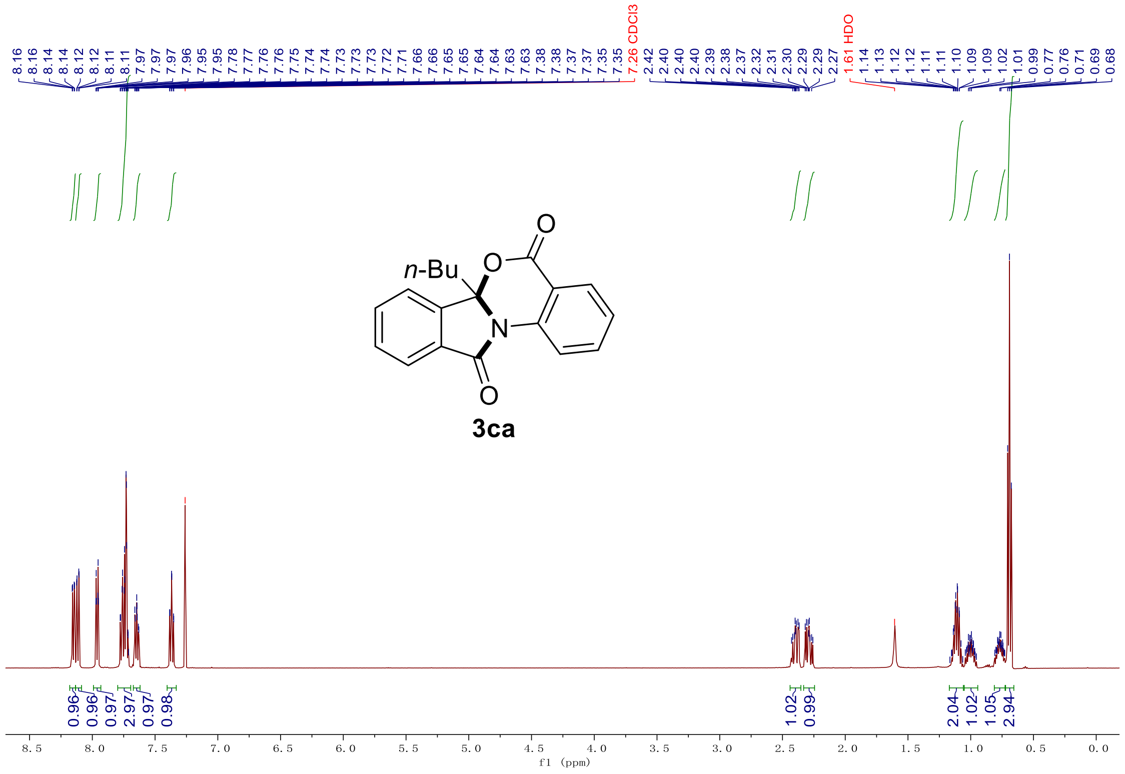
8.16 8.16 8.14 8.14 8.12 8.12 8.11 8.11 7.97 7.97 7.97 7.96 7.95 7.95 7.78 7.77 7.77 7.76 7.76 7.75 7.74 7.74 7.73 7.73 7.73 7.72 7.71 7.66 7.66 7.65 7.65 7.64 7.64 7.63 7.63 7.38 7.38 7.37 7.37 7.35
7.26 CDCl3
2.42 2.40 2.40 2.39 2.38 2.37 2.32 2.31 2.30 2.29 2.29 2.27
1.61 HDO
1.14 1.13 1.12 1.11 1.11 1.10 1.09 1.02 1.01 0.99 0.77 0.76 0.71 0.69 0.68
n-Bu
O
N
3ca
0.96 0.96 0.97 2.97 0.97 0.98
1.02 0.99
2.04 1.02 1.05 2.94
f1 (ppm)

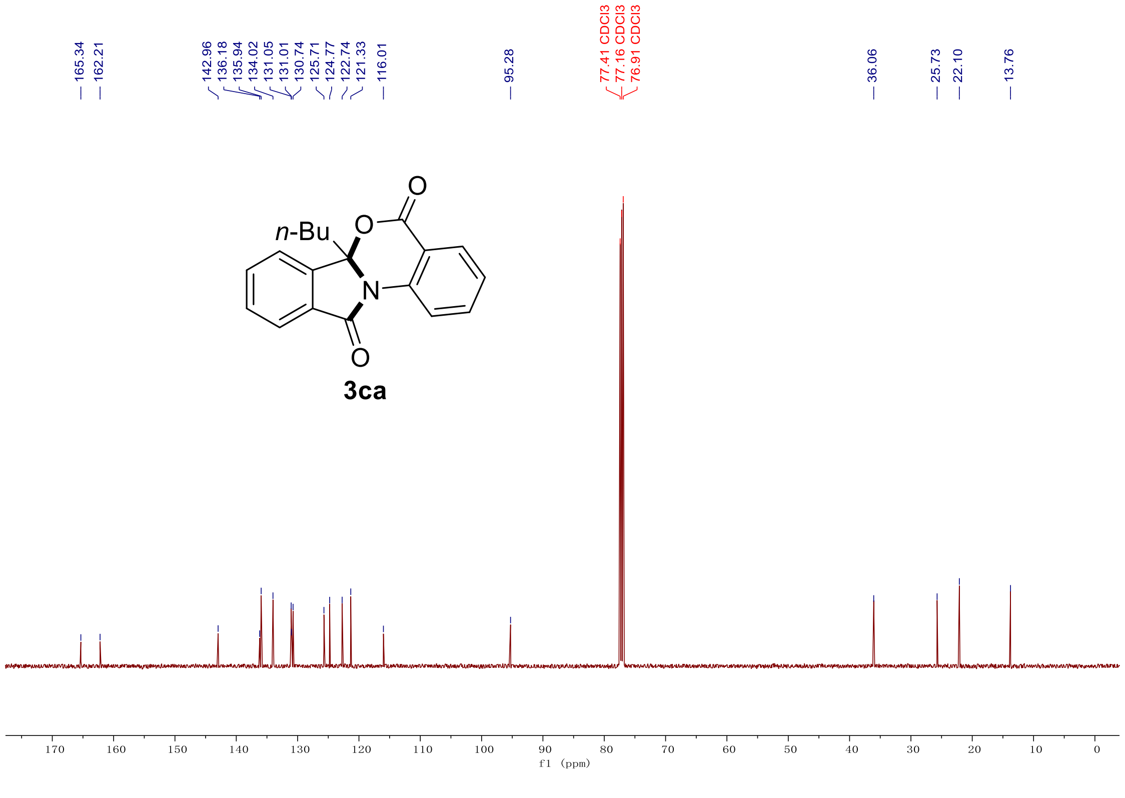
165.34 162.21
142.96 136.18 135.94 134.02 131.05 131.01 130.74 125.71 124.77 122.74 121.33 116.01
95.28
77.41 CDCl3 77.16 CDCl3 76.91 CDCl3
36.06 25.73 22.10 13.76
n-Bu
O
N
3ca
f1 (ppm)

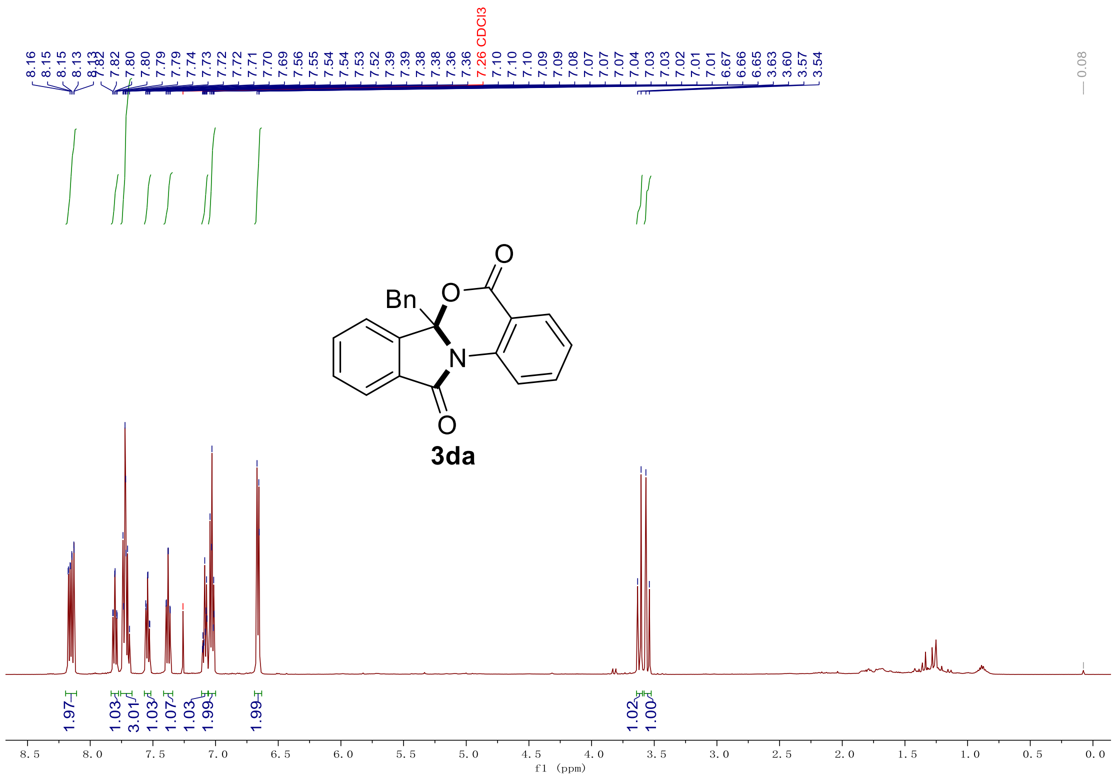

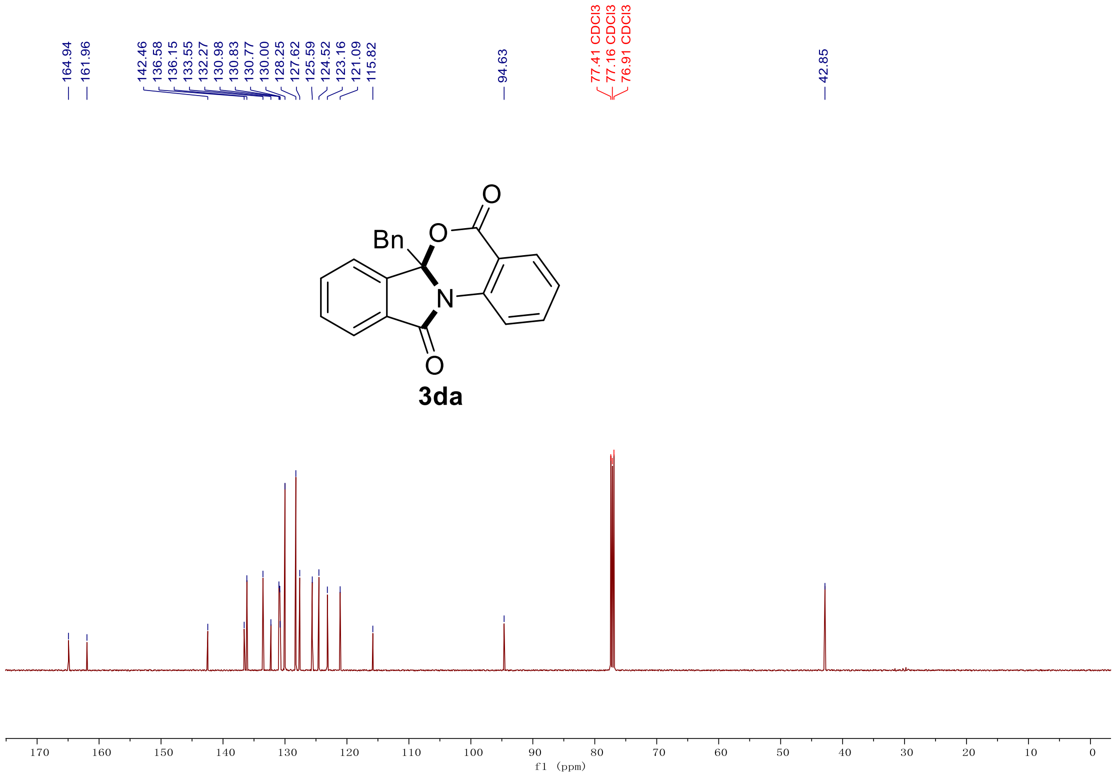

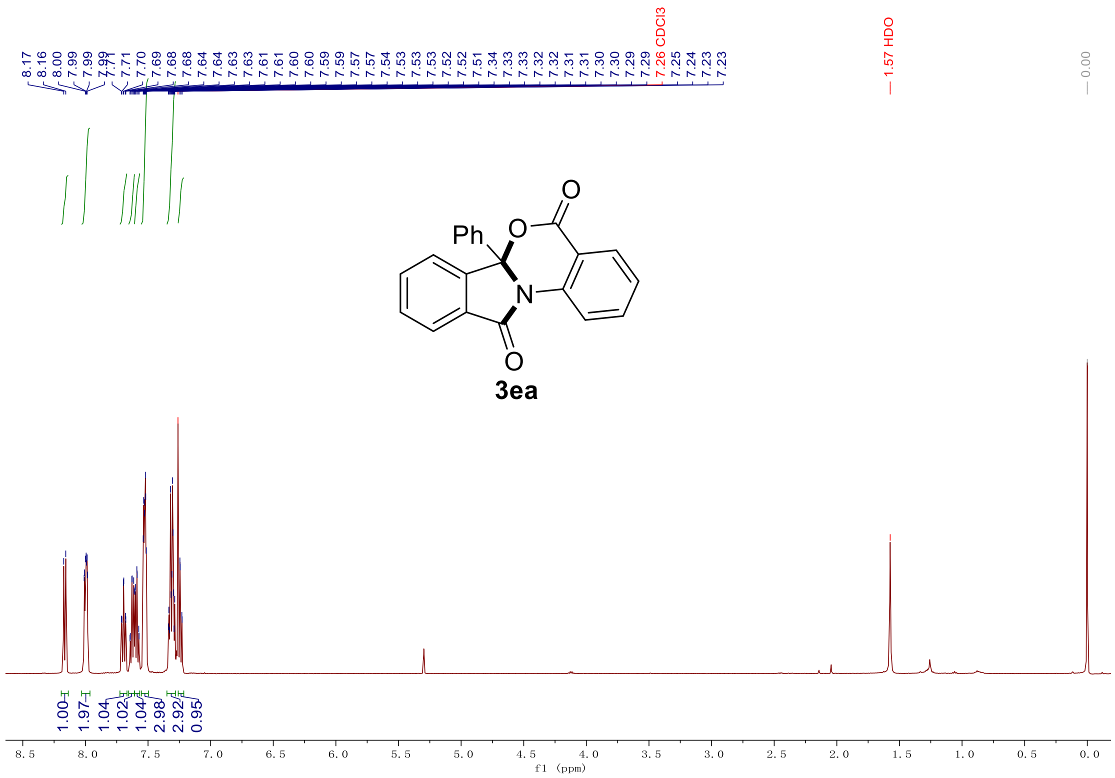

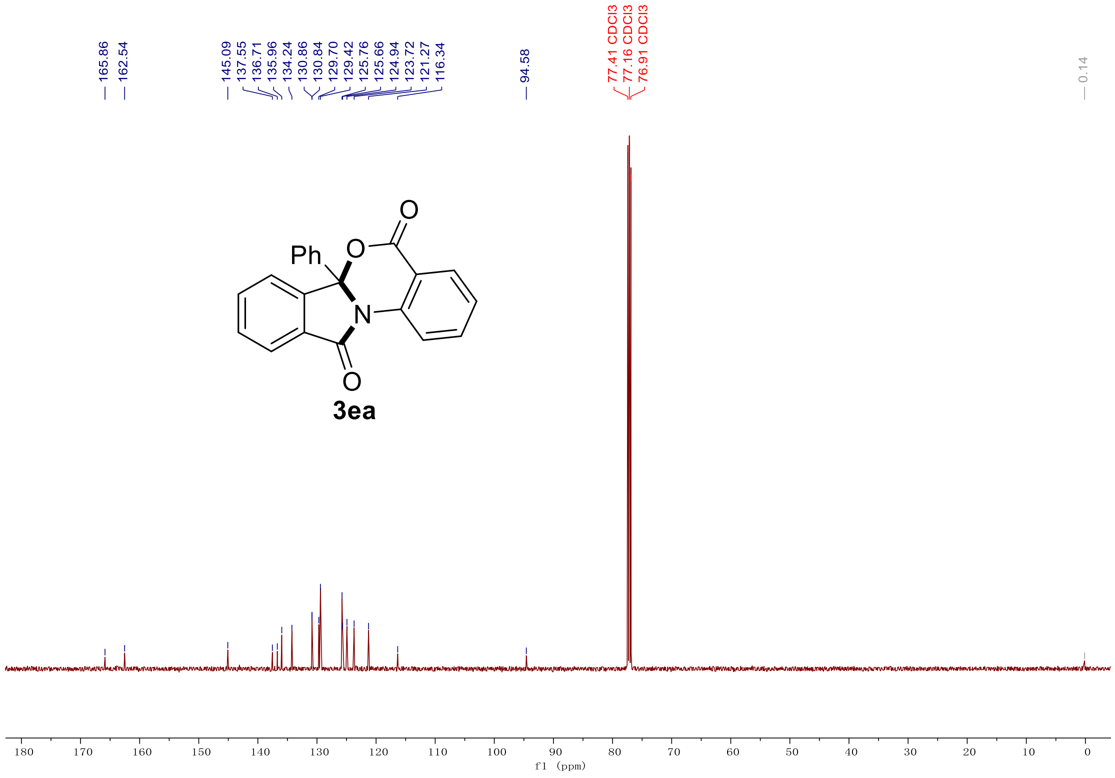

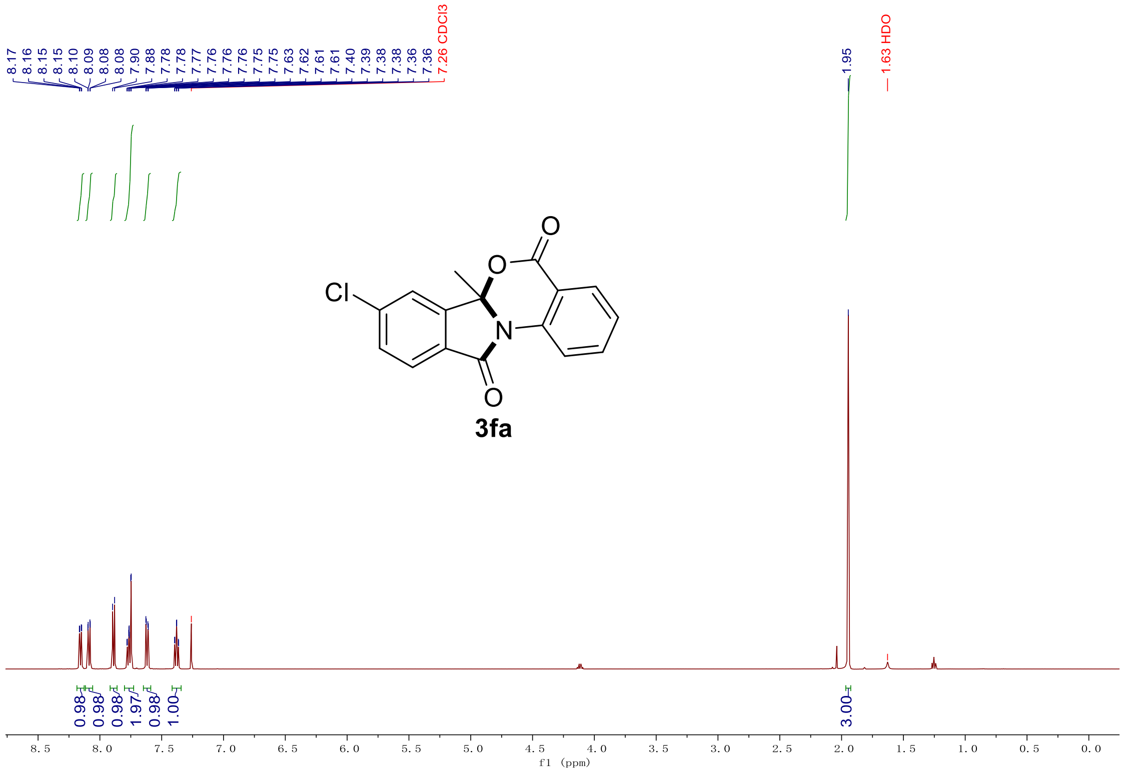

3fa

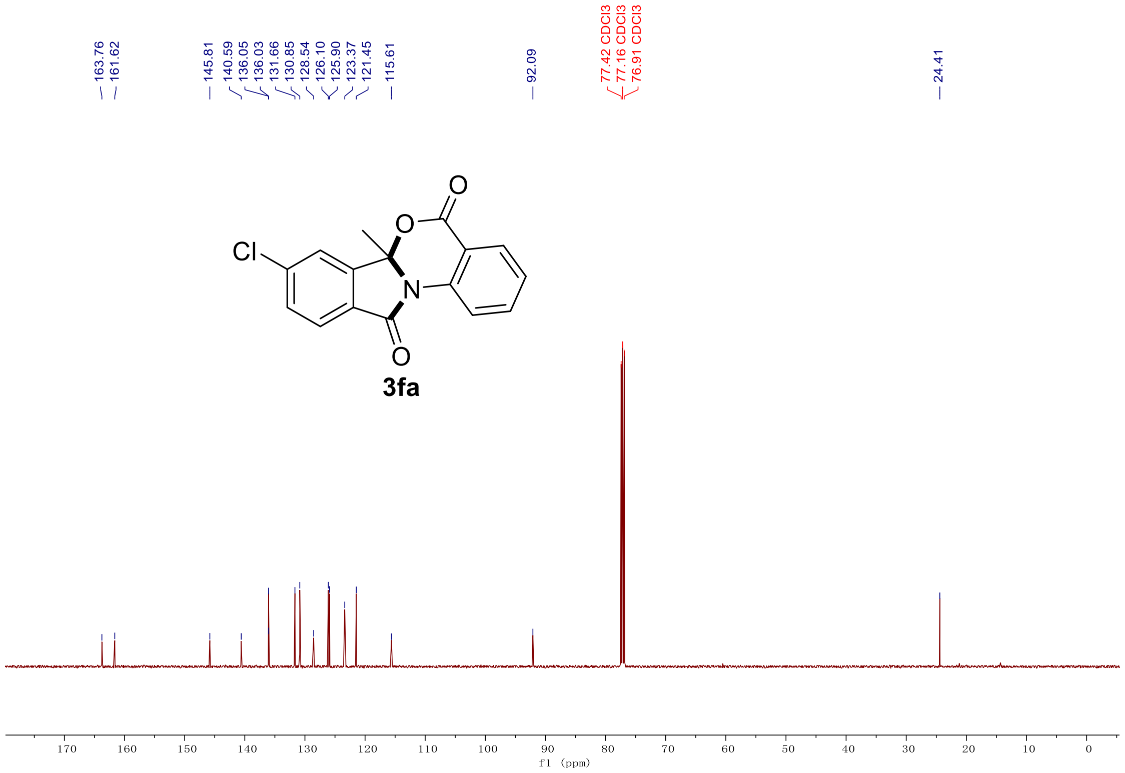

3fa

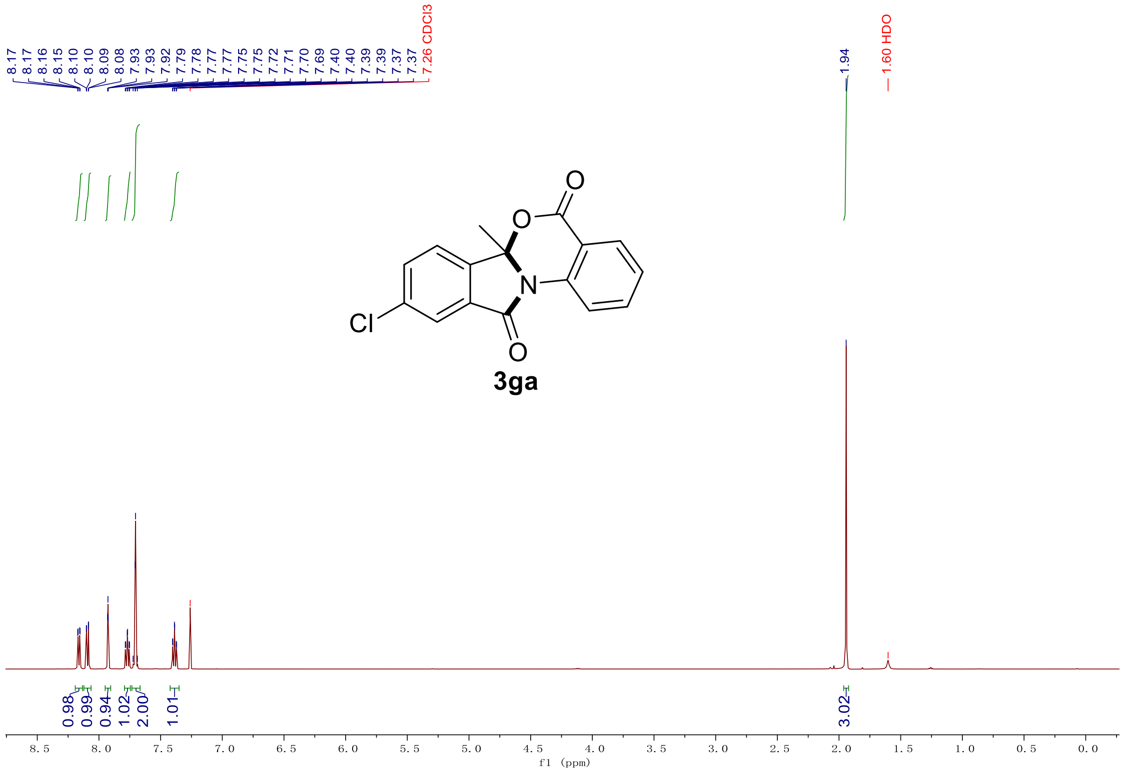

8.17
8.17
8.16
8.15
8.10
8.10
8.09
8.08
7.93
7.93
7.92
7.79
7.78
7.77
7.77
7.75
7.75
7.72
7.71
7.70
7.69
7.40
7.40
7.39
7.39
7.37
7.26 CDCl3
1.94
1.60 HDO
Cl
O
O
N
O
3ga
0.98
0.99
0.94
1.02
2.00
1.01
3.02
f1 (ppm)

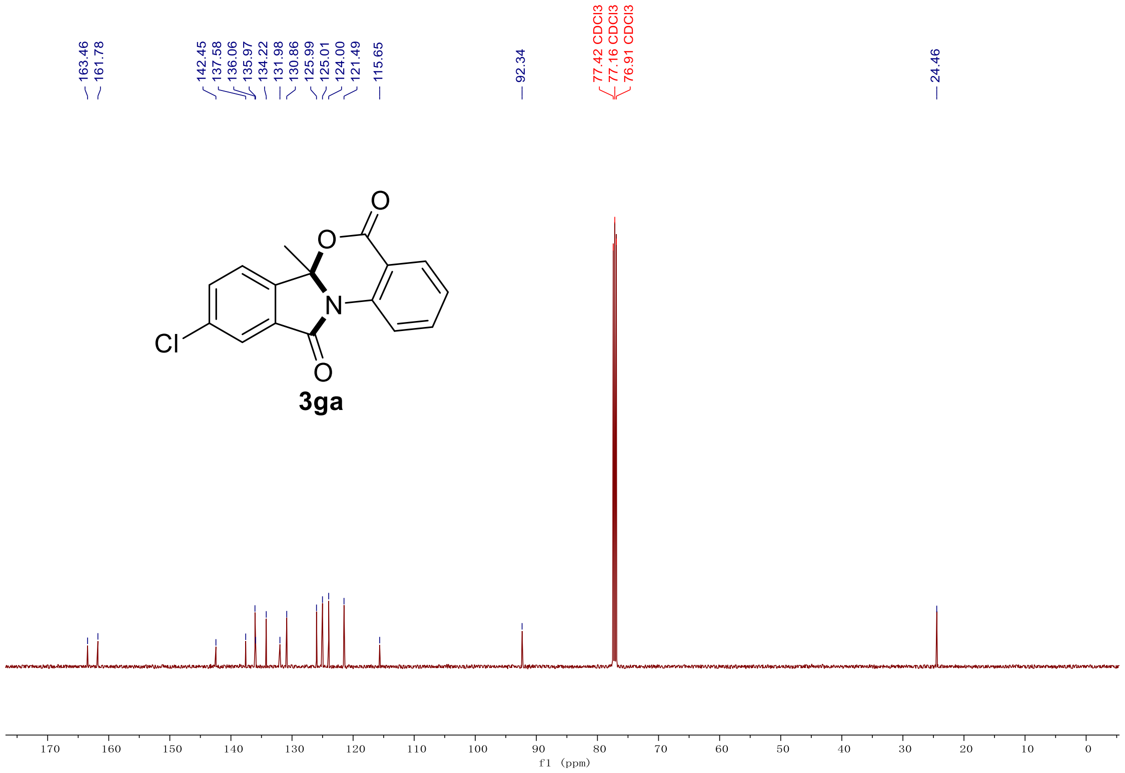

163.46
161.78
142.45
137.58
136.06
135.97
134.22
131.98
130.86
125.99
125.01
124.00
121.49
115.65
92.34
77.42 CDCl3
77.16 CDCl3
76.91 CDCl3
24.46
Cl
O
O
N
O
3ga
f1 (ppm)

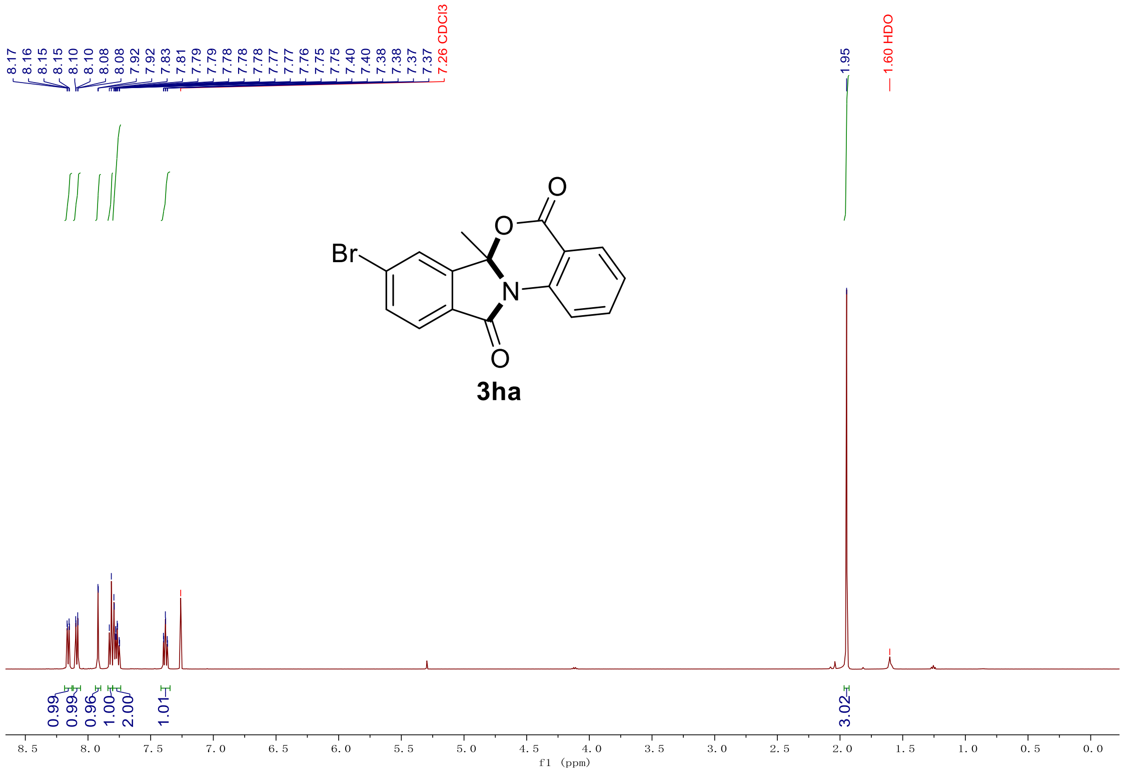
8.17
8.16
8.15
8.10
8.10
8.08
8.08
7.92
7.92
7.83
7.81
7.79
7.79
7.78
7.78
7.77
7.77
7.76
7.75
7.75
7.40
7.40
7.38
7.38
7.37
7.37
7.26 CDCl3
1.95
1.60 HDO
Br
O
O
N
O
3ha
0.99
0.99
0.96
1.00
2.00
1.01
3.02
8.5 8.0 7.5 7.0 6.5 6.0 5.5 5.0 4.5 4.0 3.5 3.0 2.5 2.0 1.5 1.0 0.5 0.0
f1 (ppm)

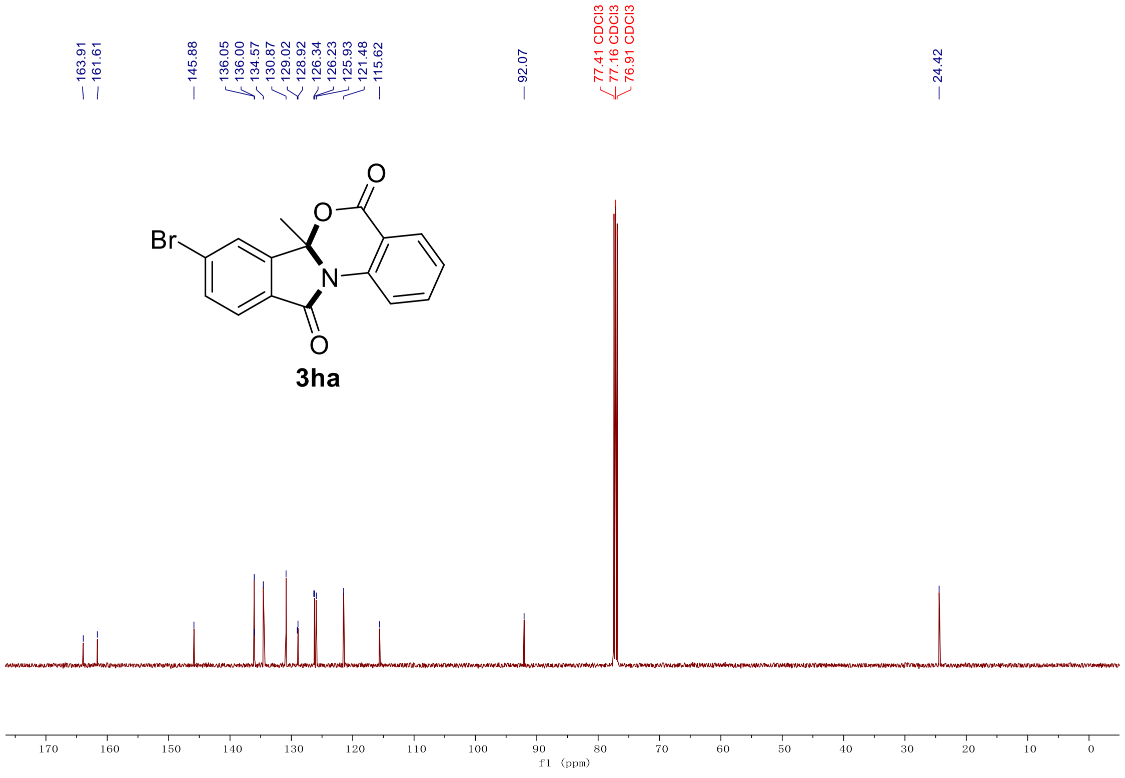
163.91
161.61
145.88
136.05
136.00
134.57
130.87
129.02
128.92
126.34
126.23
125.93
121.48
115.62
92.07
77.41 CDCl3
77.16 CDCl3
76.91 CDCl3
24.42
Br
O
O
N
O
3ha
170 160 150 140 130 120 110 100 90 80 70 60 50 40 30 20 10 0
f1 (ppm)

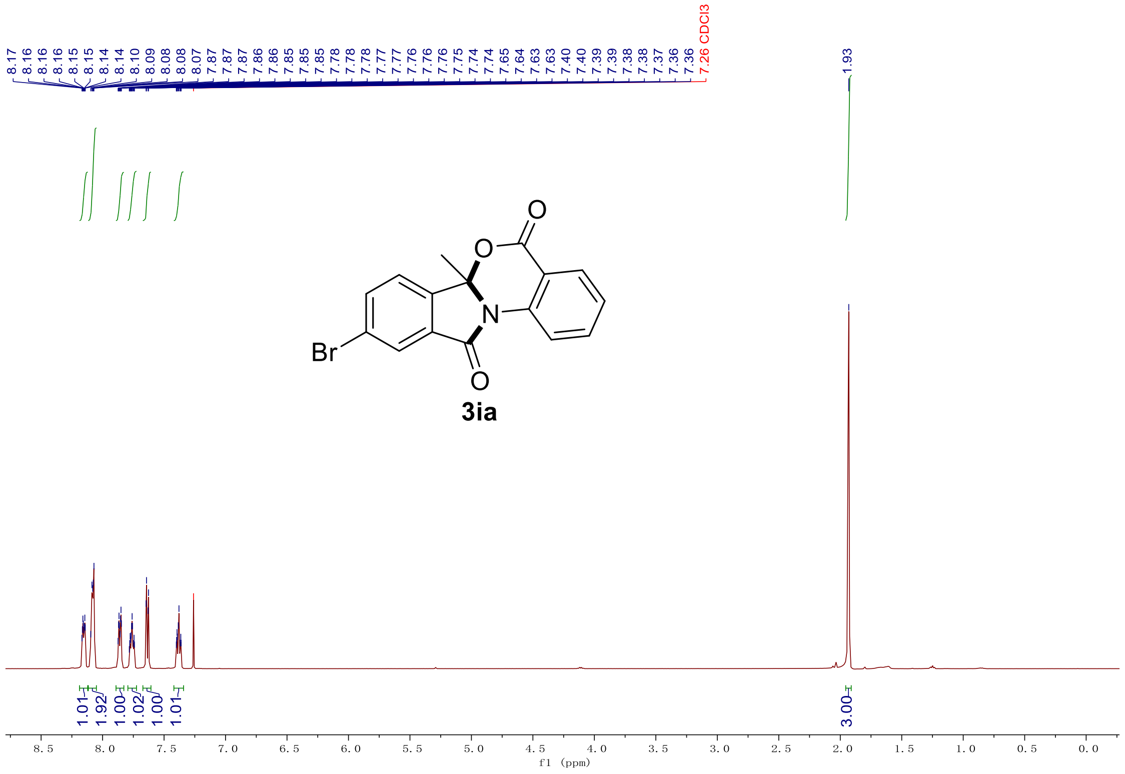

8.17 8.16 8.16 8.16 8.15 8.15 8.14 8.14 8.10 8.09 8.08 8.08 8.07 7.87 7.87 7.87 7.86 7.86 7.85 7.85 7.85 7.78 7.78 7.78 7.77 7.77 7.76 7.76 7.76 7.75 7.74 7.74 7.65 7.64 7.63 7.63 7.40 7.40 7.39 7.39 7.38 7.38 7.37 7.36 7.36
7.26 CDCl3
1.93
Br
O
N
3ia
1.01
1.92
1.00
1.02
1.00
1.01
3.00
f1 (ppm)

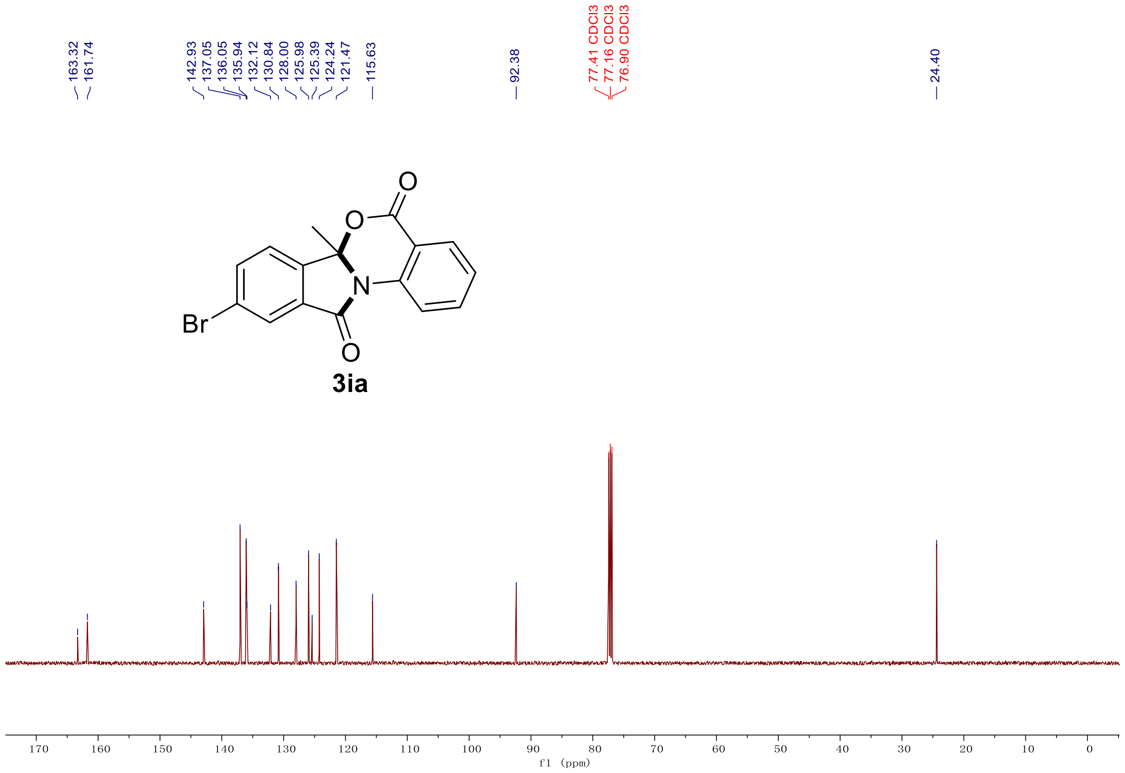

163.32
161.74
142.93
137.05
136.05
135.94
132.12
130.84
128.00
125.98
125.39
124.24
121.47
115.63
92.38
77.41 CDCl3
77.16 CDCl3
76.90 CDCl3
24.40
Br
O
N
3ia
f1 (ppm)

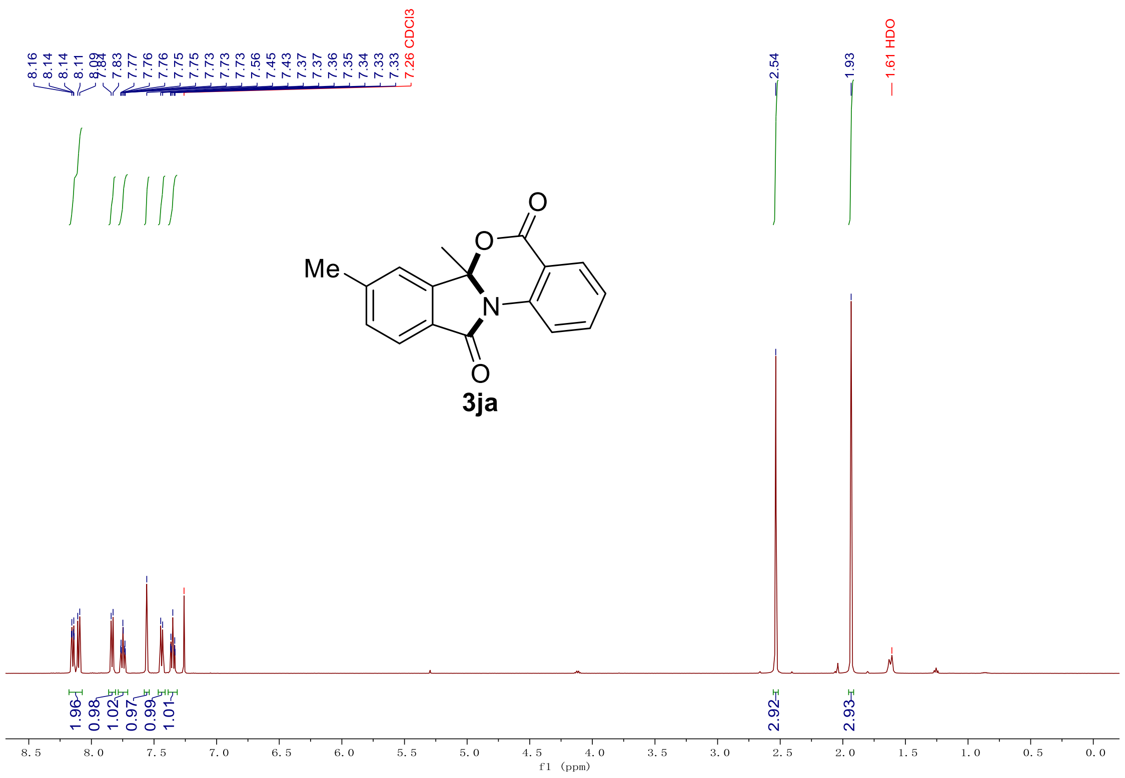

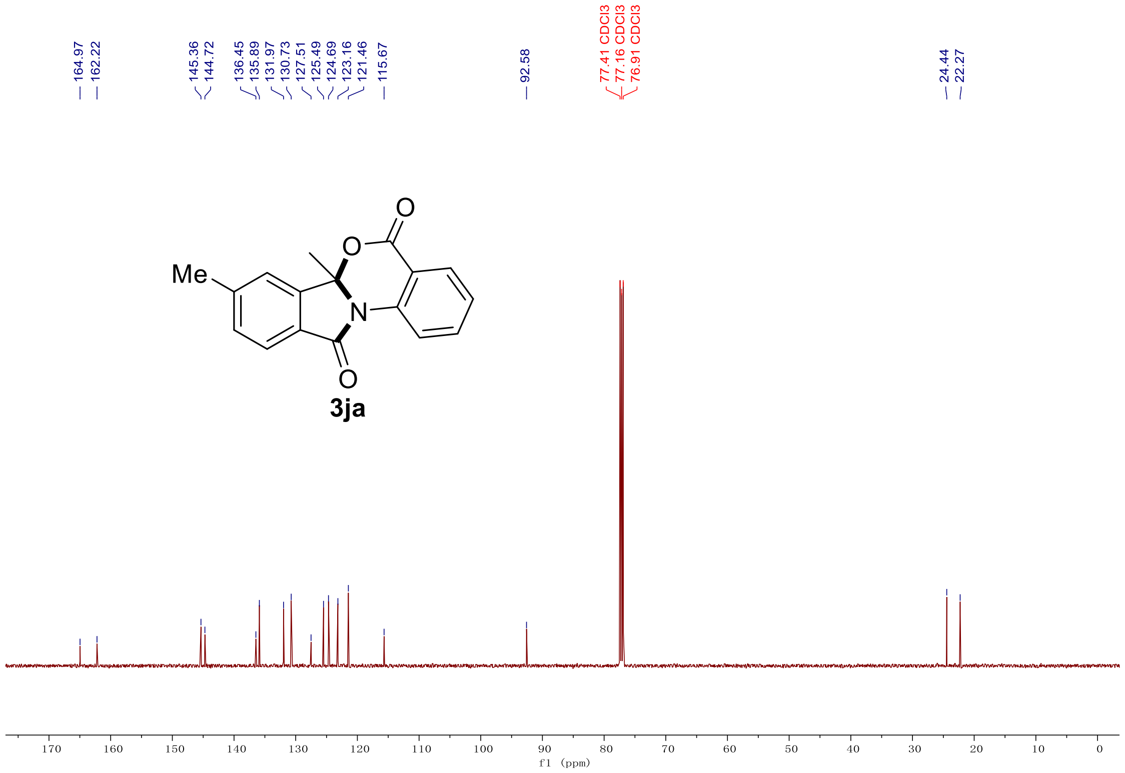

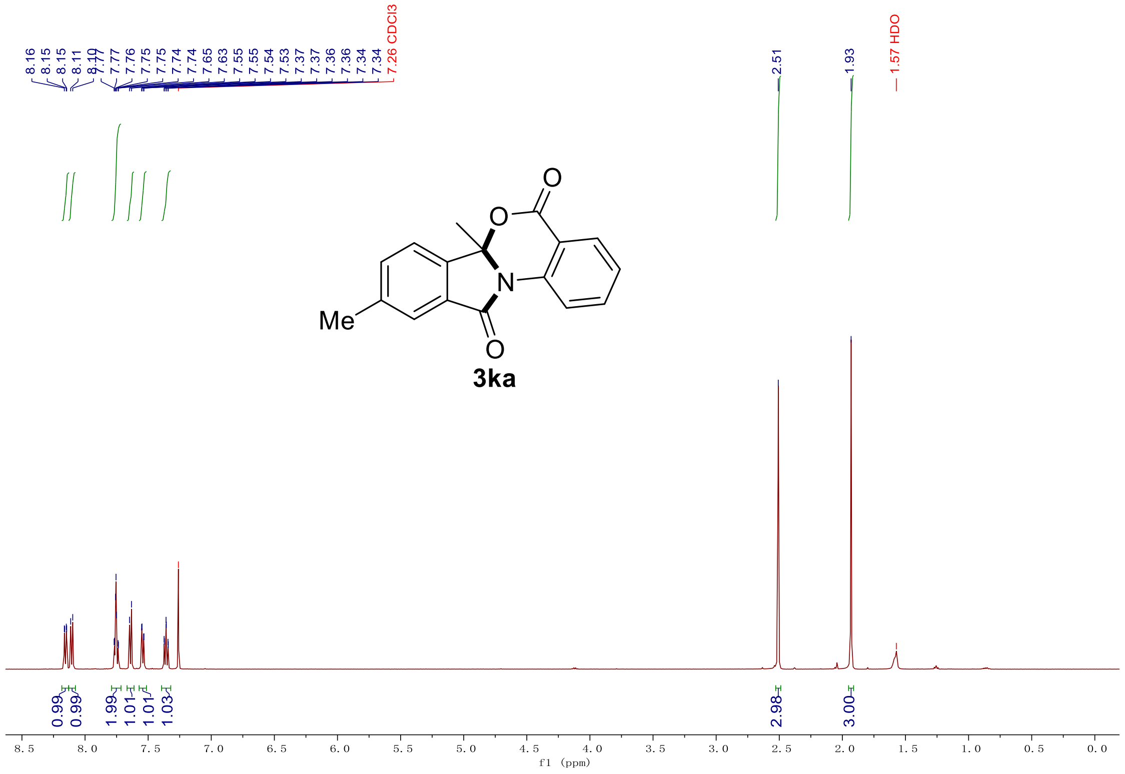

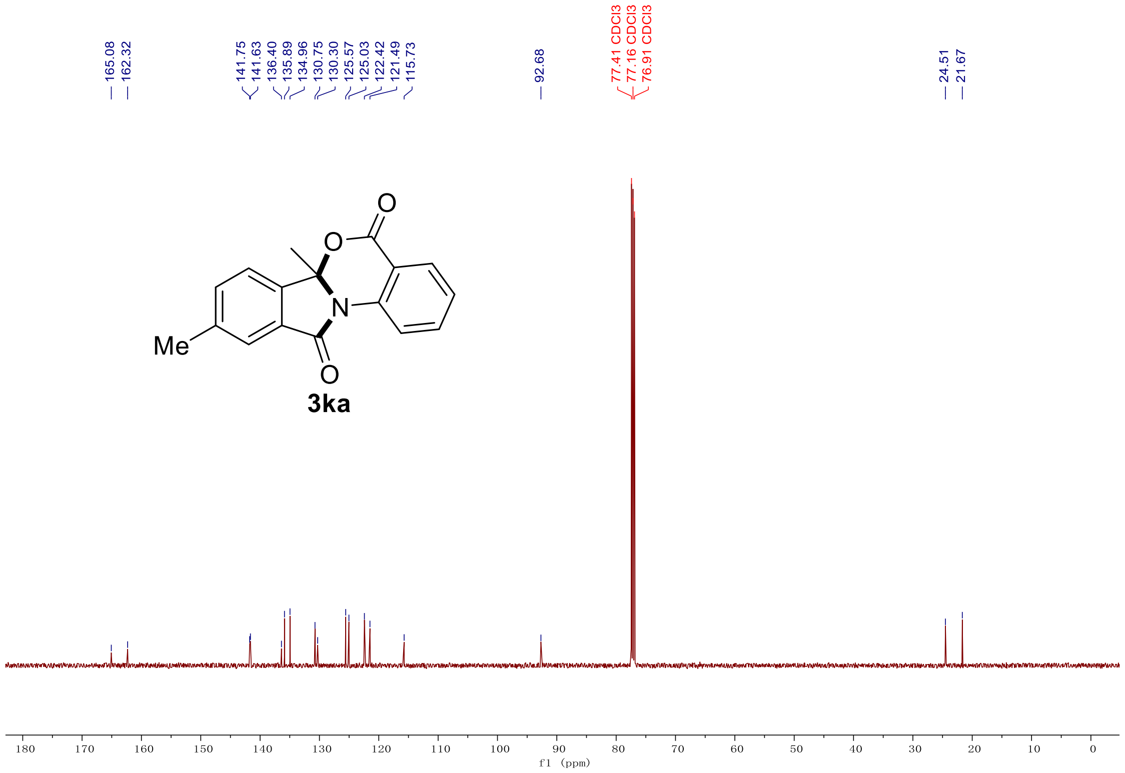

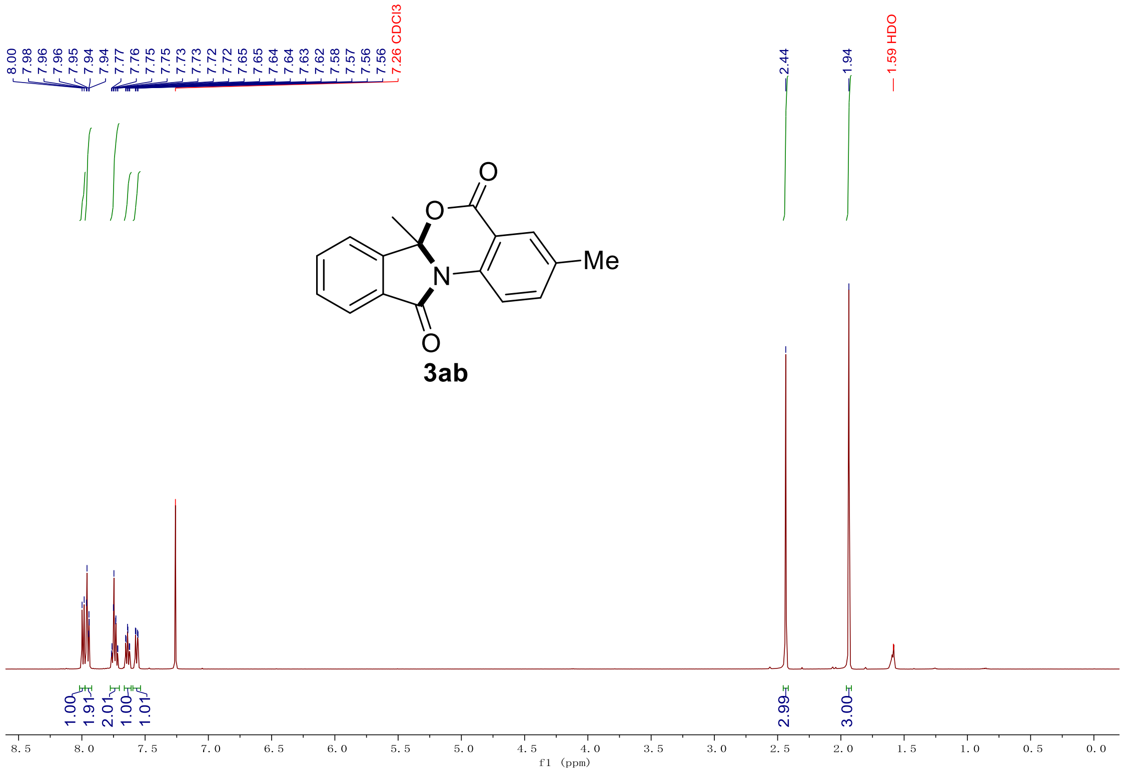
8.00
7.98
7.96
7.96
7.95
7.94
7.94
7.77
7.76
7.75
7.75
7.73
7.73
7.72
7.72
7.65
7.65
7.64
7.64
7.63
7.62
7.58
7.57
7.56
7.56
7.26 CDCl3
2.44
1.94
1.59 HDO
Me
3ab
1.00
1.91
2.01
1.00
1.01
2.99
3.00
f1 (ppm)

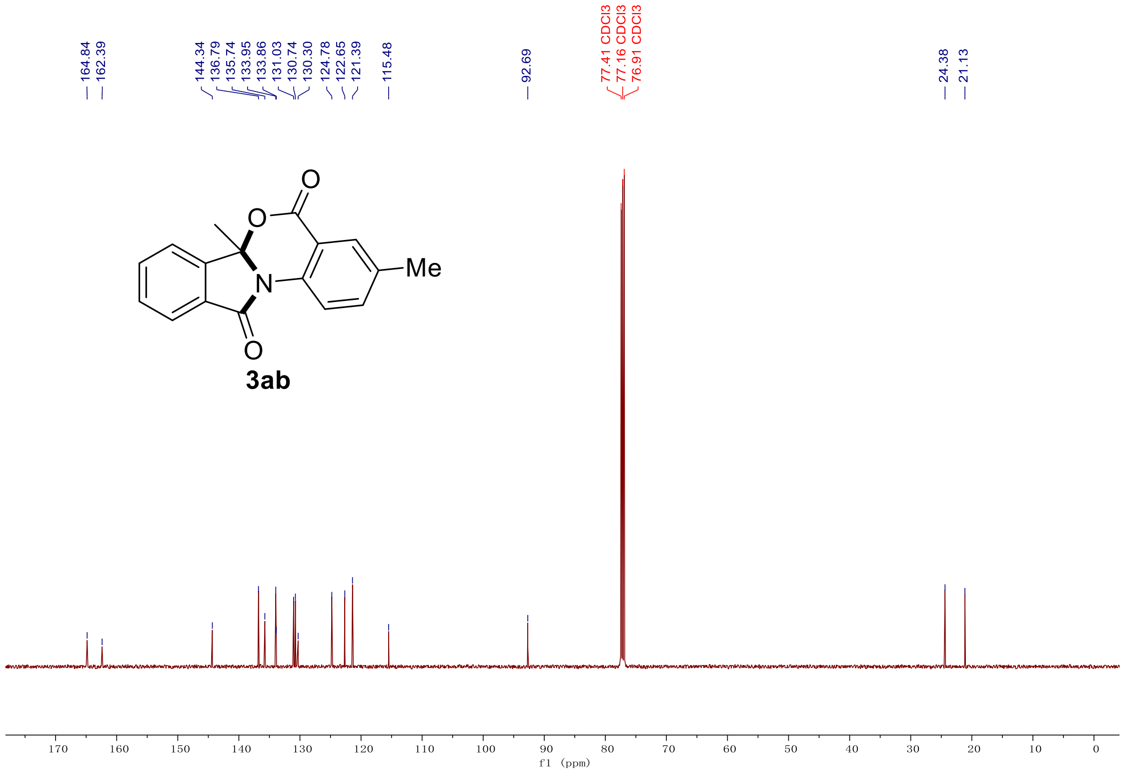
164.84
162.39
144.34
136.79
135.74
133.95
133.86
131.03
130.74
130.30
124.78
122.65
121.39
115.48
92.69
77.41 CDCl3
77.16 CDCl3
76.91 CDCl3
24.38
21.13
Me
3ab
f1 (ppm)

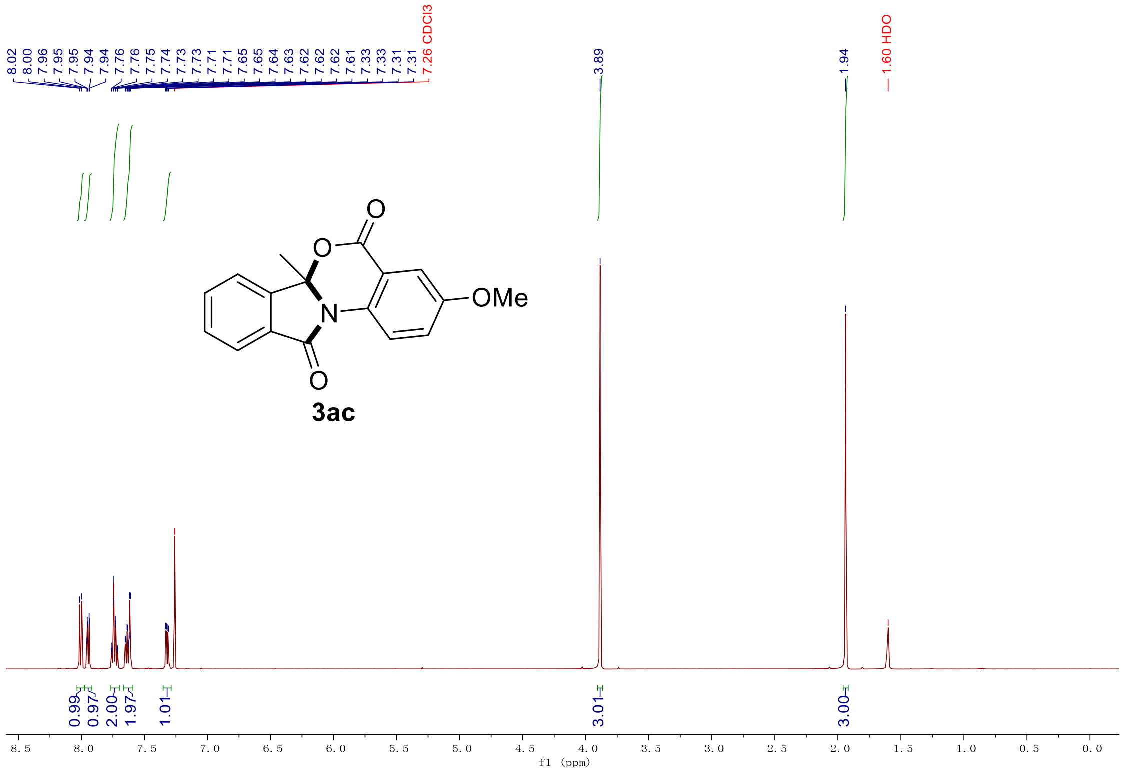

8.02
8.00
7.96
7.95
7.95
7.94
7.94
7.76
7.76
7.75
7.74
7.73
7.73
7.71
7.71
7.65
7.64
7.63
7.62
7.62
7.61
7.33
7.33
7.31
7.31
7.26 CDCl3
3.89
1.94
1.60 HDO
O
O
N
O
OMe
3ac
0.99
0.97
2.00
1.97
1.01
3.01
3.00
f1 (ppm)

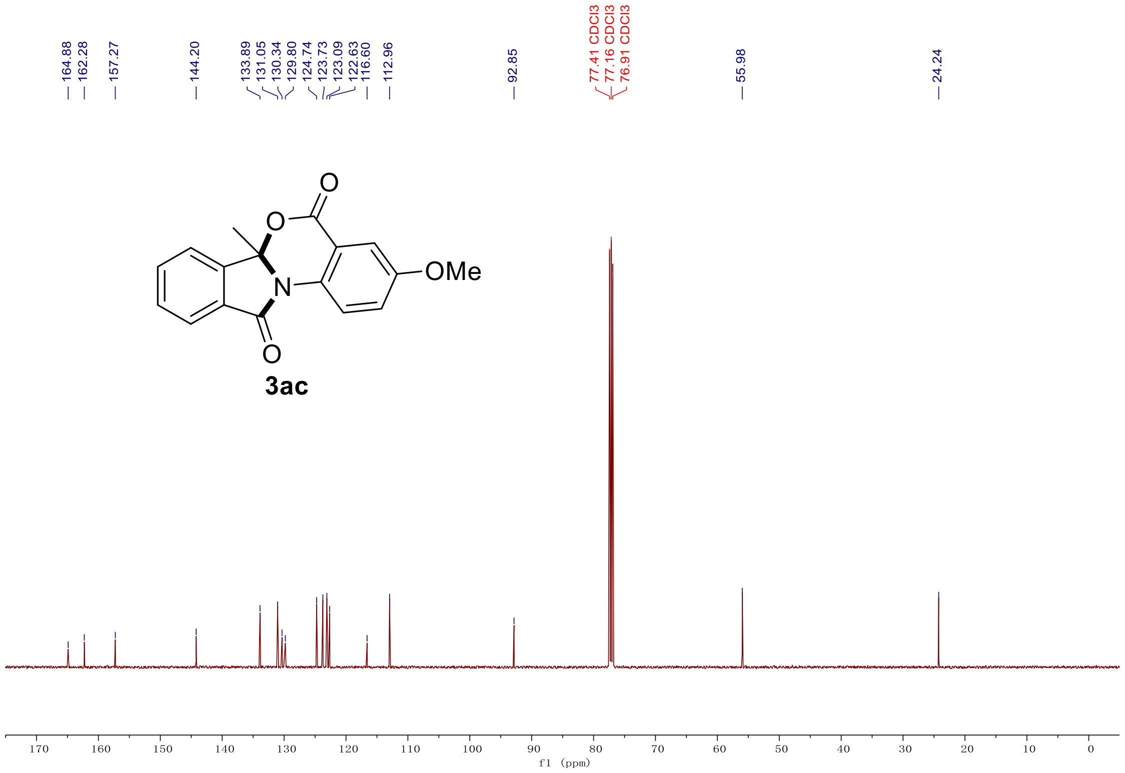

164.88
162.28
157.27
144.20
133.89
131.05
130.34
129.80
124.74
123.73
123.09
122.63
116.60
112.96
92.85
77.41 CDCl3
77.16 CDCl3
76.91 CDCl3
55.98
24.24
O
O
N
O
OMe
3ac
f1 (ppm)

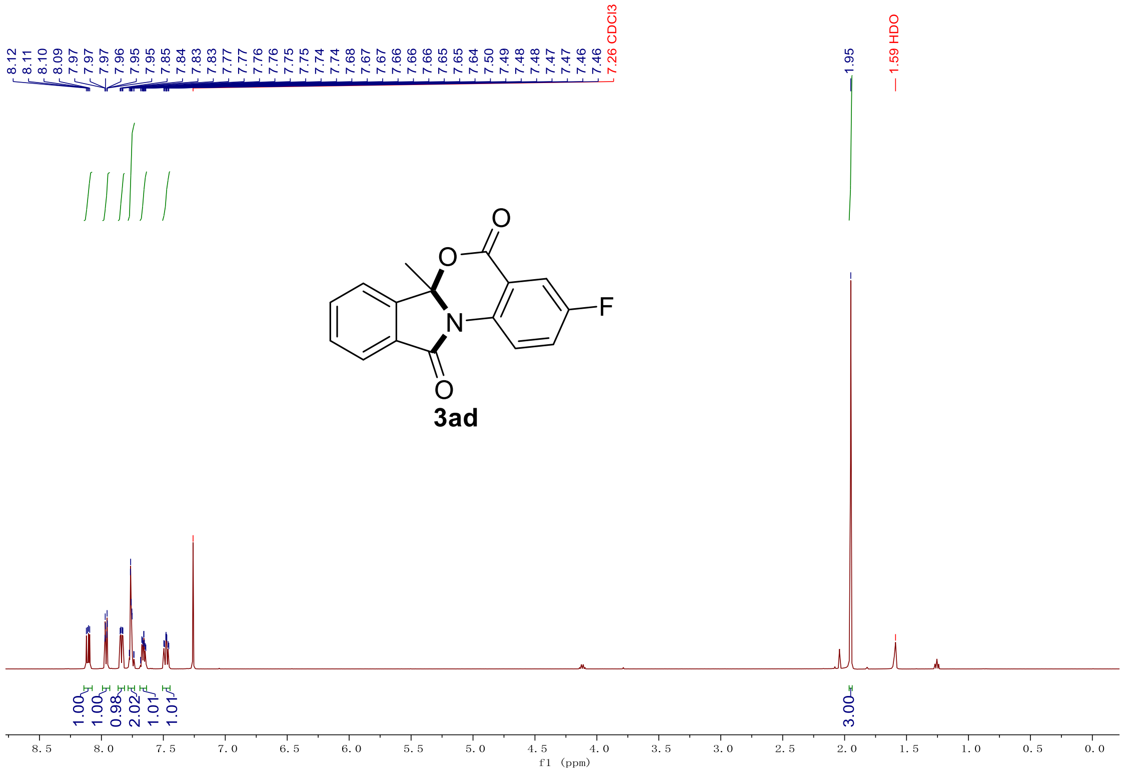
8.12 8.11 8.10 8.09 7.97 7.97 7.97 7.96 7.95 7.95 7.85 7.84 7.83 7.83 7.77 7.77 7.76 7.76 7.75 7.75 7.74 7.74 7.68 7.67 7.67 7.66 7.66 7.66 7.65 7.65 7.64 7.50 7.49 7.48 7.48 7.47 7.47 7.46 7.46
7.26 CDCl3
1.95
1.59 HDO
3ad
1.00 1.00 0.98 2.02 1.01 1.01
3.00
f1 (ppm)

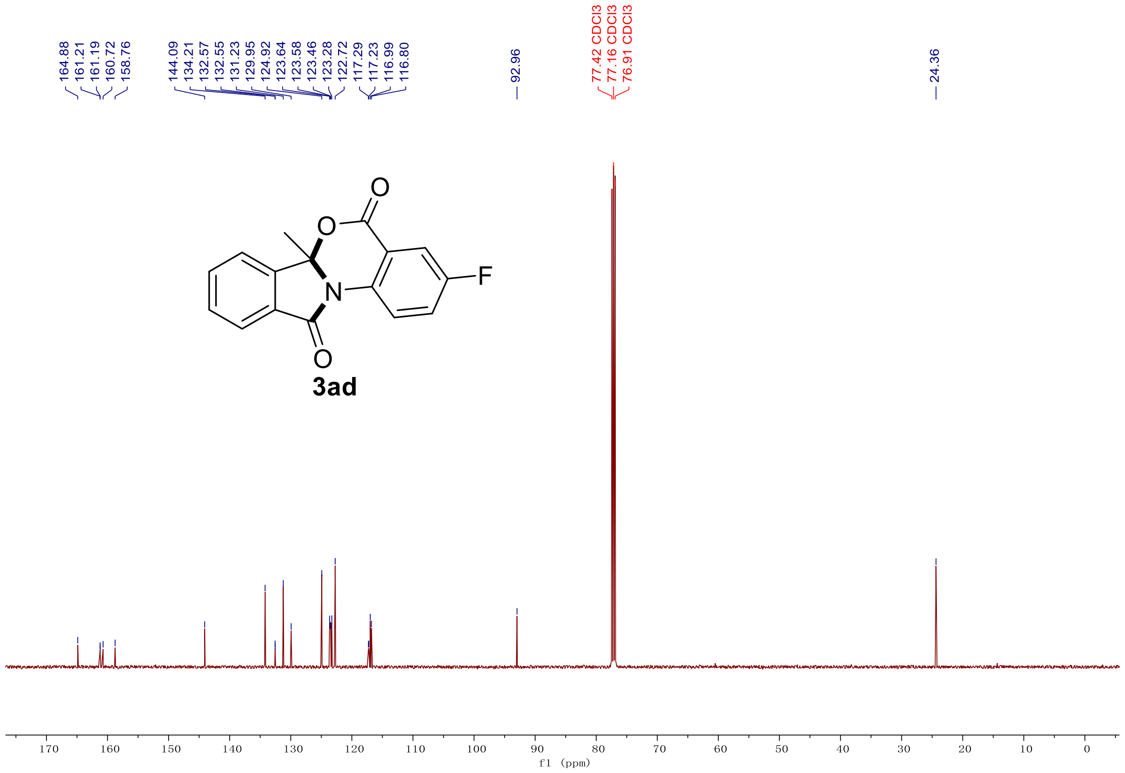
164.88 161.21 161.19 160.72 158.76
144.09 134.21 132.57 132.55 131.23 129.95 124.92 123.64 123.58 123.46 123.28 122.72 117.29 117.23 116.99 116.80
92.96
77.42 CDCl3
77.16 CDCl3
76.91 CDCl3
24.36
3ad
f1 (ppm)

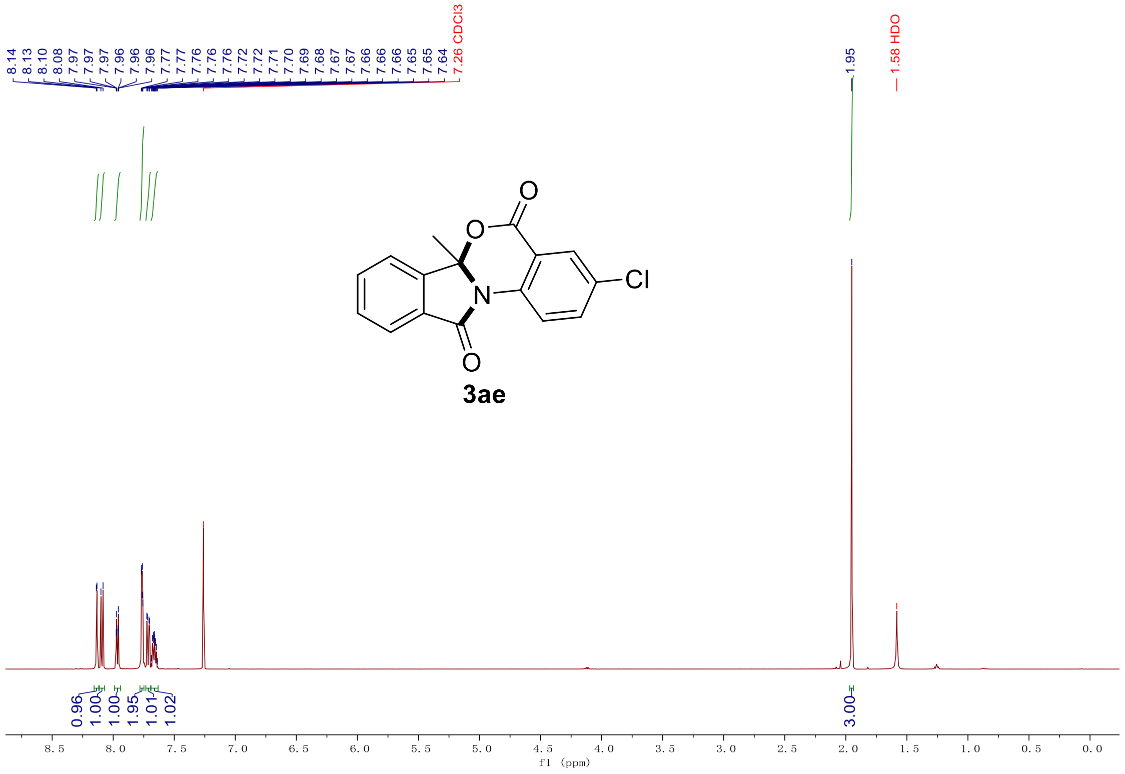
8.14
8.13
8.10
8.08
7.97
7.97
7.97
7.96
7.96
7.96
7.77
7.77
7.76
7.76
7.72
7.72
7.71
7.70
7.69
7.68
7.67
7.67
7.66
7.66
7.66
7.65
7.65
7.64
7.26 CDCl3
1.95
1.58 HDO
O
O
N
Cl
O
3ae
0.96
1.00
1.00
1.95
1.01
1.02
3.00
8.5 8.0 7.5 7.0 6.5 6.0 5.5 5.0 4.5 4.0 3.5 3.0 2.5 2.0 1.5 1.0 0.5 0.0
f1 (ppm)

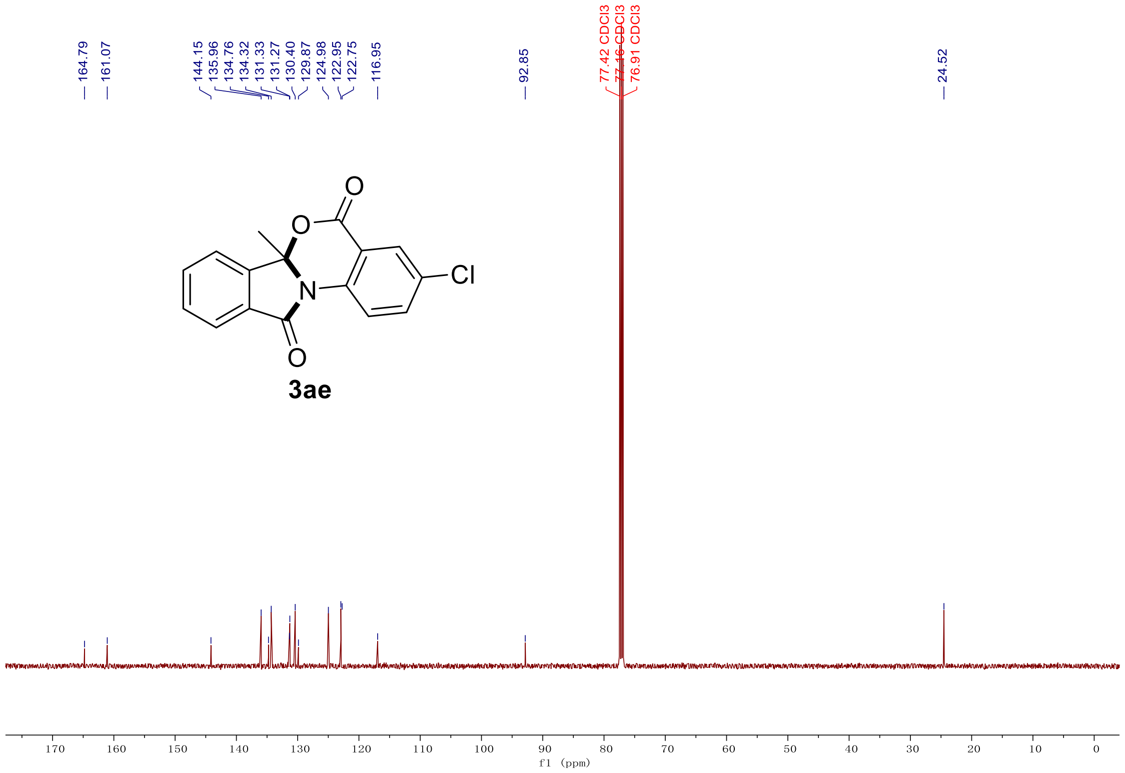
164.79
161.07
144.15
135.96
134.76
134.32
131.33
131.27
130.40
129.87
124.98
122.95
122.75
116.95
92.85
77.42 CDCl3
77.16 CDCl3
76.91 CDCl3
24.52
O
O
N
Cl
O
3ae
170 160 150 140 130 120 110 100 90 80 70 60 50 40 30 20 10 0
f1 (ppm)

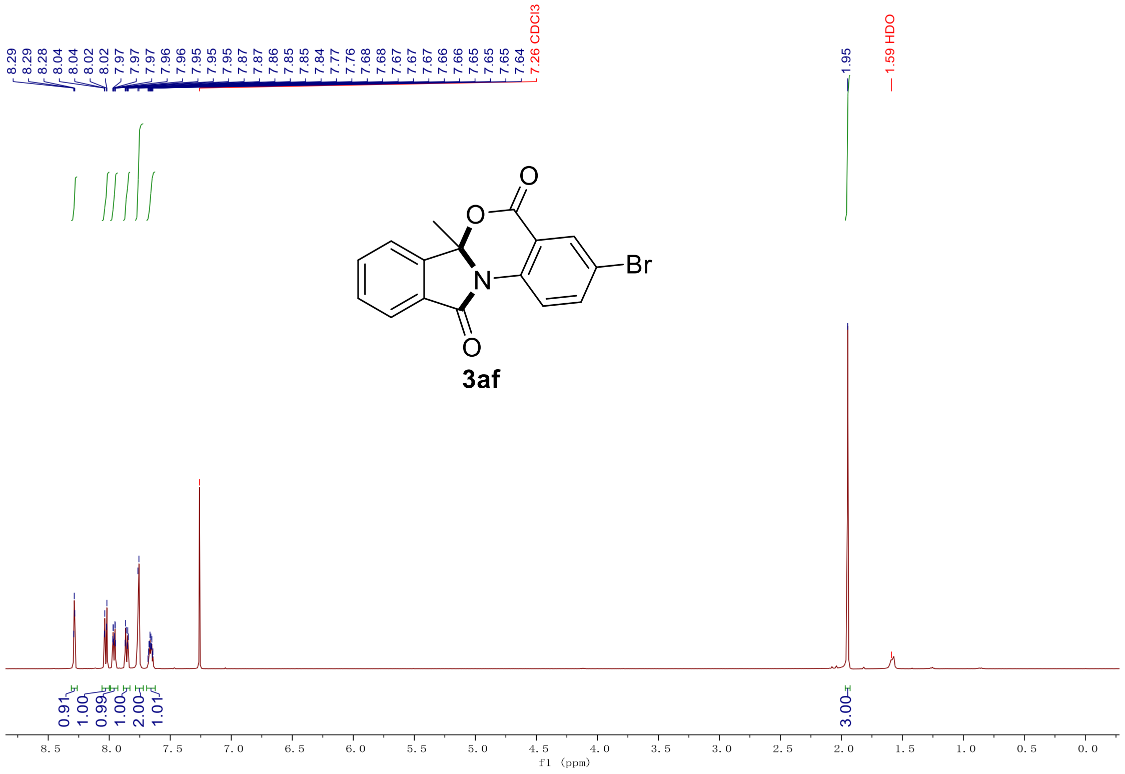
8.29
8.29
8.28
8.04
8.04
8.02
8.02
7.97
7.97
7.97
7.96
7.96
7.95
7.95
7.95
7.87
7.87
7.86
7.85
7.85
7.84
7.77
7.76
7.68
7.68
7.67
7.67
7.67
7.66
7.66
7.65
7.65
7.64
7.26 CDCl3
1.95
1.59 HDO
O
O
N
Br
O
3af
0.91
1.00
0.99
1.00
2.00
1.01
3.00
f1 (ppm)

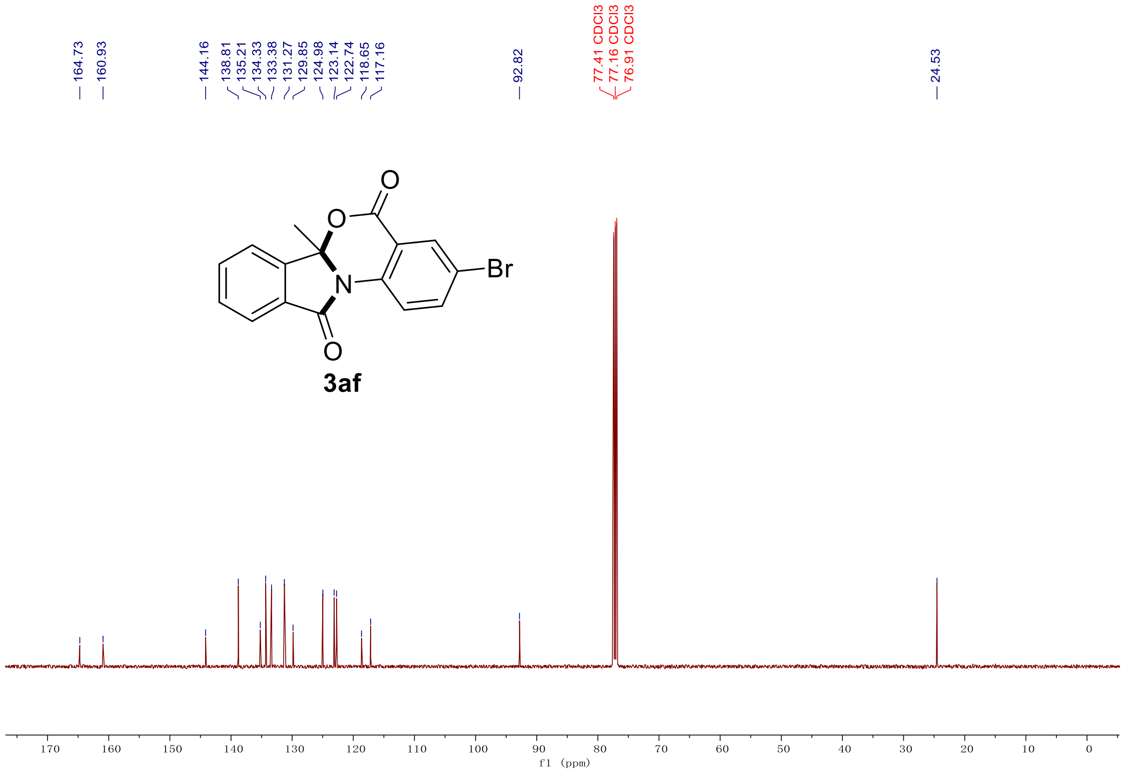
164.73
160.93
144.16
138.81
135.21
134.33
133.38
131.27
129.85
124.98
123.14
122.74
118.65
117.16
92.82
77.41 CDCl3
77.16 CDCl3
76.91 CDCl3
24.53
O
O
N
Br
O
3af
f1 (ppm)

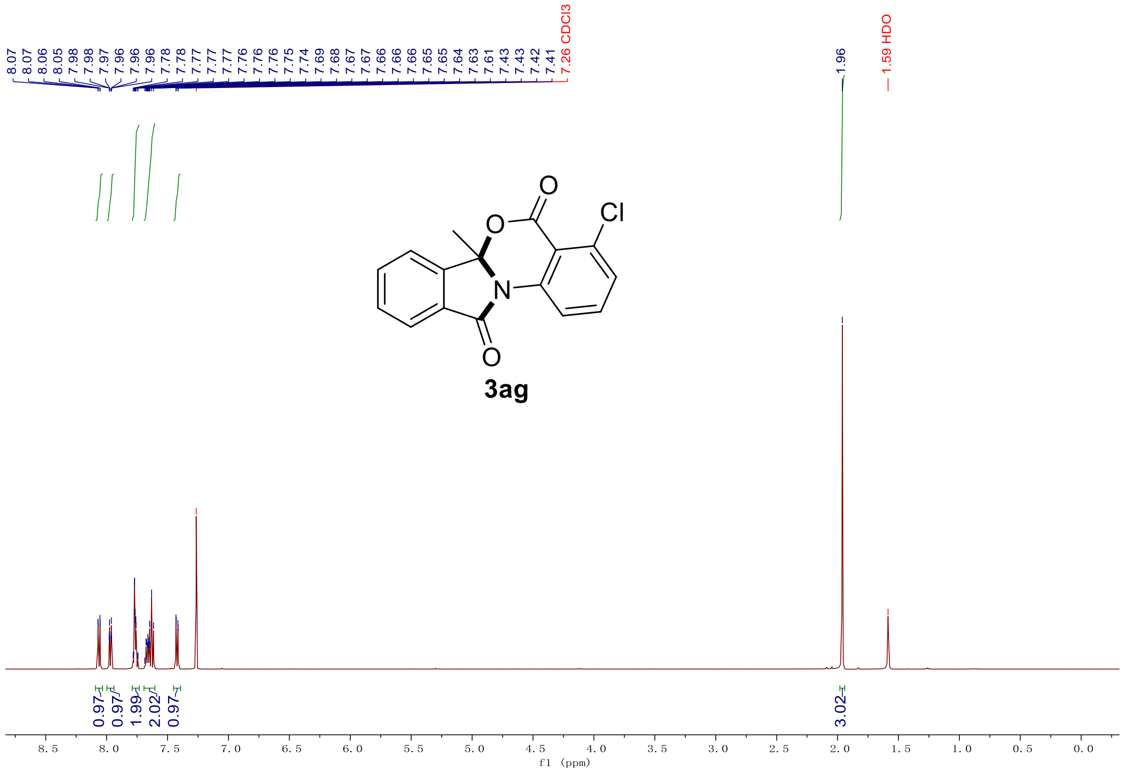

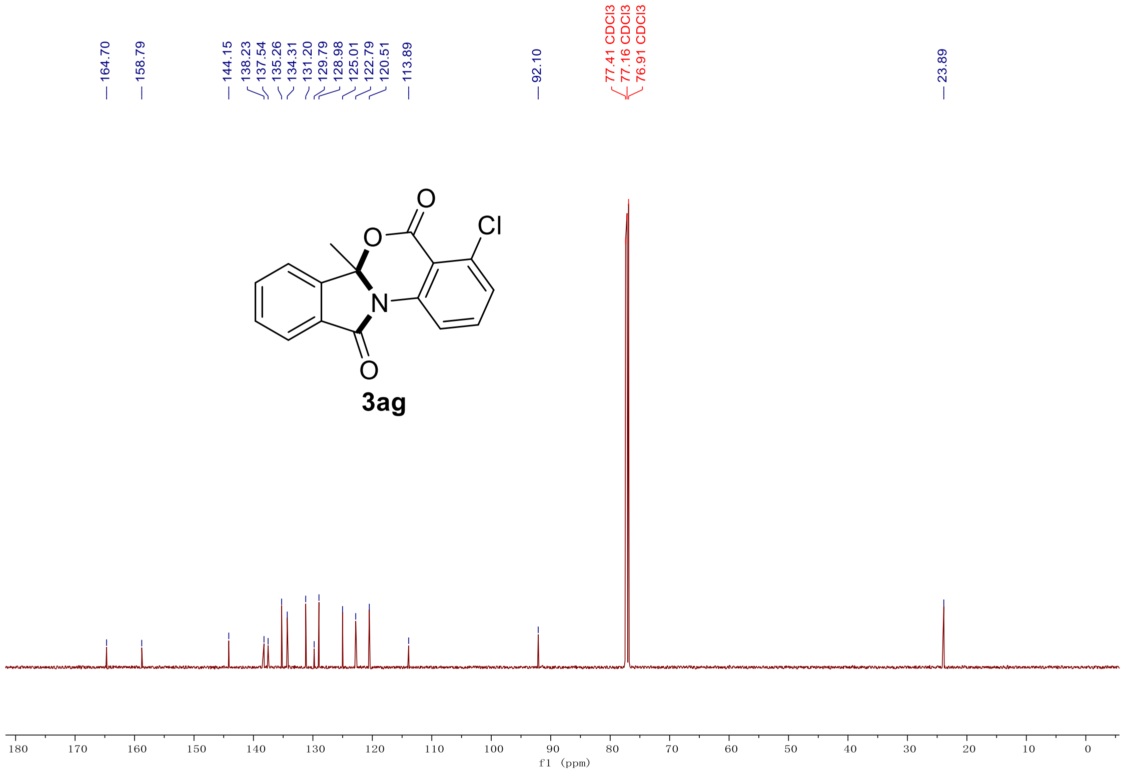

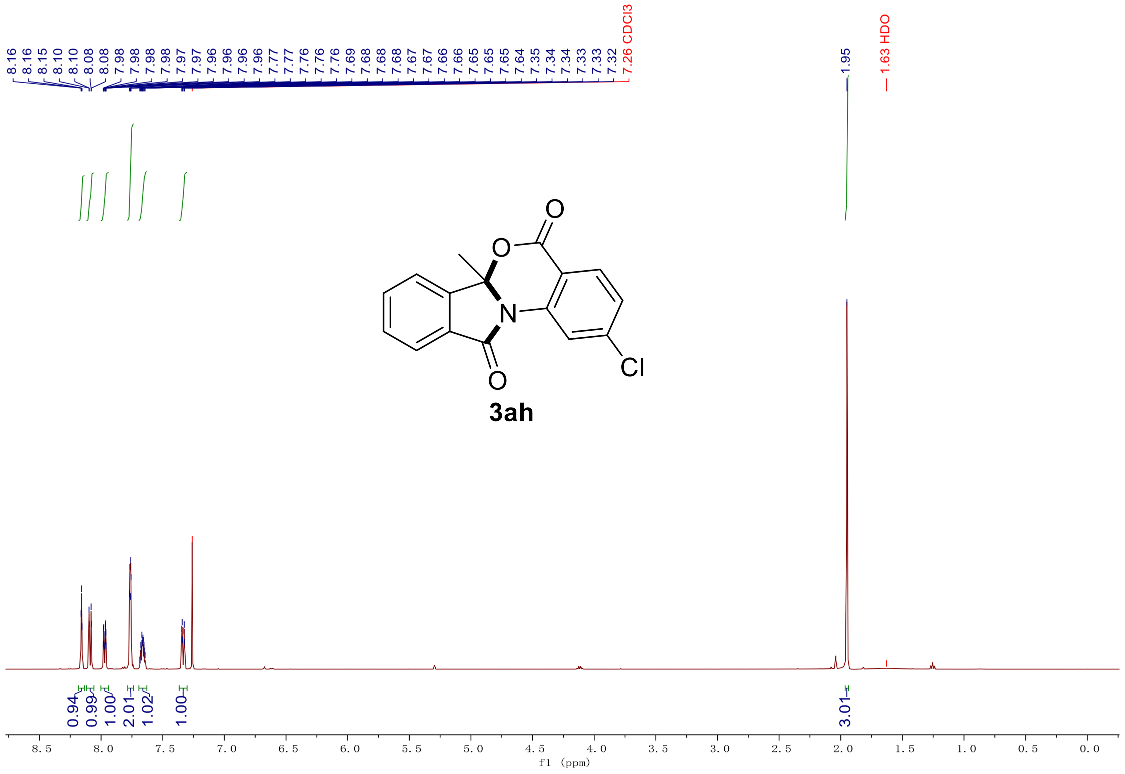
8.16 8.16 8.15 8.10 8.10 8.08 8.08 7.98 7.98 7.98 7.98 7.97 7.97 7.96 7.96 7.96 7.96 7.77 7.77 7.76 7.76 7.76 7.69 7.68 7.68 7.68 7.67 7.67 7.66 7.66 7.65 7.65 7.65 7.64 7.35 7.34 7.34 7.33 7.33 7.32
7.26 CDCl3
1.95
1.63 HDO
O
O
N
O
Cl
3ah
0.94 0.99 1.00 2.01 1.02 1.00
3.01
f1 (ppm)

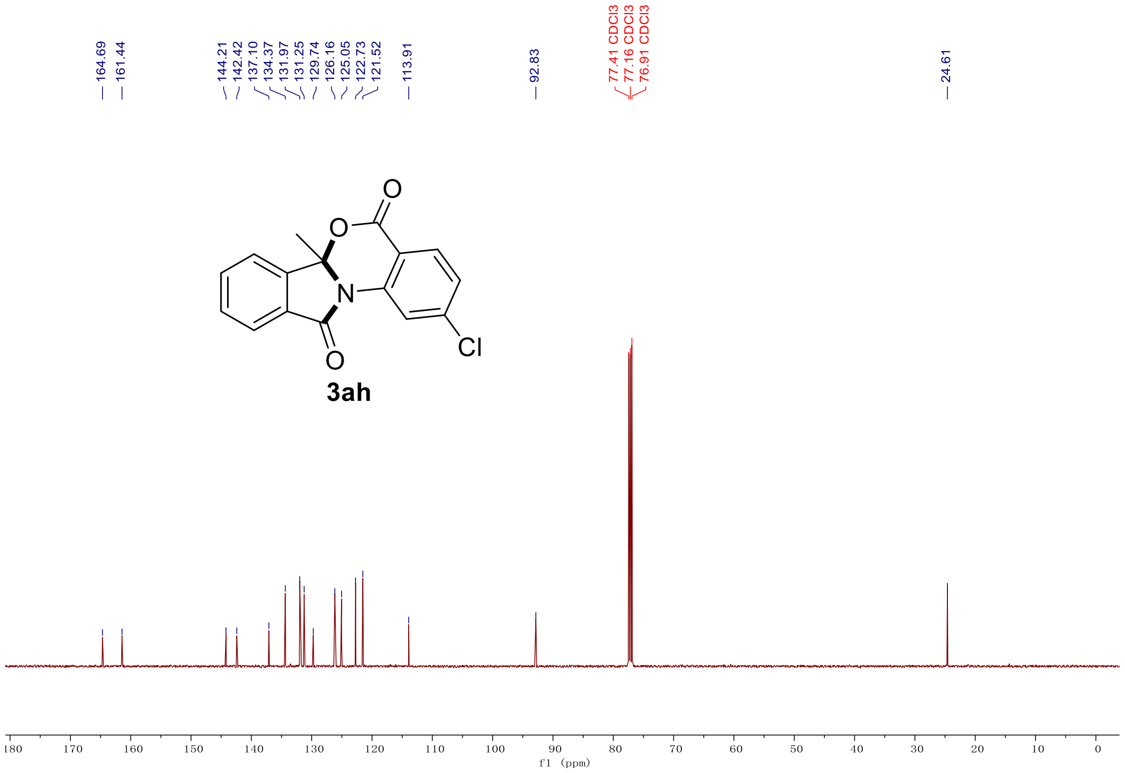
164.69
161.44
144.21 142.42 137.10 134.37 131.97 131.25 129.74 126.16 125.05 122.73 121.52
113.91
92.83
77.41 CDCl3
77.16 CDCl3
76.91 CDCl3
24.61
O
O
N
O
Cl
3ah
f1 (ppm)

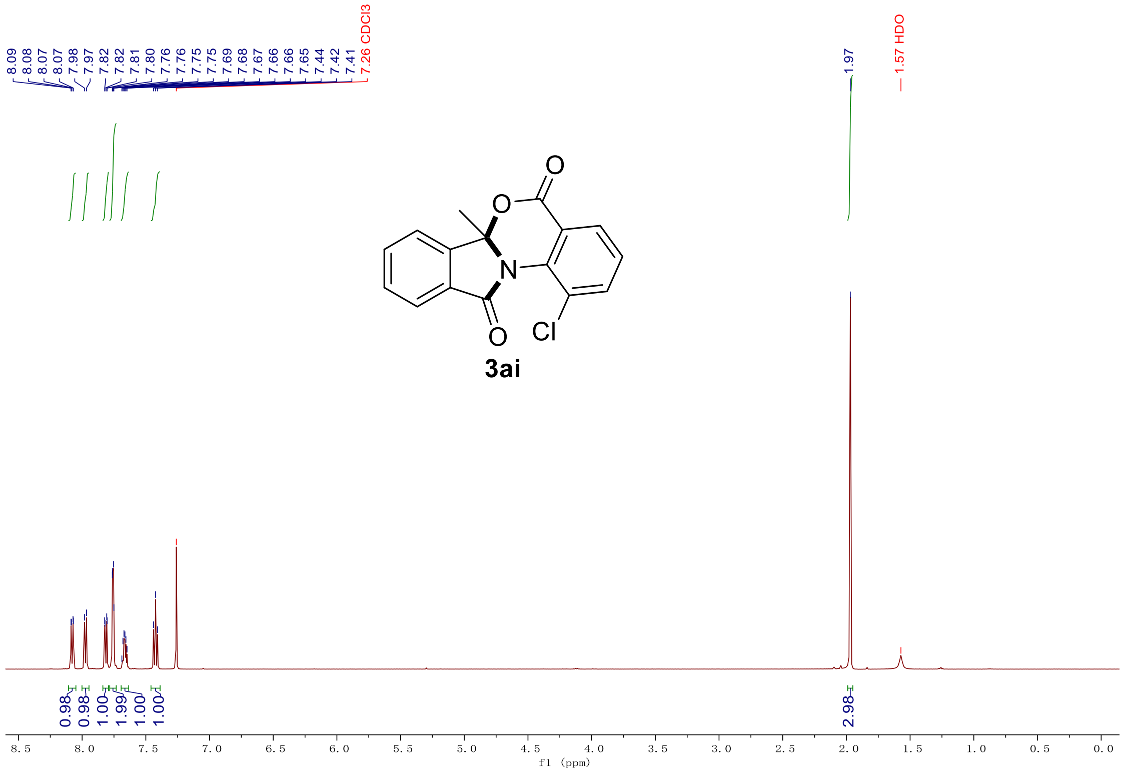
8.09
8.08
8.07
8.07
7.98
7.97
7.82
7.82
7.81
7.80
7.76
7.76
7.75
7.69
7.68
7.67
7.66
7.66
7.65
7.44
7.42
7.41
7.26 CDCl3
1.97
1.57 HDO
0.98
0.98
1.00
1.99
1.00
1.00
2.98
Cl
3ai
f1 (ppm)

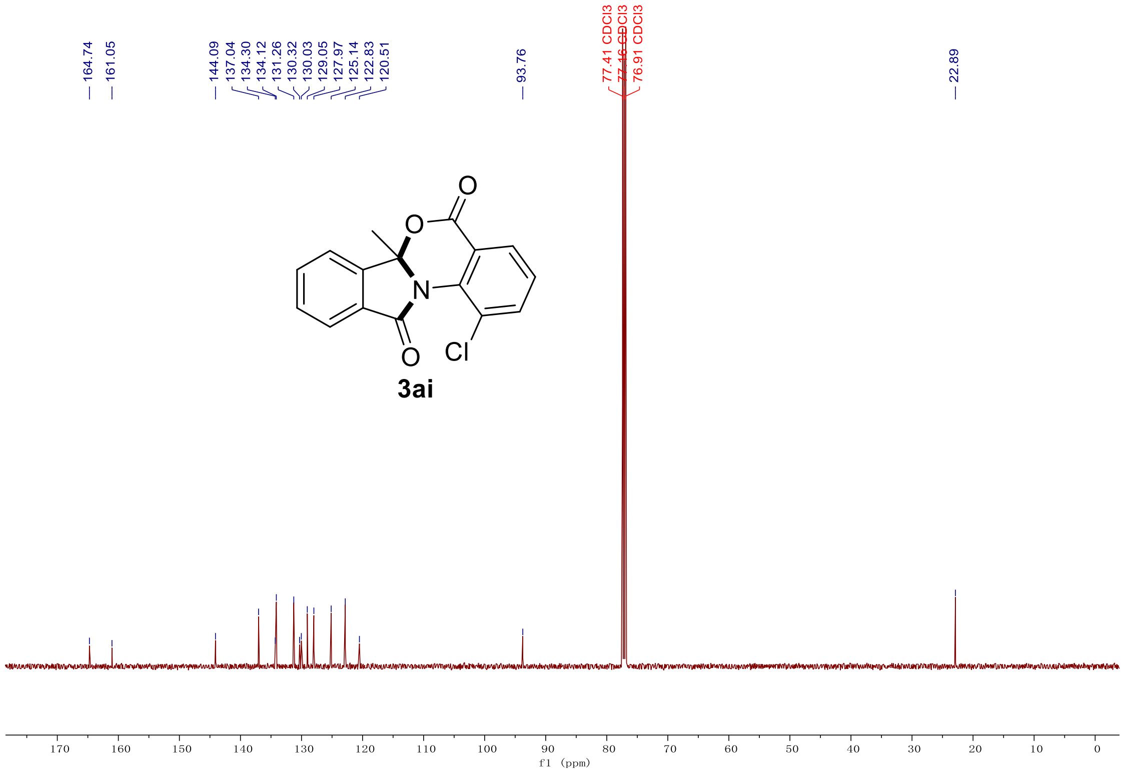
164.74
161.05
144.09
137.04
134.30
134.12
131.26
130.32
130.03
129.05
127.97
125.14
122.83
120.51
93.76
77.41 CDCl3
77.16 CDCl3
76.91 CDCl3
22.89
Cl
3ai
f1 (ppm)

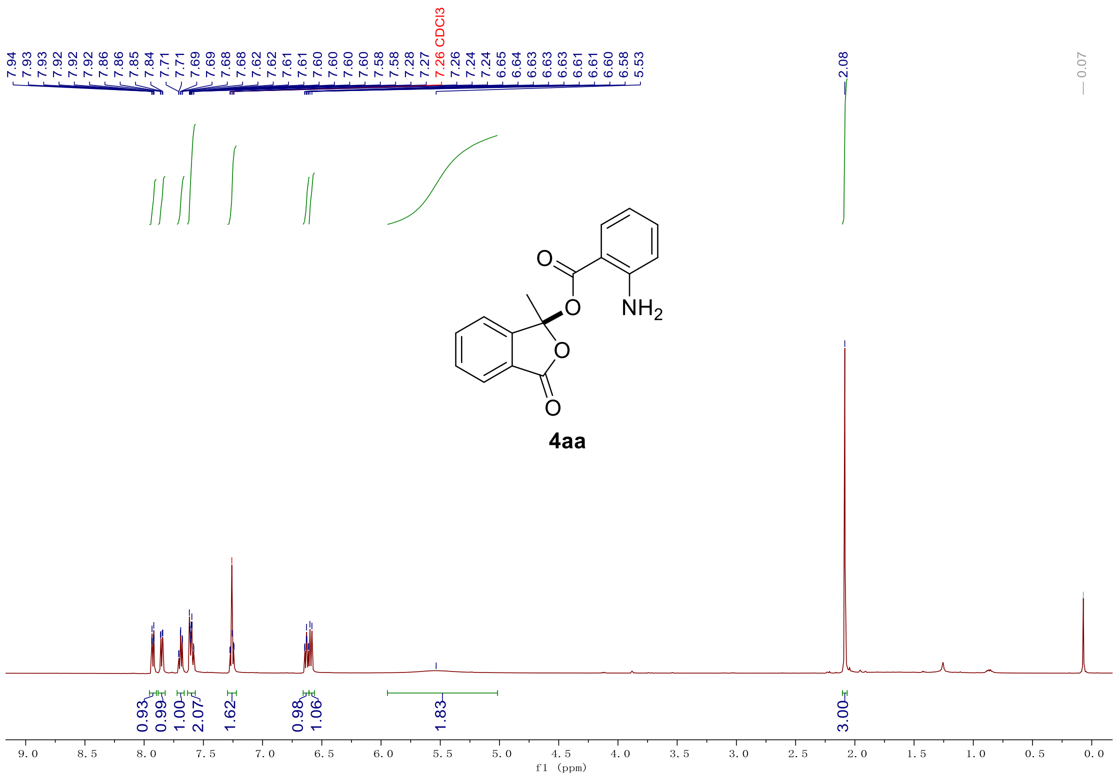
4aa

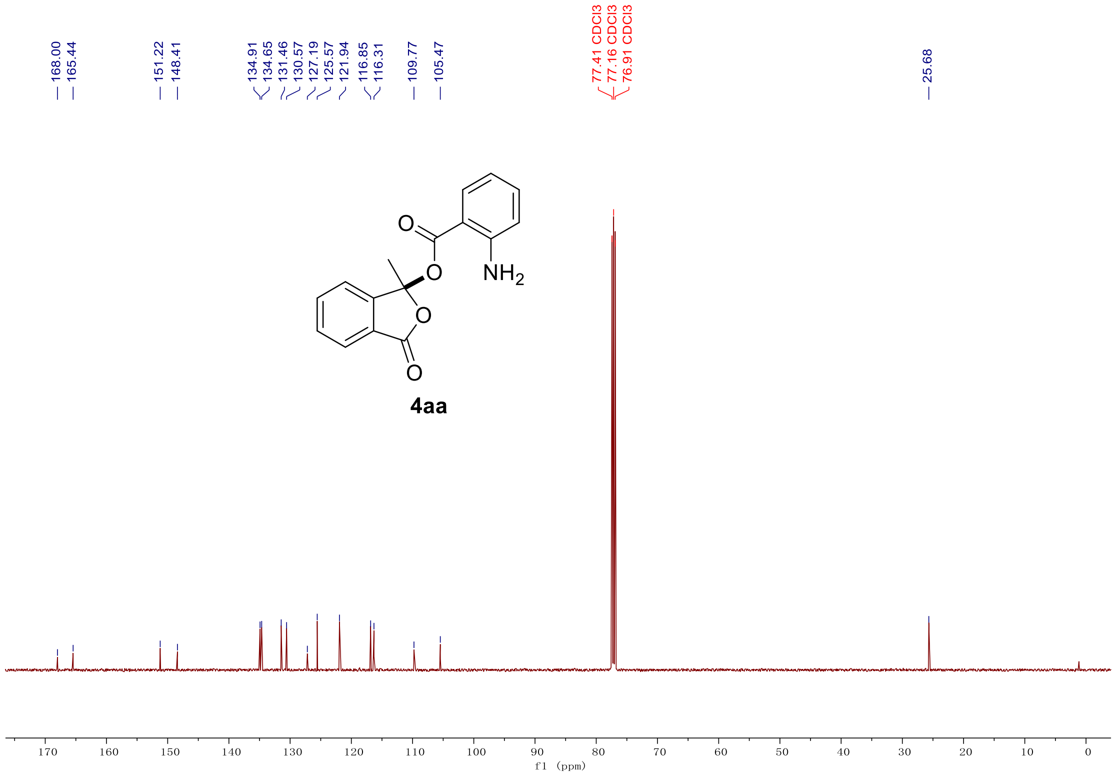
4aa

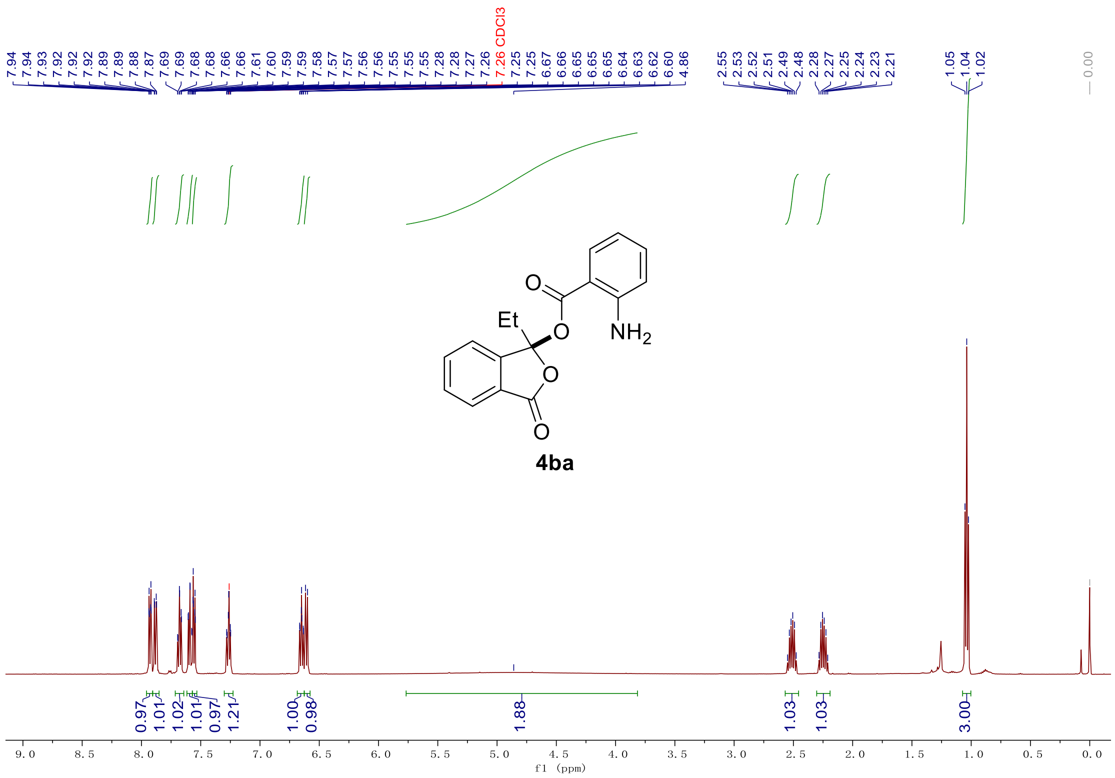
7.94 7.94 7.93 7.92 7.92 7.92 7.89 7.89 7.88 7.87 7.69 7.69 7.68 7.68 7.66 7.66 7.61 7.60 7.59 7.59 7.58 7.57 7.57 7.56 7.56 7.55 7.55 7.55 7.28 7.28 7.27 7.26
7.26 CDCl3
7.25 7.25 6.67 6.66 6.65 6.65 6.64 6.63 6.62 6.60 4.86
2.55 2.53 2.52 2.51 2.49 2.48 2.28 2.27 2.25 2.24 2.23 2.21
1.05 1.04 1.02
0.00
Et
O
NH2
4ba
0.97 1.01 1.02 1.01 0.97 1.21 1.00 0.98 1.88 1.03 1.03 3.00
9.0 8.5 8.0 7.5 7.0 6.5 6.0 5.5 5.0 4.5 4.0 3.5 3.0 2.5 2.0 1.5 1.0 0.5 0.0
f1 (ppm)

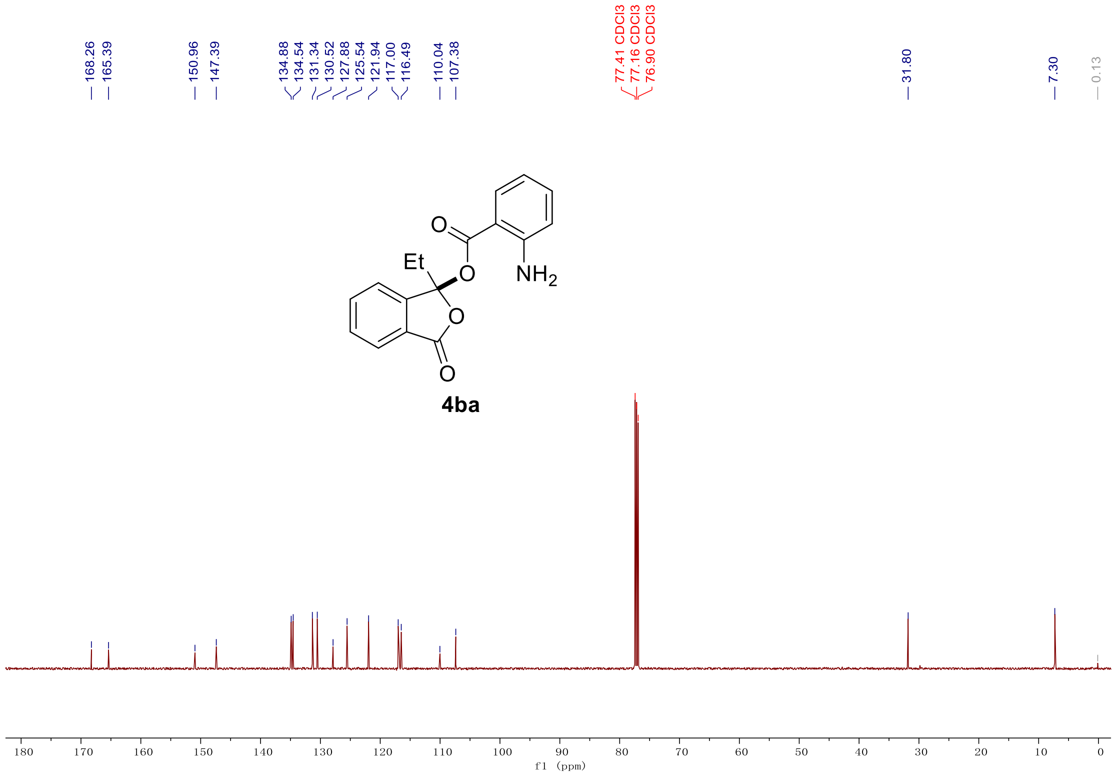
168.26 165.39 150.96 147.39
134.88 134.54 131.34 130.52 127.88 125.54 121.94 117.00 116.49
110.04 107.38
77.41 CDCl3
77.16 CDCl3
76.90 CDCl3
31.80 7.30 0.13
Et
O
NH2
4ba
180 170 160 150 140 130 120 110 100 90 80 70 60 50 40 30 20 10 0
f1 (ppm)

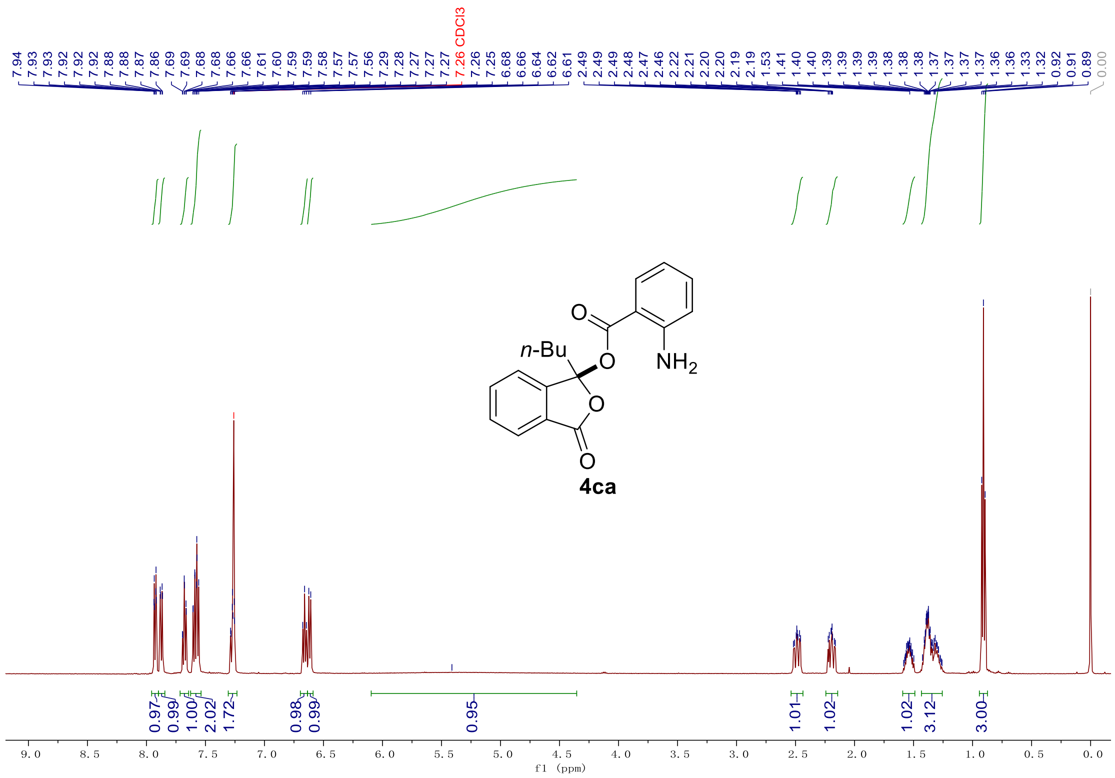

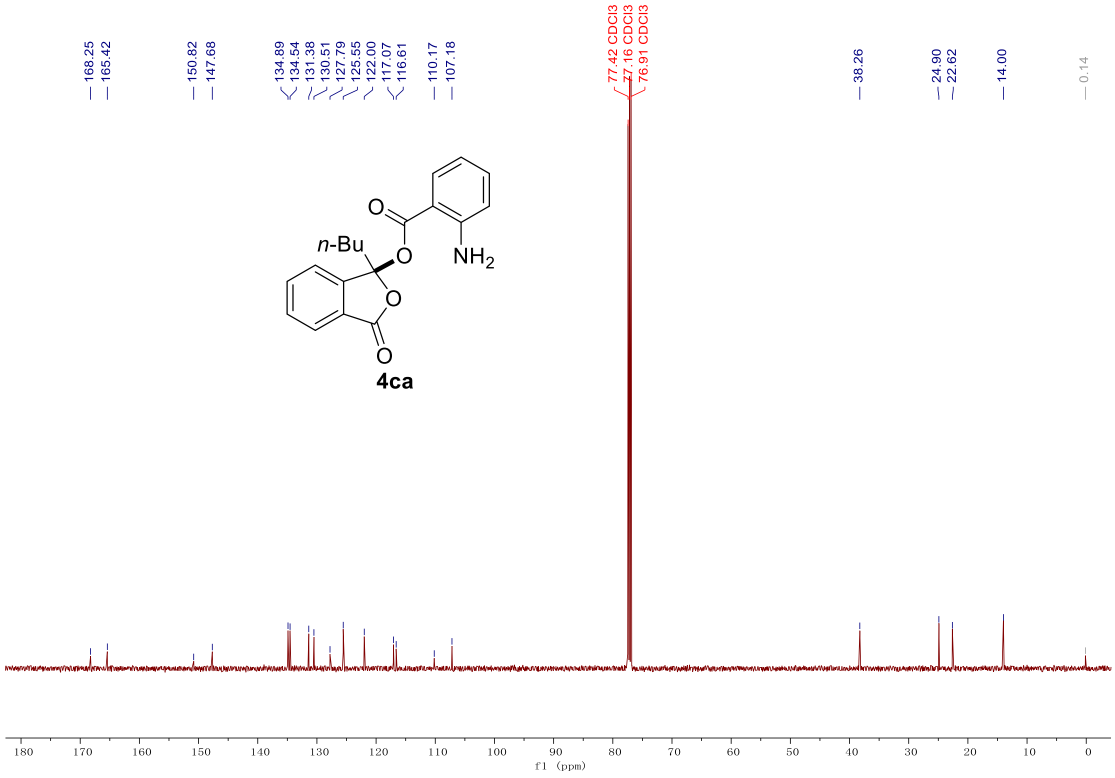

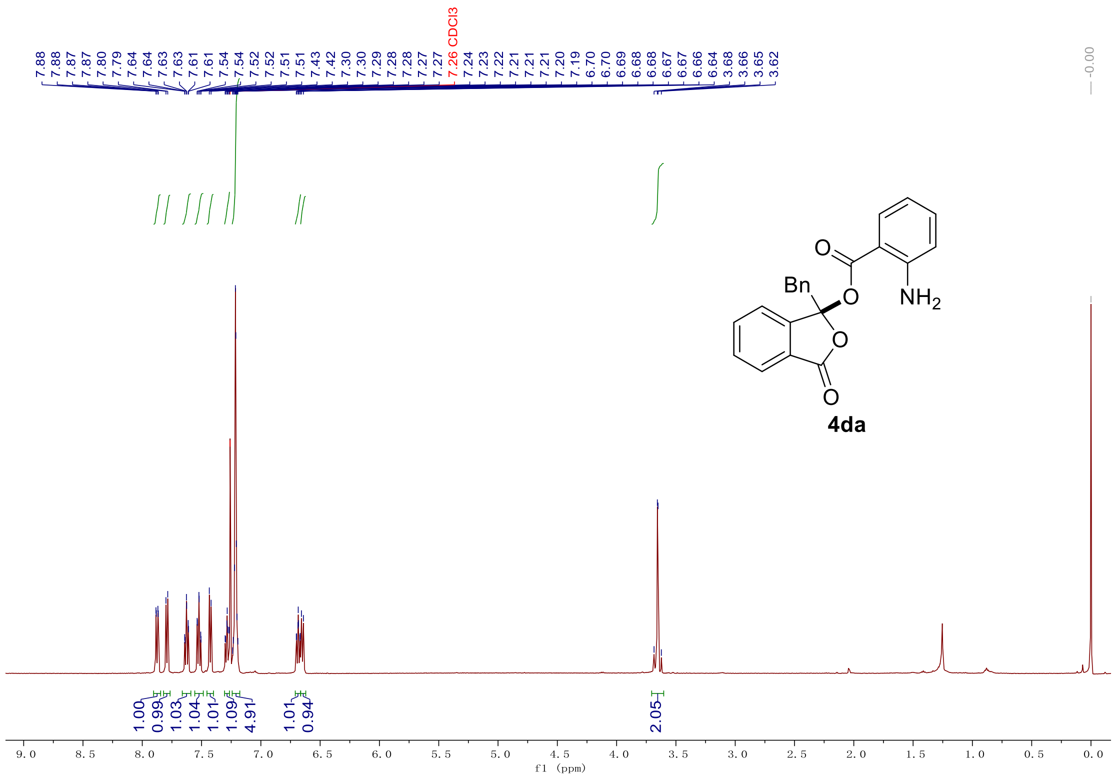

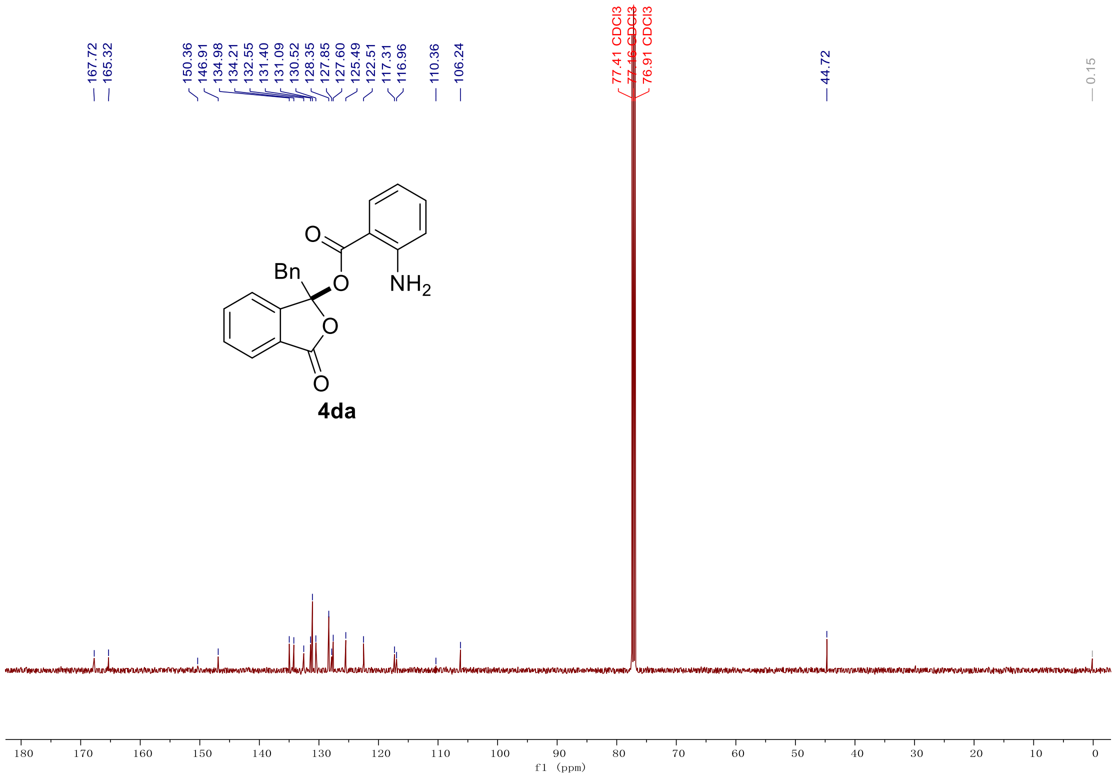

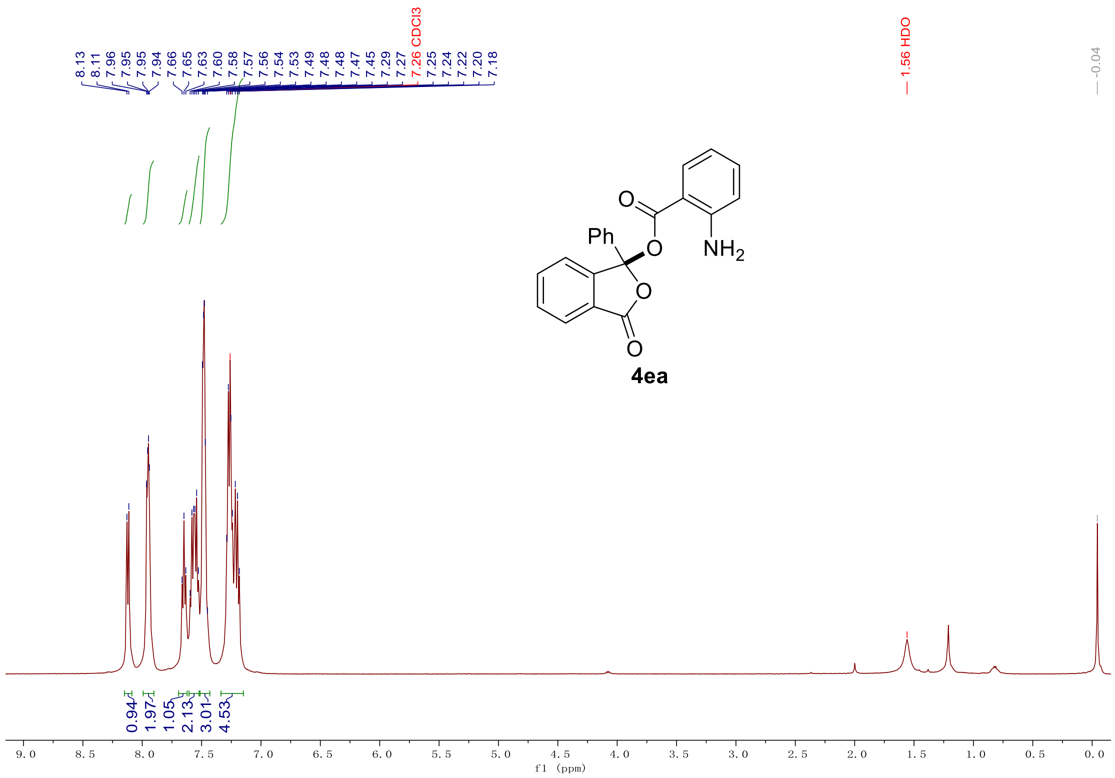
8.13 8.11 7.96 7.95 7.95 7.94 7.66 7.65 7.63 7.60 7.58 7.57 7.56 7.54 7.53 7.49 7.48 7.48 7.47 7.45 7.29 7.27
7.26 CDCl3
7.25 7.24 7.22 7.20 7.18
1.56 HDO
-0.04
Ph
O
NH2
4ea
0.94 1.97 1.05 2.13 3.01 4.53
9.0 8.5 8.0 7.5 7.0 6.5 6.0 5.5 5.0 4.5 4.0 3.5 3.0 2.5 2.0 1.5 1.0 0.5 0.0
f1 (ppm)

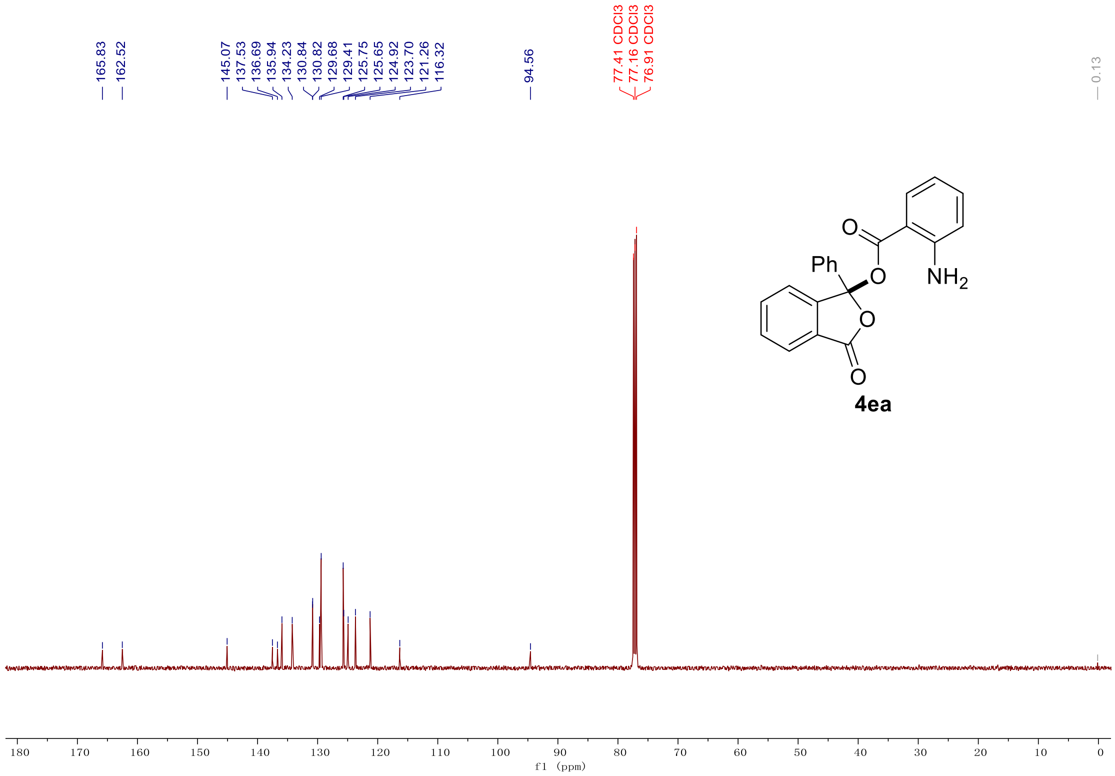
165.83 162.52
145.07 137.53 136.69 135.94 134.23 130.84 130.82 129.68 129.41 125.75 125.65 124.92 123.70 121.26 116.32
94.56
77.41 CDCl3
77.16 CDCl3
76.91 CDCl3
0.13
Ph
O
NH2
4ea
180 170 160 150 140 130 120 110 100 90 80 70 60 50 40 30 20 10 0
f1 (ppm)

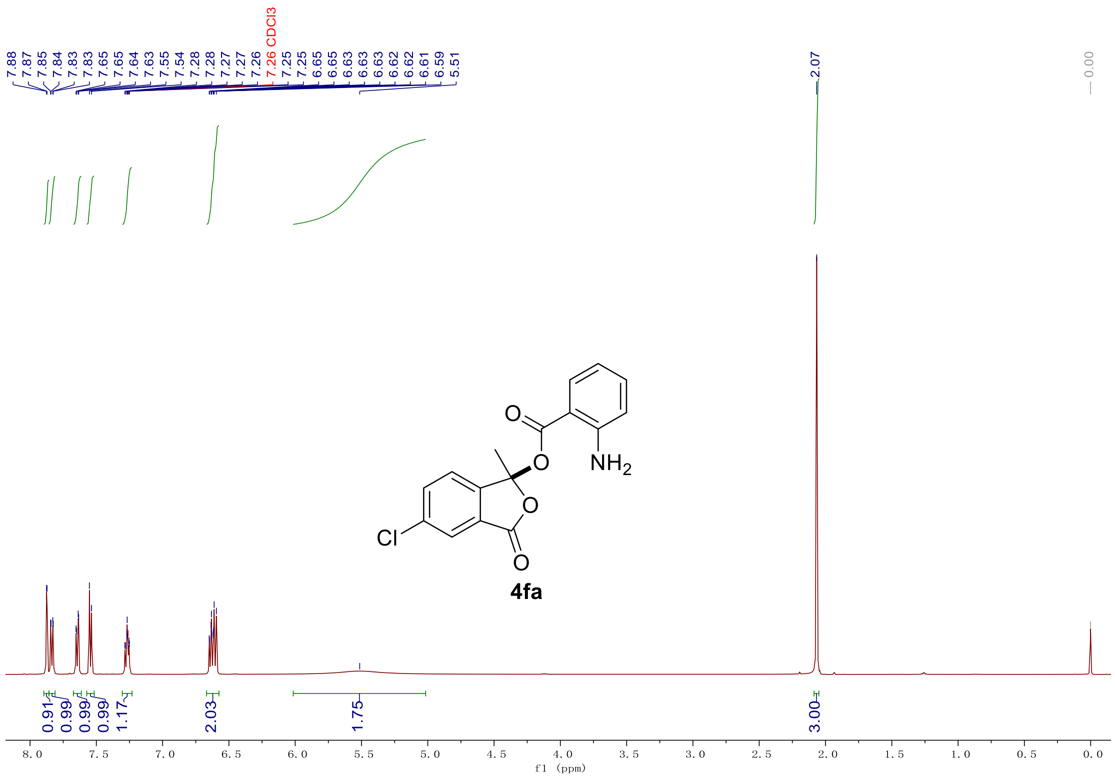
7.88 7.87 7.85 7.84 7.83 7.83 7.65 7.65 7.64 7.63 7.55 7.54 7.28 7.28 7.27 7.26 7.26 CDCl3 7.25 7.25 6.65 6.65 6.63 6.63 6.62 6.62 6.61 6.59 5.51
2.07
0.00
O
O
NH2
O
Cl
O
4fa
0.91 0.99 0.99 0.99 1.17 2.03 1.75 3.00
8.0 7.5 7.0 6.5 6.0 5.5 5.0 4.5 4.0 3.5 3.0 2.5 2.0 1.5 1.0 0.5 0.0
f1 (ppm)

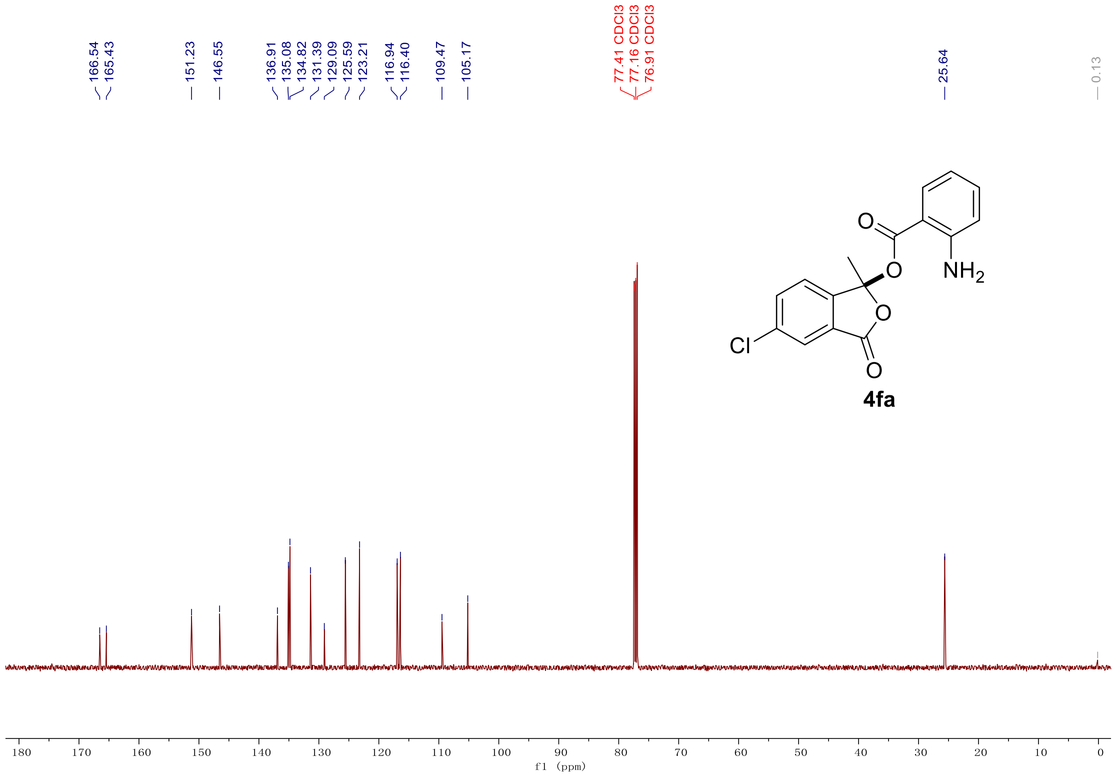
166.54 165.43
151.23
146.55
136.91 135.08 134.82 131.39 129.09 125.59 123.21
116.94 116.40
109.47
105.17
77.41 CDCl3 77.16 CDCl3 76.91 CDCl3
25.64
0.13
O
O
NH2
O
Cl
O
4fa
180 170 160 150 140 130 120 110 100 90 80 70 60 50 40 30 20 10 0
f1 (ppm)

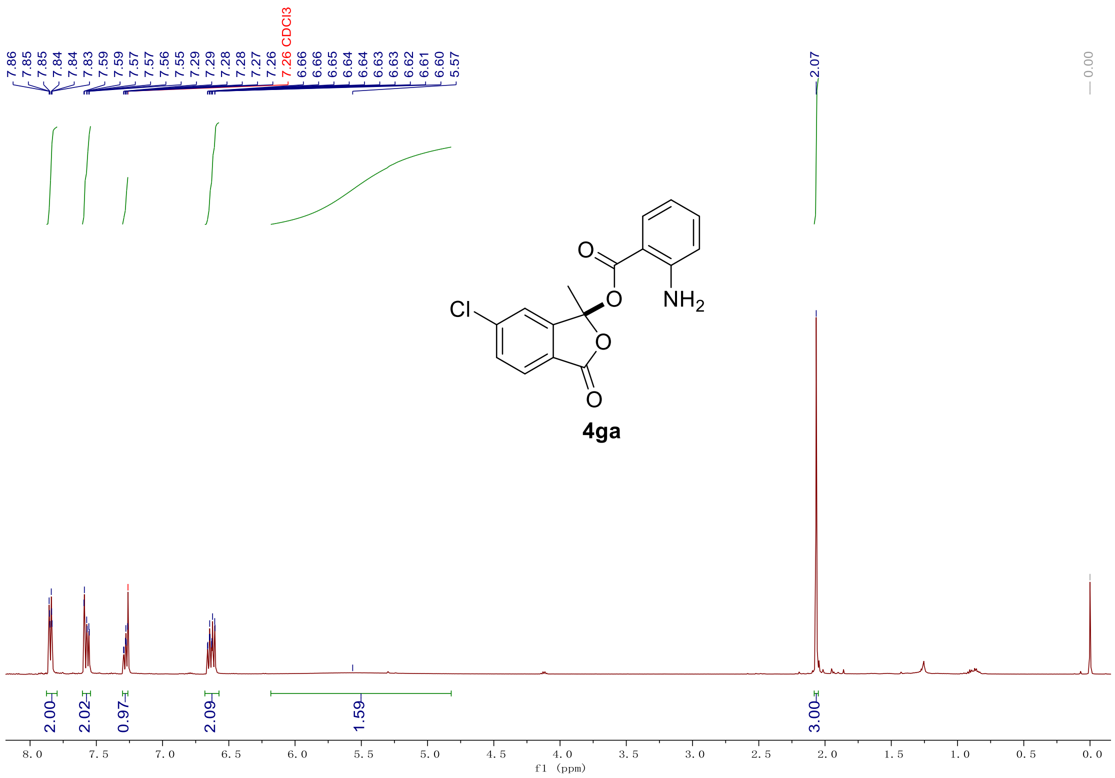

7.86
7.85
7.85
7.84
7.84
7.83
7.59
7.59
7.57
7.57
7.56
7.55
7.29
7.29
7.28
7.28
7.27
7.26
7.26 CDCl3
6.66
6.66
6.65
6.64
6.64
6.63
6.63
6.62
6.61
6.60
5.57
2.07
0.00
Cl
O
O
O
O
O
NH2
4ga
2.00
2.02
0.97
2.09
1.59
3.00
f1 (ppm)

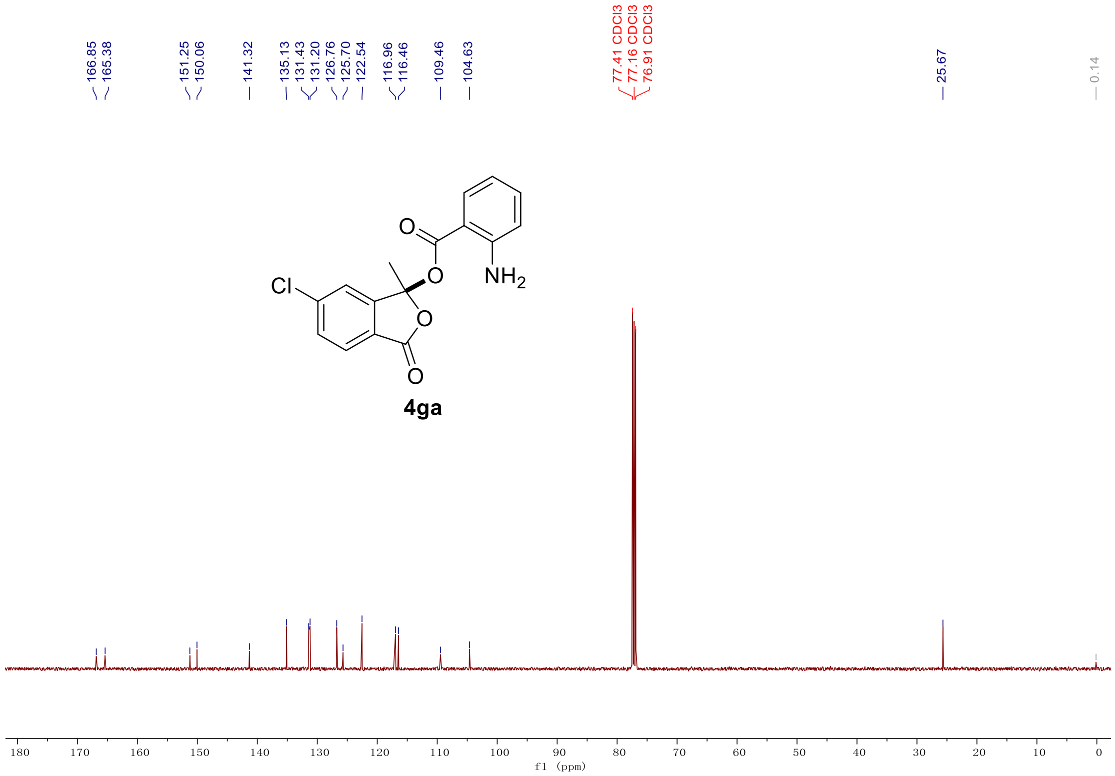

166.85
165.38
151.25
150.06
141.32
135.13
131.43
131.20
126.76
125.70
122.54
116.96
116.46
109.46
104.63
77.41 CDCl3
77.16 CDCl3
76.91 CDCl3
25.67
0.14
Cl
O
O
O
O
O
NH2
4ga
f1 (ppm)

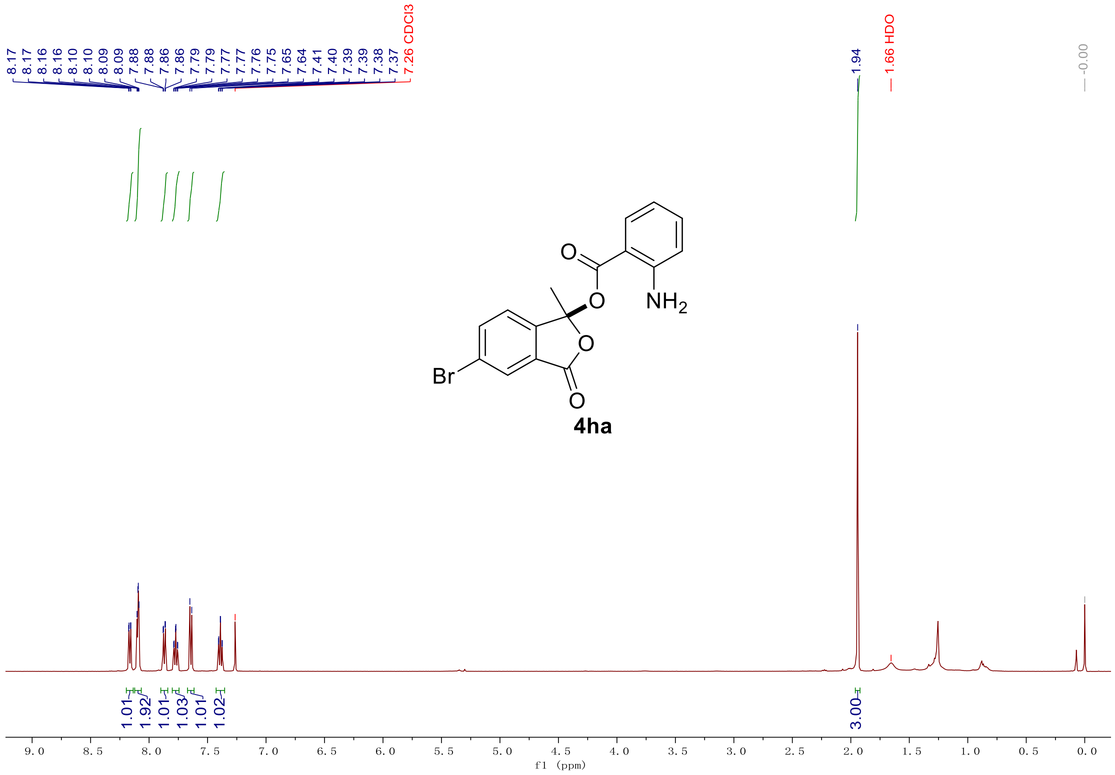
8.17 8.17 8.16 8.16 8.10 8.10 8.09 8.09 7.88 7.88 7.86 7.86 7.79 7.79 7.77 7.77 7.76 7.75 7.65 7.64 7.41 7.40 7.39 7.39 7.38 7.37
7.26 CDCl3
1.94
1.66 HDO
-0.00
O
O
NH2
O
Br
O
O
4ha
1.01
1.92
1.01
1.03
1.01
1.02
3.00
f1 (ppm)

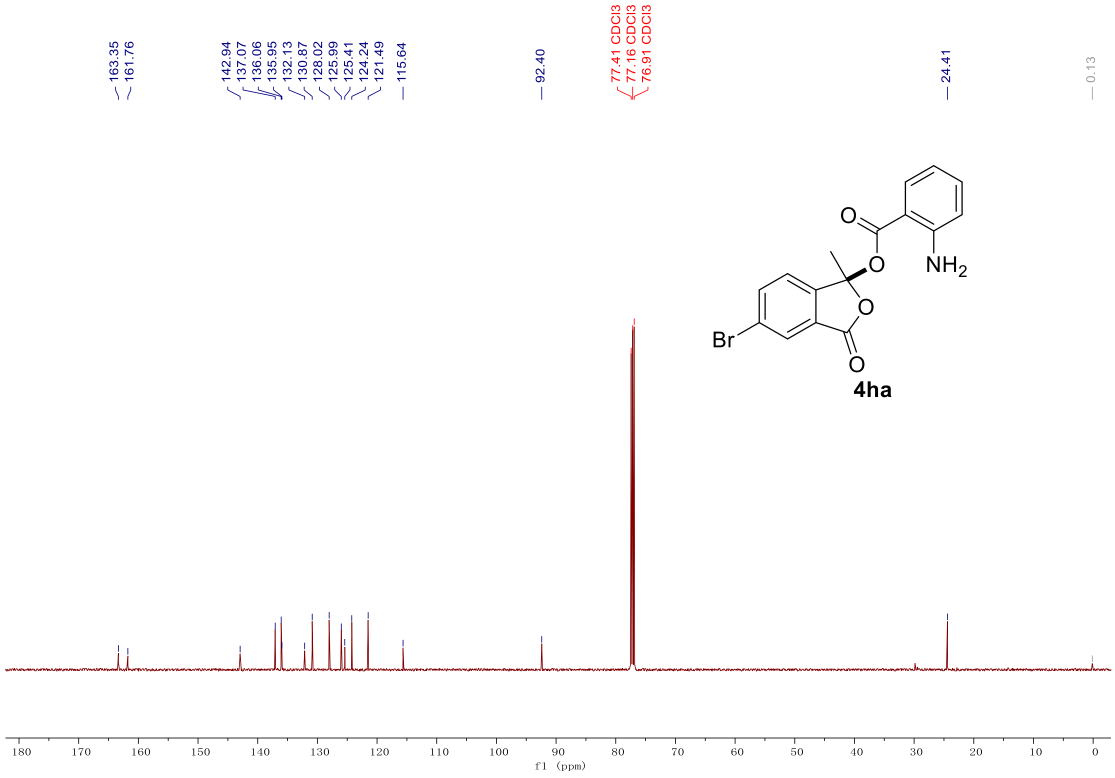
163.35 161.76
142.94 137.07 136.06 135.95 132.13 130.87 128.02 125.99 125.41 124.24 121.49
115.64
92.40
77.41 CDCl3 77.16 CDCl3 76.91 CDCl3
24.41
0.13
O
O
NH2
O
Br
O
O
4ha
f1 (ppm)

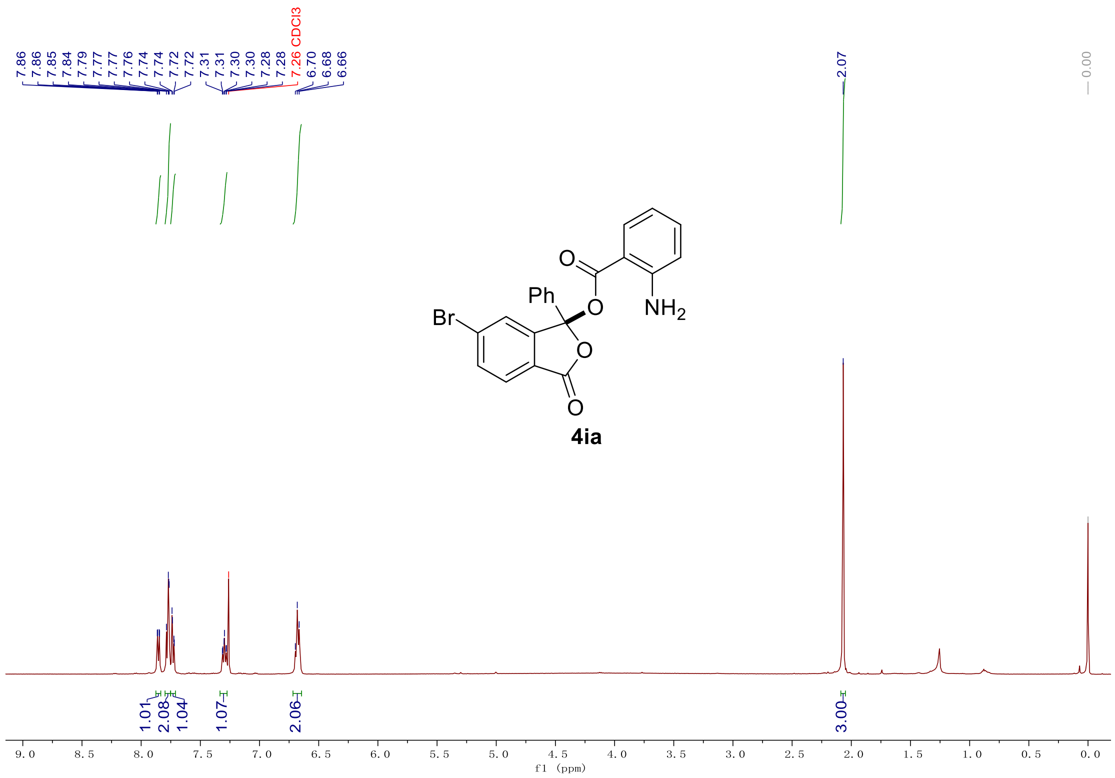
7.86
7.86
7.85
7.84
7.79
7.77
7.77
7.76
7.74
7.74
7.72
7.72
7.31
7.31
7.30
7.30
7.28
7.28
7.26 CDCl3
6.70
6.68
6.66
2.07
0.00
Br
Ph
O
O
NH2
O
O
4ia
1.01
2.08
1.04
1.07
2.06
3.00
f1 (ppm)

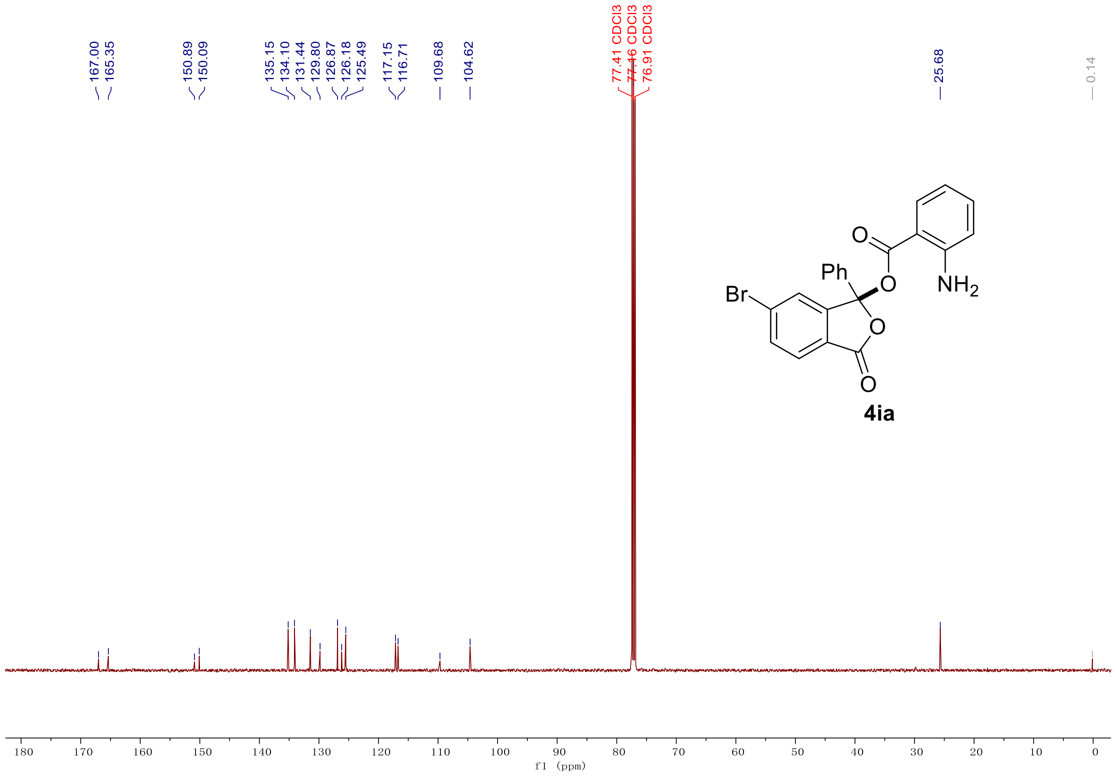
167.00
165.35
150.89
150.09
135.15
134.10
131.44
129.80
126.87
126.18
125.49
117.15
116.71
109.68
104.62
77.41 CDCl3
77.16 CDCl3
76.91 CDCl3
25.68
0.14
Br
Ph
O
O
NH2
O
O
4ia
f1 (ppm)

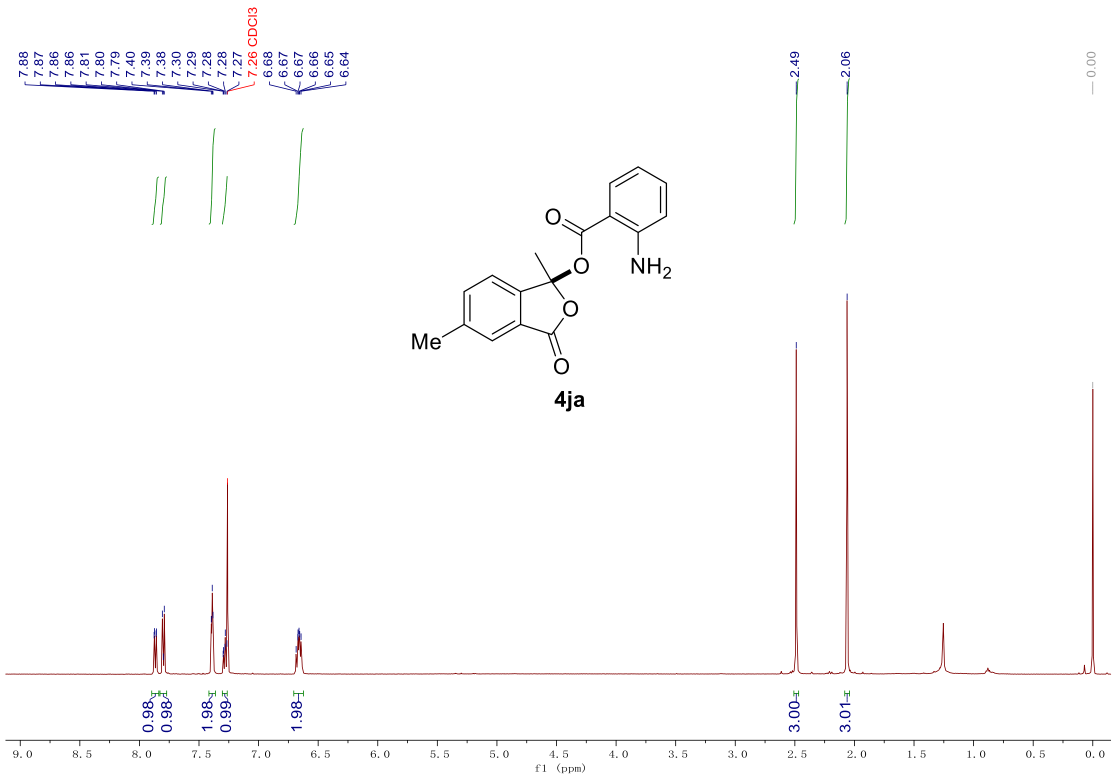

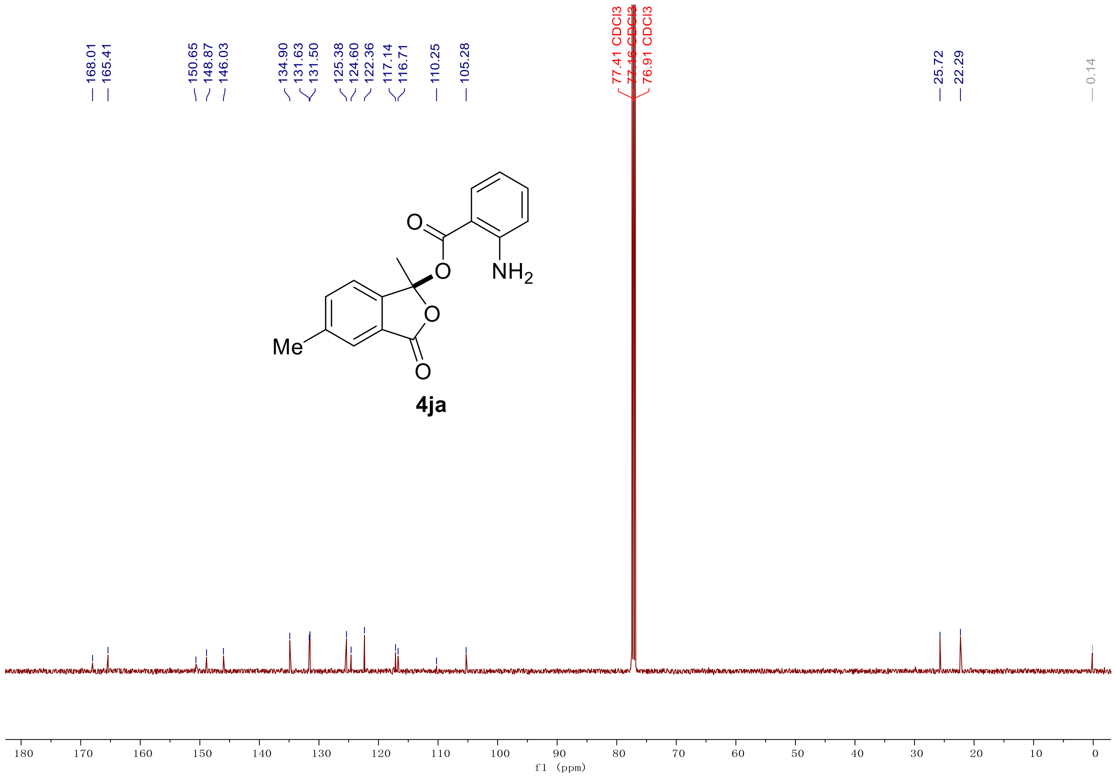

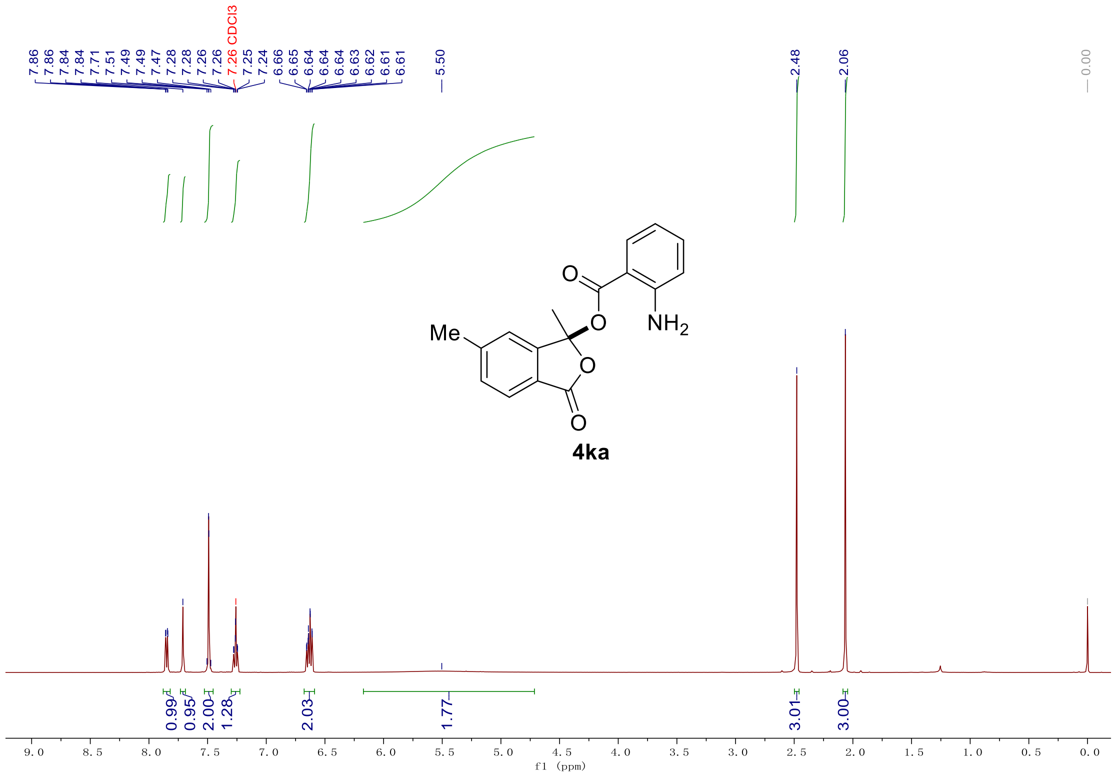
7.86
7.86
7.84
7.84
7.71
7.51
7.49
7.49
7.47
7.28
7.28
7.26
7.26
7.26 CDCl3
7.25
7.24
6.66
6.65
6.64
6.64
6.64
6.63
6.62
6.61
6.61
5.50
2.48
2.06
0.00
Me
O
O
O
O
NH2
4ka
0.99
0.95
2.00
1.28
2.03
1.77
3.01
3.00
f1 (ppm)

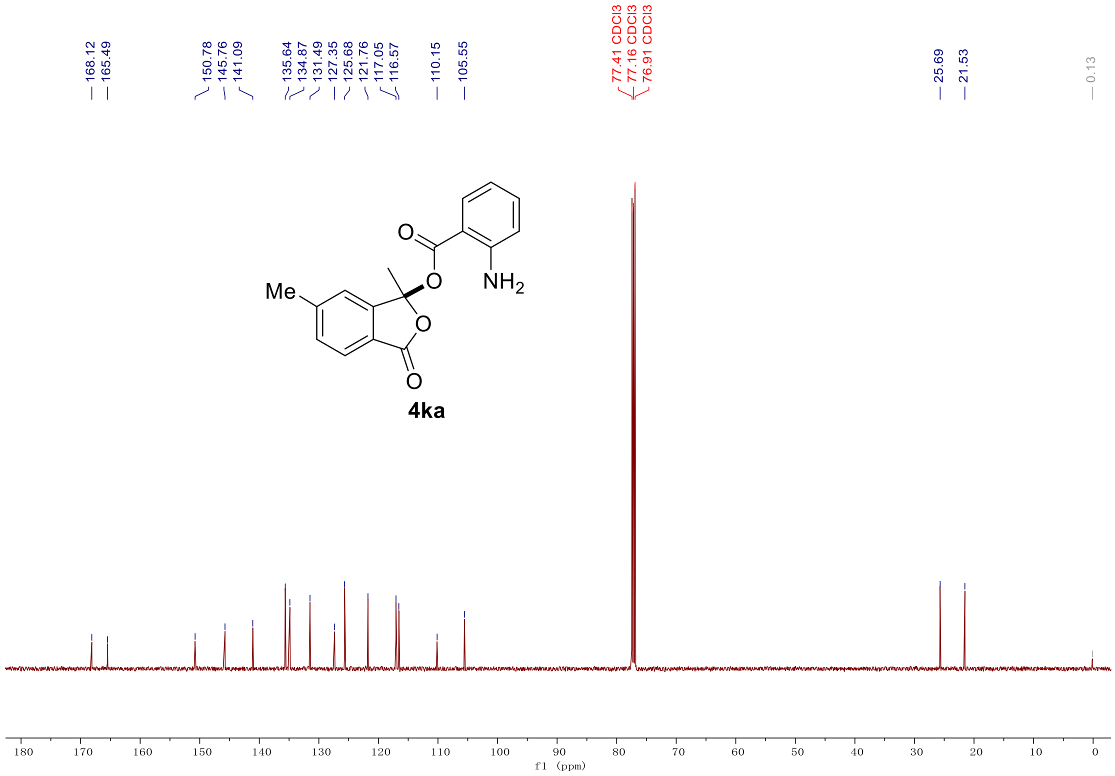
168.12
165.49
150.78
145.76
141.09
135.64
134.87
131.49
127.35
125.68
121.76
117.05
116.57
110.15
105.55
77.41 CDCl3
77.16 CDCl3
76.91 CDCl3
25.69
21.53
0.13
Me
O
O
O
O
NH2
4ka
f1 (ppm)

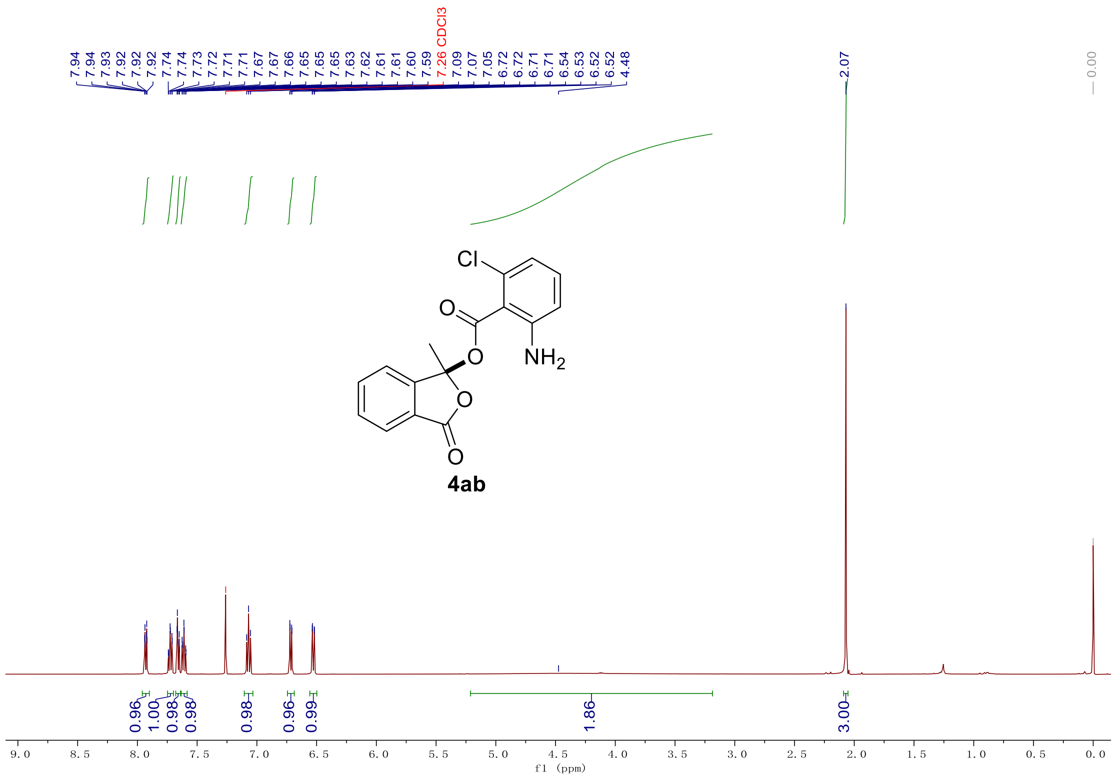

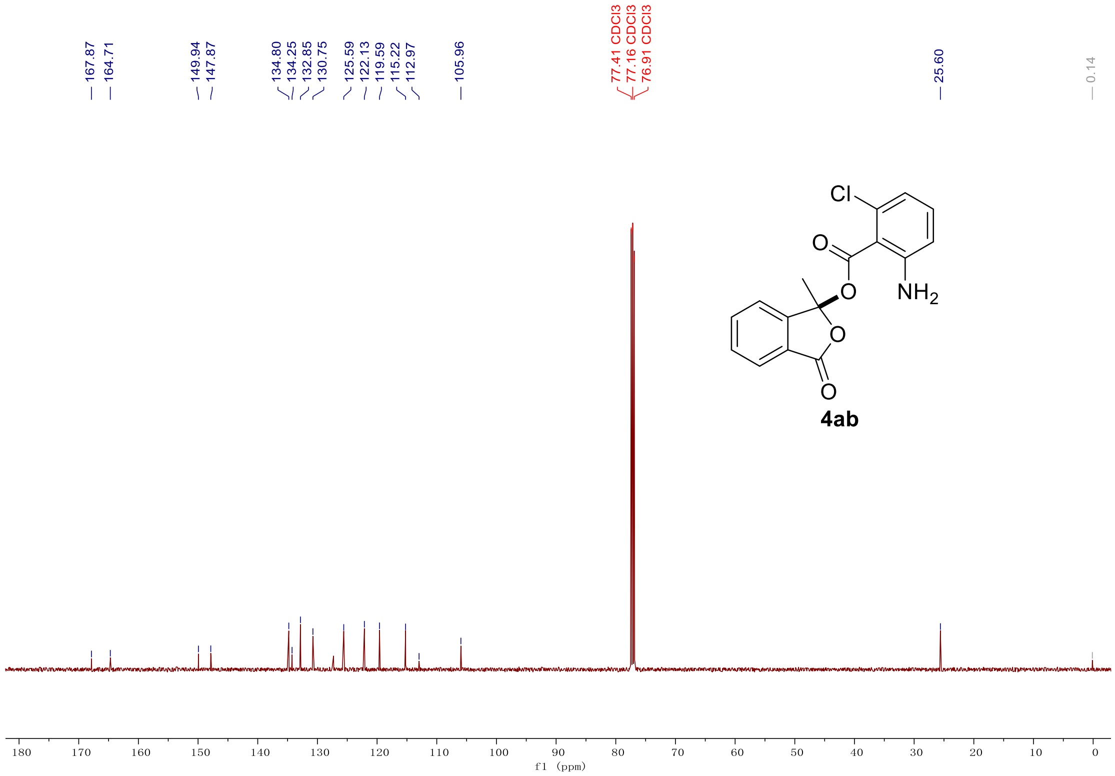

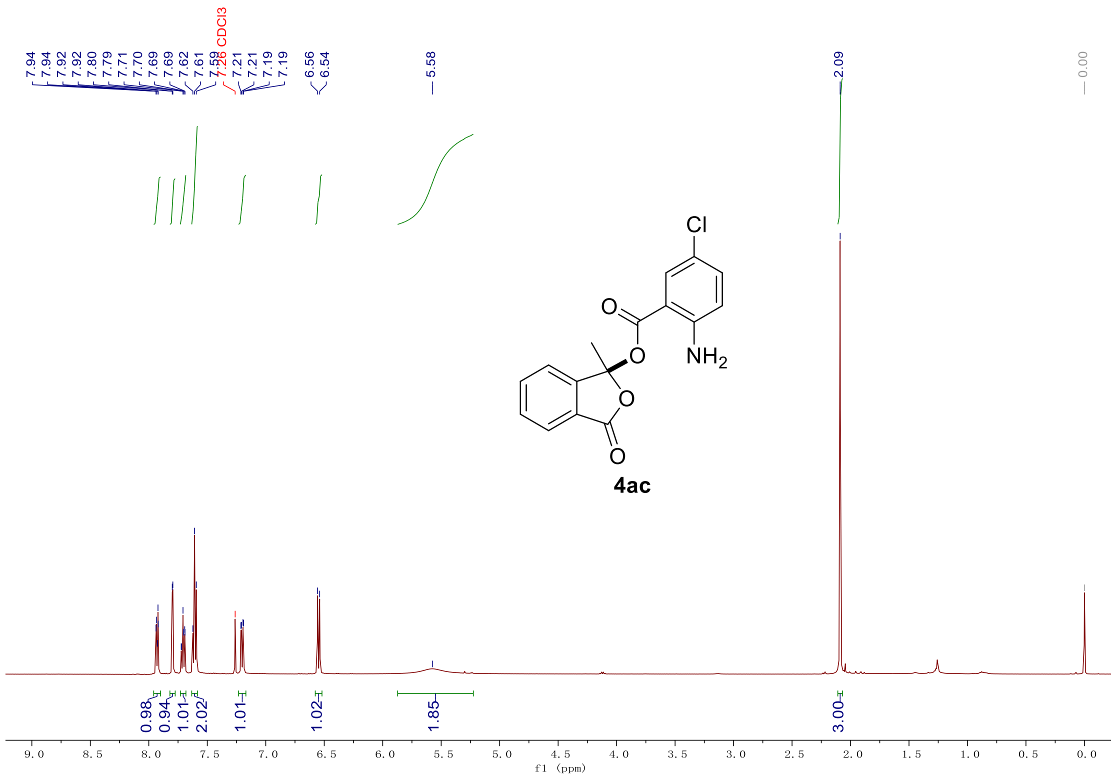
7.94
7.94
7.92
7.92
7.80
7.79
7.71
7.70
7.69
7.69
7.62
7.61
7.59
7.26 CDCl3
7.21
7.21
7.19
7.19
6.56
6.54
5.58
2.09
0.00
Cl
O
O
NH2
O
O
4ac
0.98
0.94
1.01
2.02
1.01
1.02
1.85
3.00
9.0
8.5
8.0
7.5
7.0
6.5
6.0
5.5
5.0
4.5
4.0
3.5
3.0
2.5
2.0
1.5
1.0
0.5
0.0
f1 (ppm)

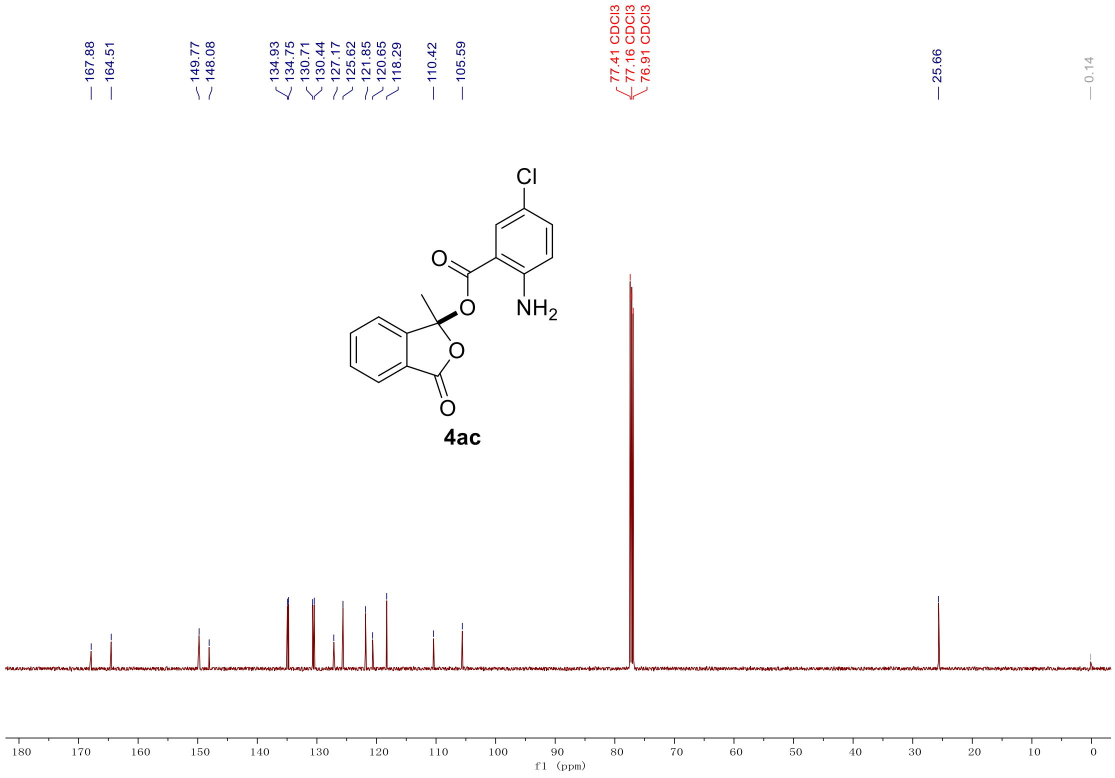
167.88
164.51
149.77
148.08
134.93
134.75
130.71
130.44
127.17
125.62
121.85
120.65
118.29
110.42
105.59
77.41 CDCl3
77.16 CDCl3
76.91 CDCl3
25.66
0.14
Cl
O
O
NH2
O
O
4ac
180
170
160
150
140
130
120
110
100
90
80
70
60
50
40
30
20
10
0
f1 (ppm)

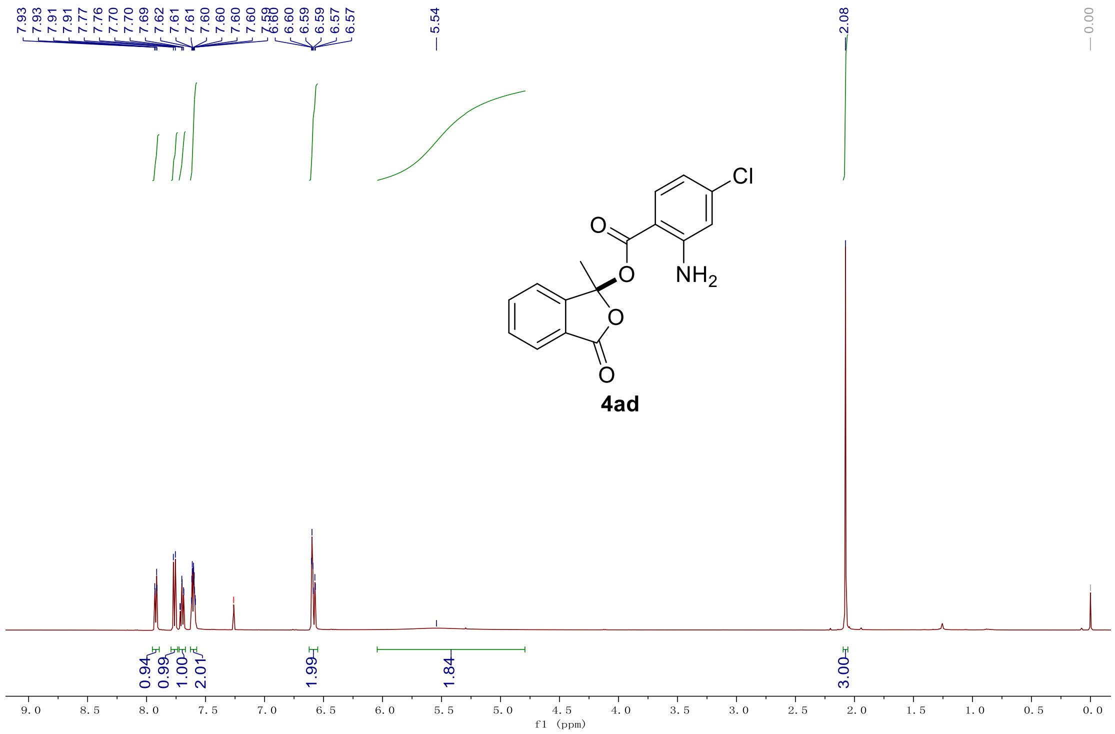
7.93
7.93
7.91
7.91
7.77
7.76
7.70
7.70
7.69
7.62
7.61
7.61
7.60
7.60
7.60
7.60
6.60
6.59
6.59
6.57
6.57
5.54
2.08
0.00
Cl
O
O
NH2
O
O
4ad
0.94
0.99
1.00
2.01
1.99
1.84
3.00
f1 (ppm)

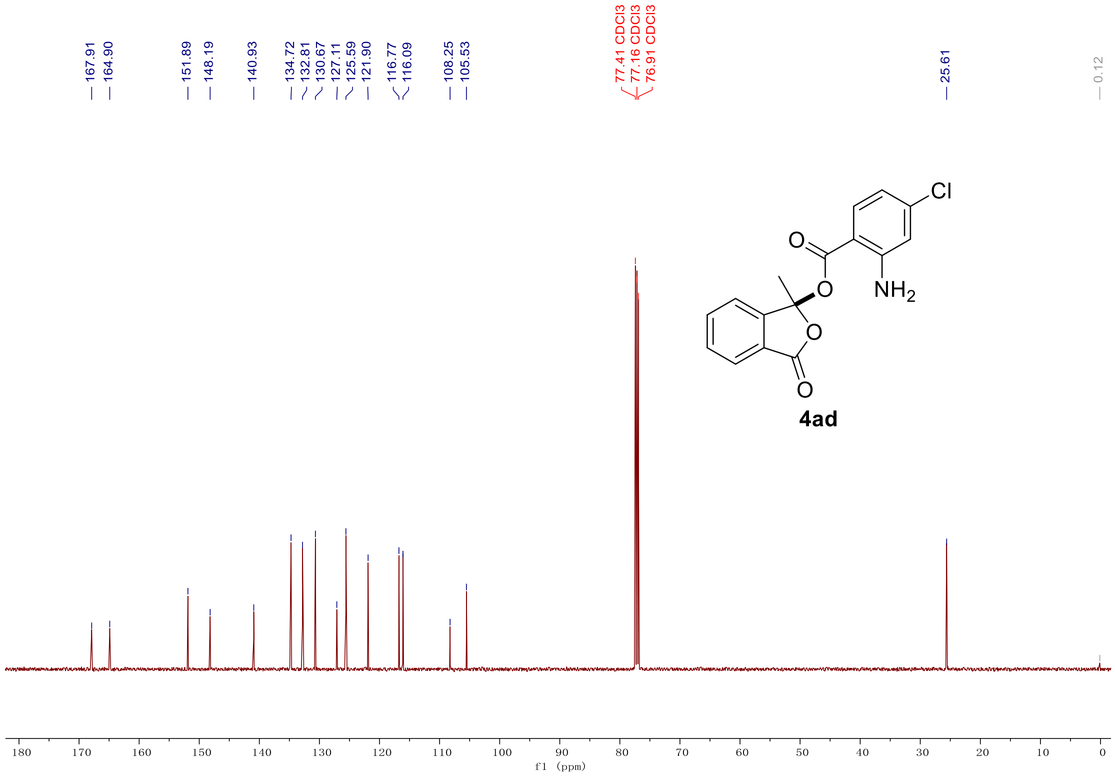
167.91
164.90
151.89
148.19
140.93
134.72
132.81
130.67
127.11
125.59
121.90
116.77
116.09
108.25
105.53
77.41 CDCl3
77.16 CDCl3
76.91 CDCl3
25.61
0.12
Cl
O
O
NH2
O
O
4ad
f1 (ppm)

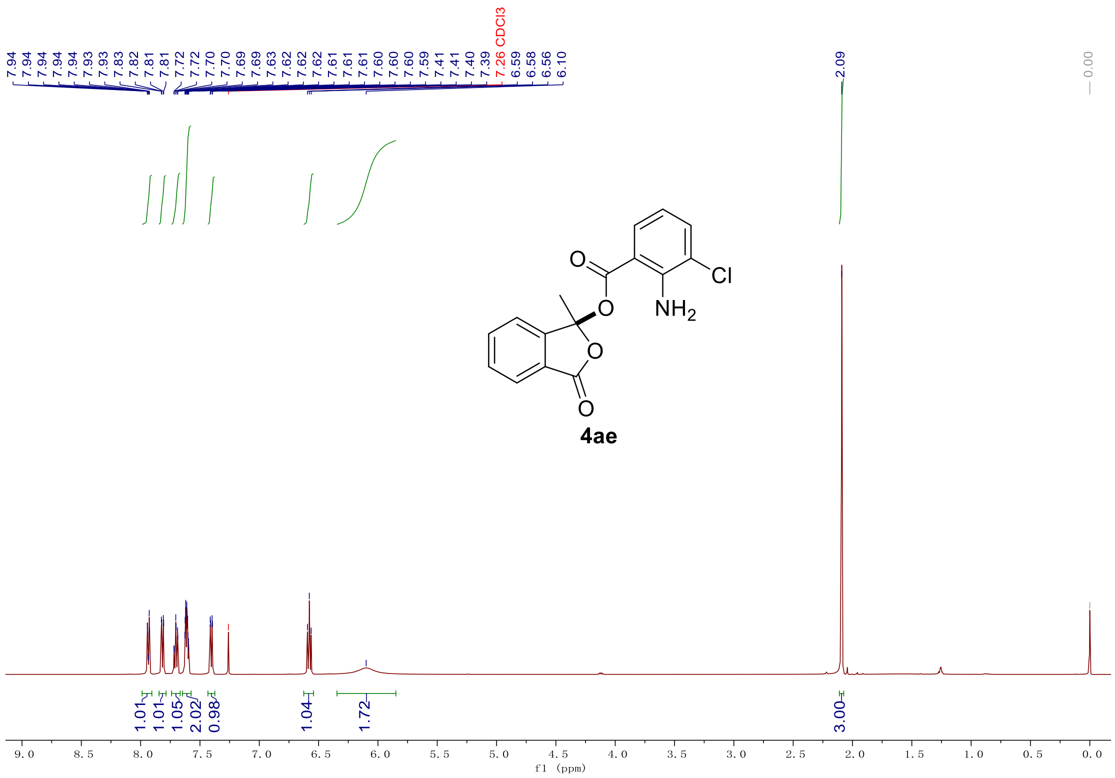

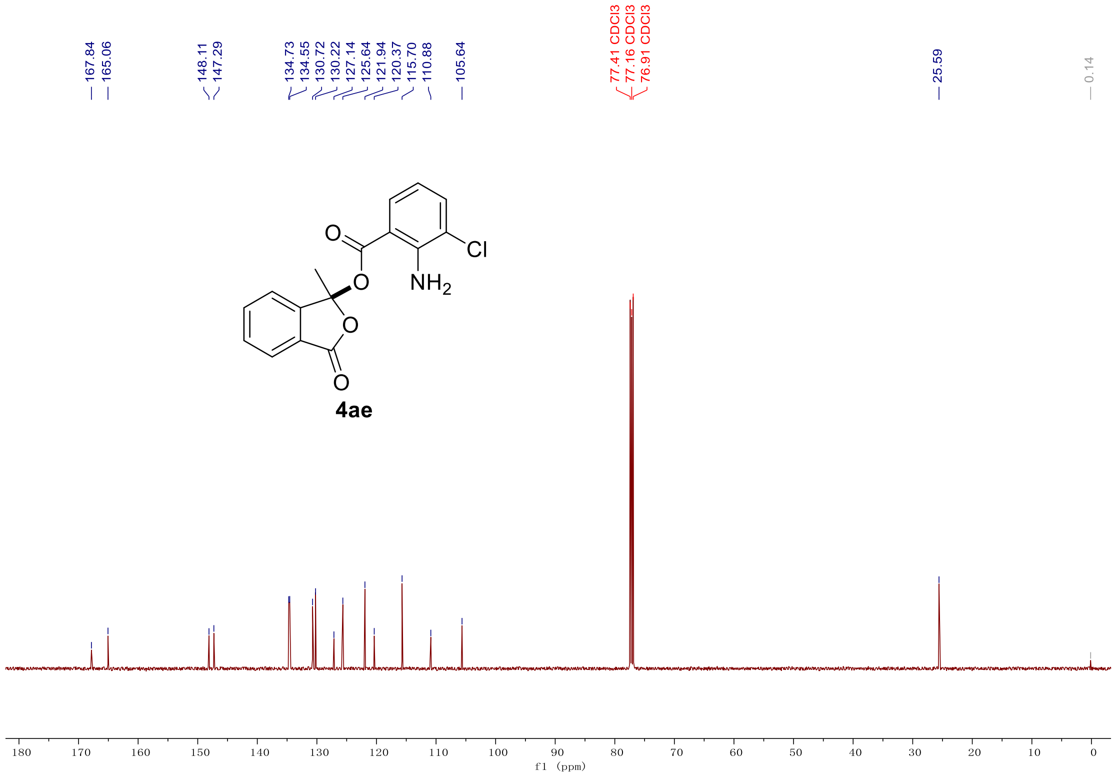

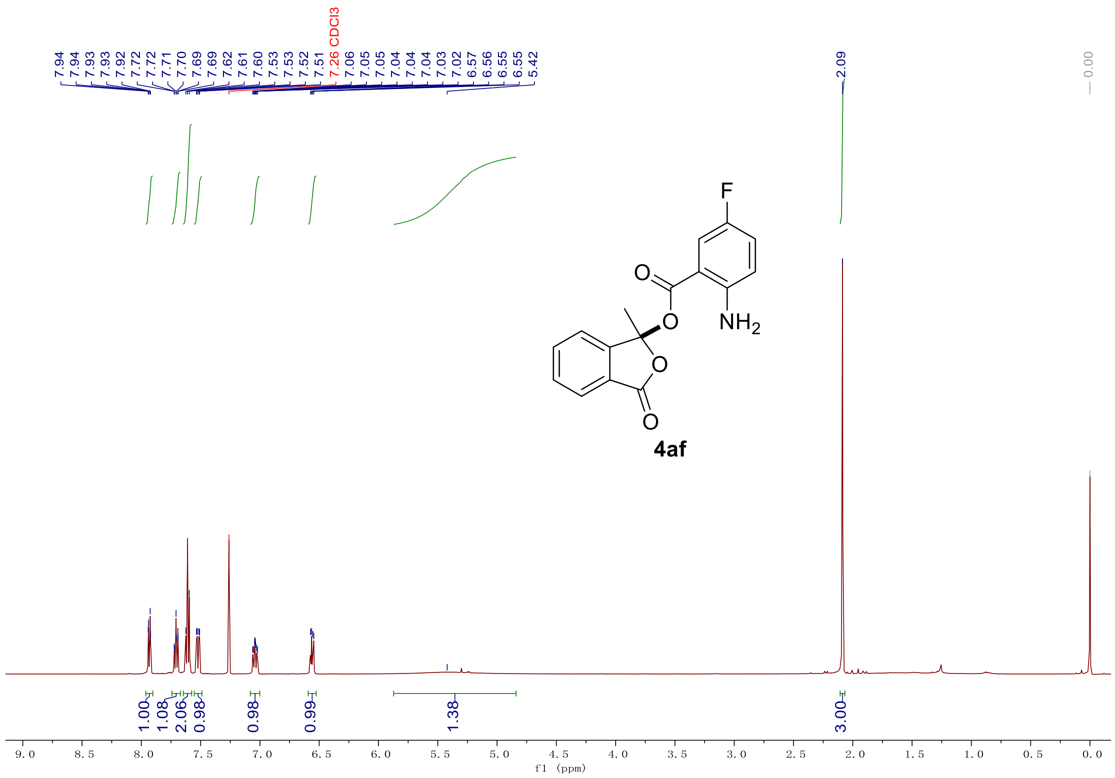
7.94
7.94
7.93
7.92
7.72
7.72
7.71
7.70
7.69
7.69
7.62
7.61
7.60
7.53
7.53
7.52
7.51
7.26 CDCl3
7.06
7.05
7.05
7.04
7.04
7.04
7.03
7.02
6.57
6.56
6.55
6.55
5.42
2.09
0.00
F
O
O
NH2
O
O
4af
1.00
1.08
2.06
0.98
0.98
0.99
1.38
3.00
f1 (ppm)

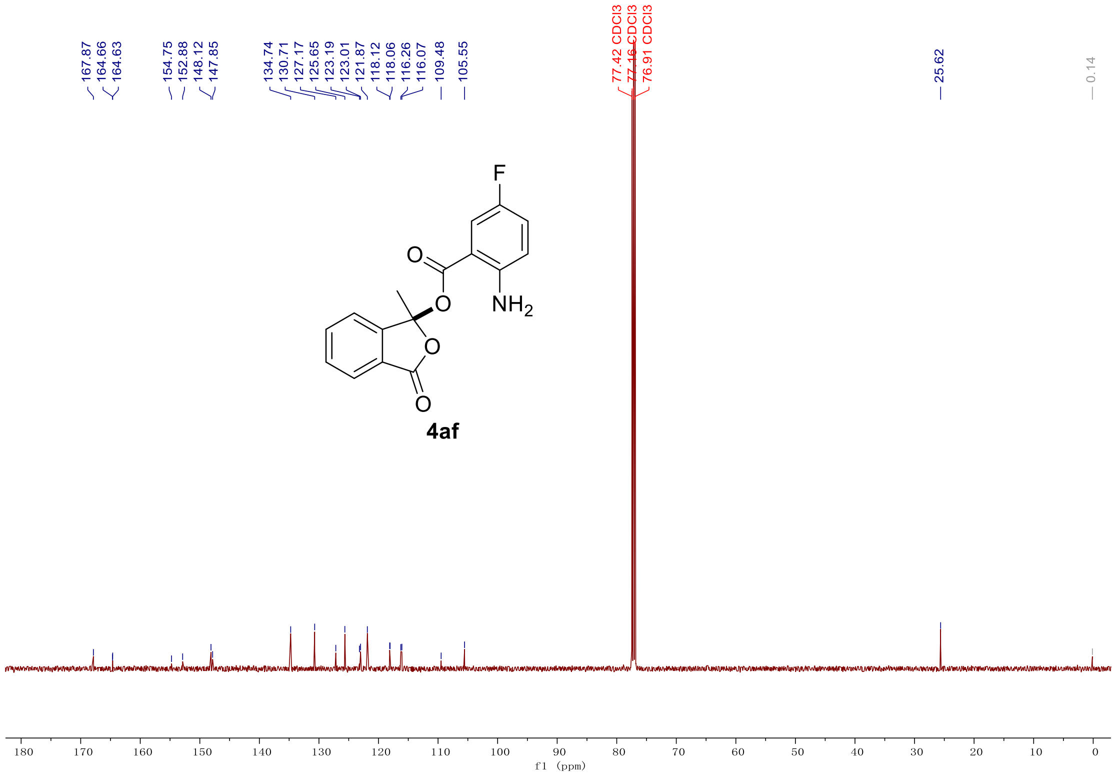
167.87
164.66
164.63
154.75
152.88
148.12
147.85
134.74
130.71
127.17
125.65
123.19
123.01
121.87
118.12
118.06
116.26
116.07
109.48
105.55
77.42 CDCl3
77.16 CDCl3
76.91 CDCl3
25.62
0.14
F
O
O
NH2
O
O
4af
f1 (ppm)

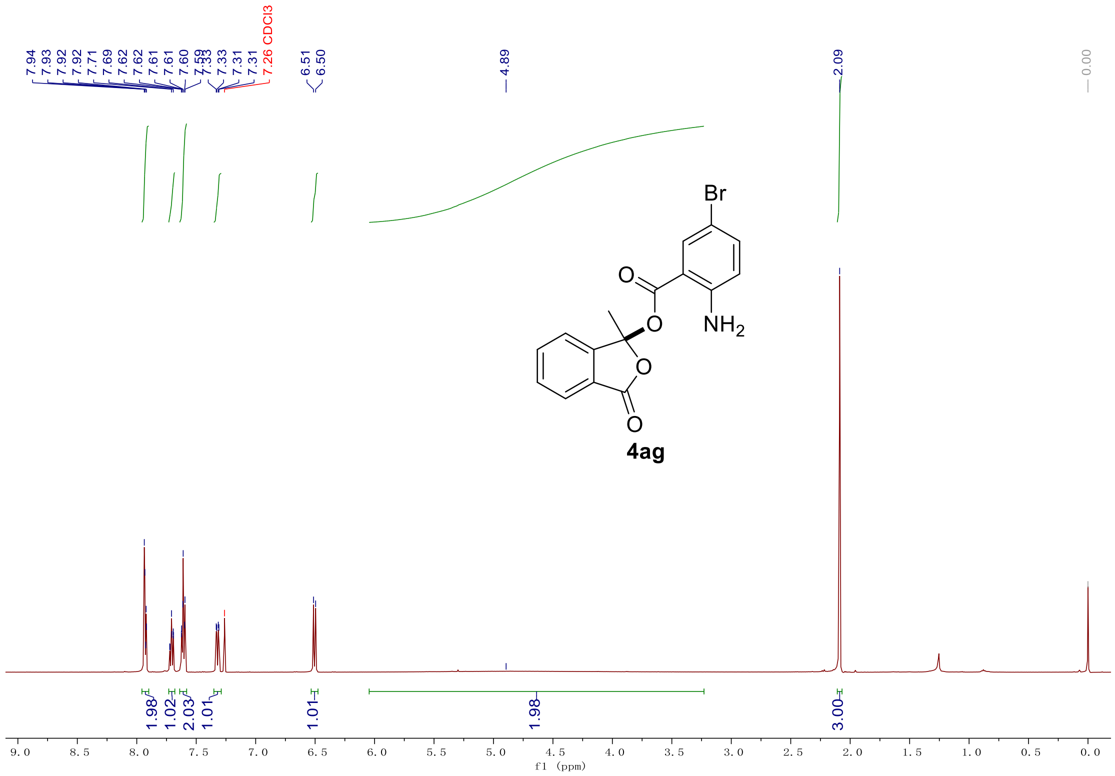
7.94
7.93
7.92
7.92
7.71
7.69
7.62
7.62
7.61
7.61
7.60
7.59
7.33
7.33
7.31
7.31
7.26 CDCl3
6.51
6.50
4.89
2.09
0.00
Br
O
O
NH2
O
O
4ag
1.98
1.02
2.03
1.01
1.01
1.98
3.00
f1 (ppm)

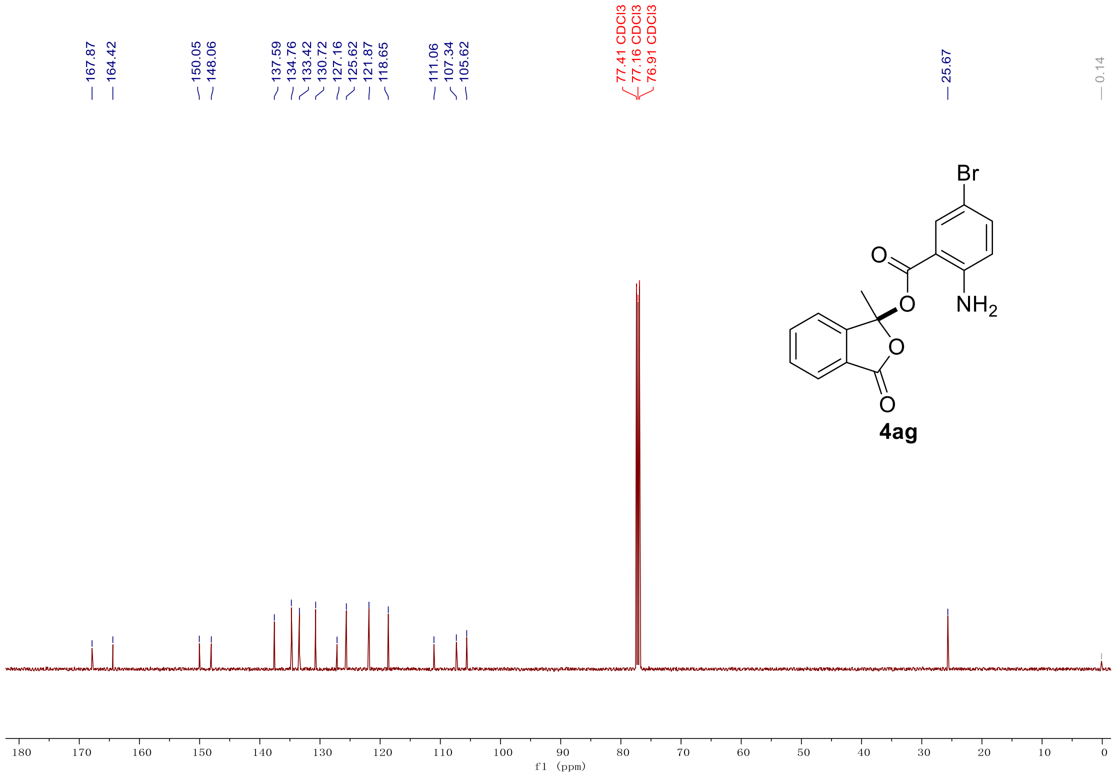
167.87
164.42
150.05
148.06
137.59
134.76
133.42
130.72
127.16
125.62
121.87
118.65
111.06
107.34
105.62
77.41 CDCl3
77.16 CDCl3
76.91 CDCl3
25.67
0.14
Br
O
O
NH2
O
O
4ag
f1 (ppm)

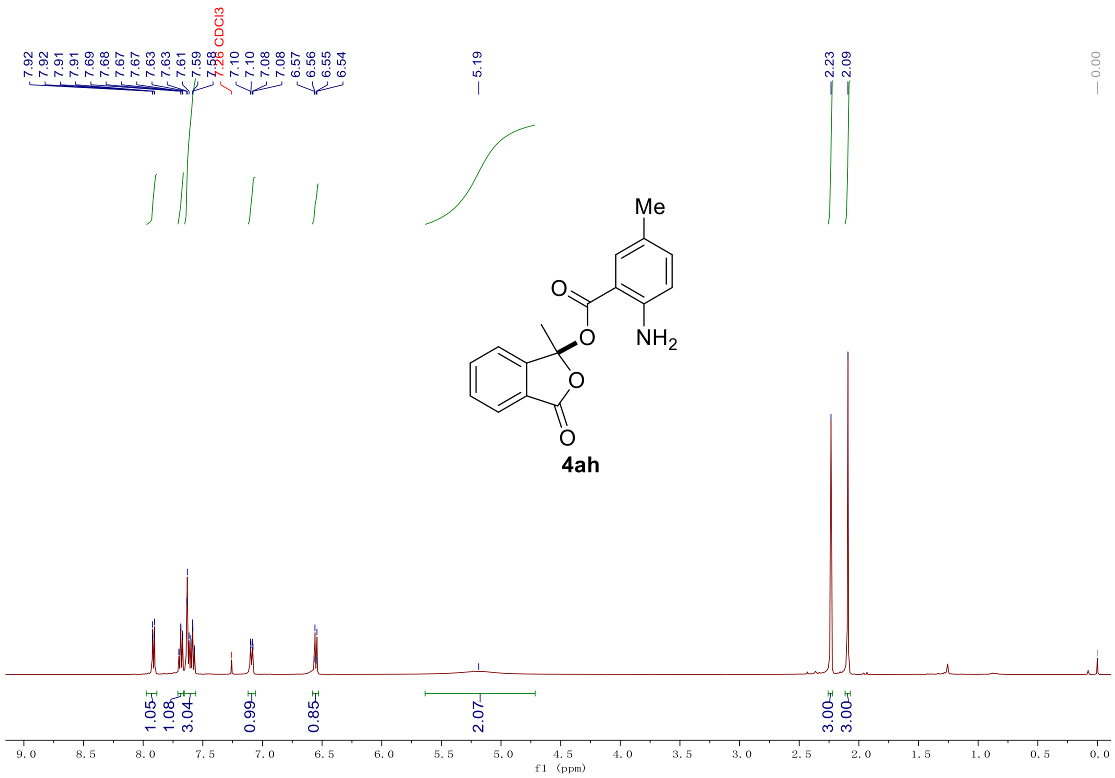

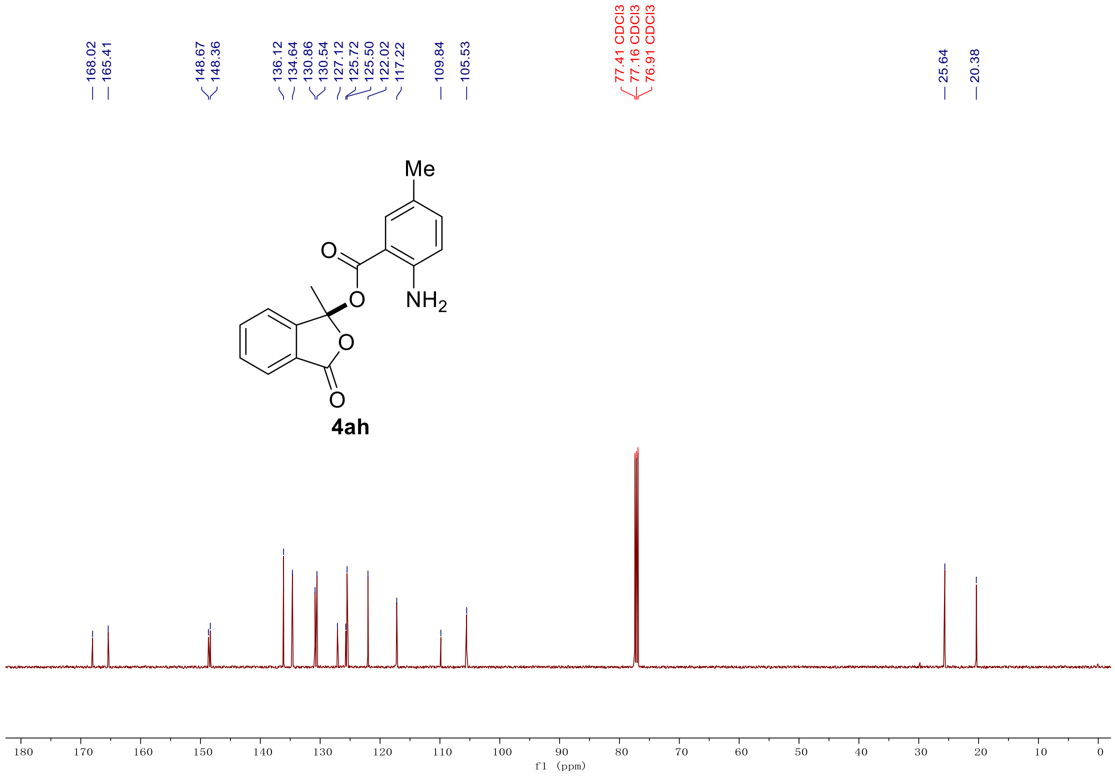

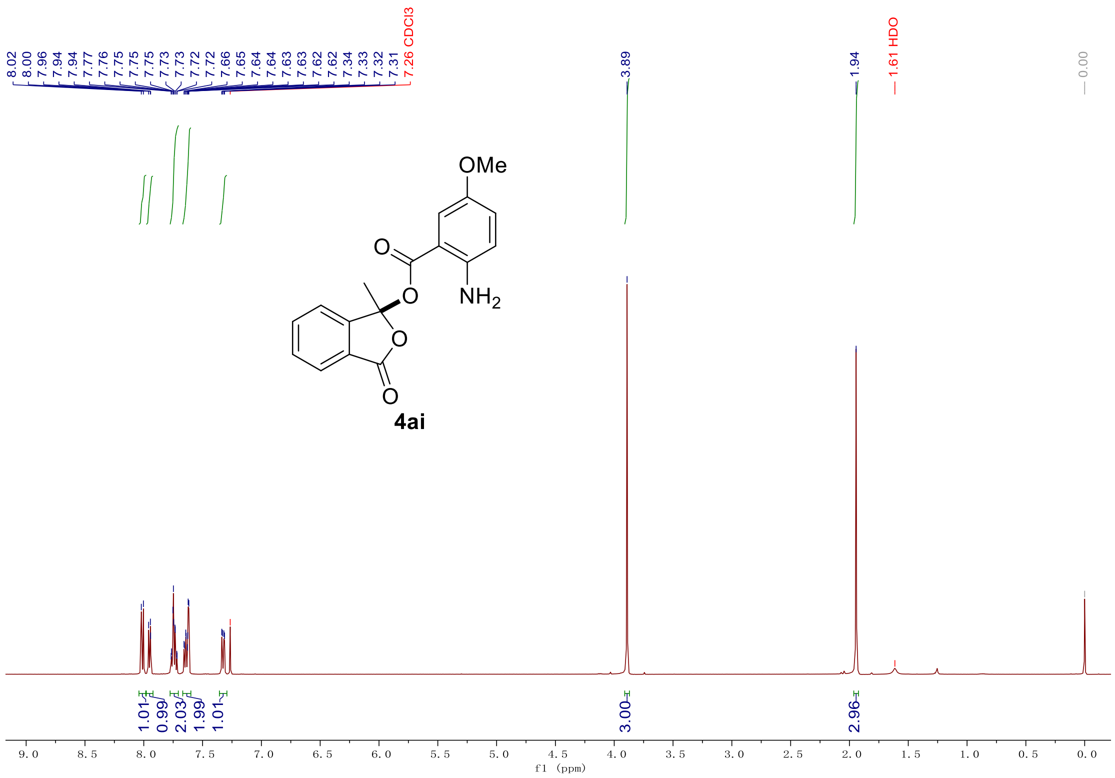

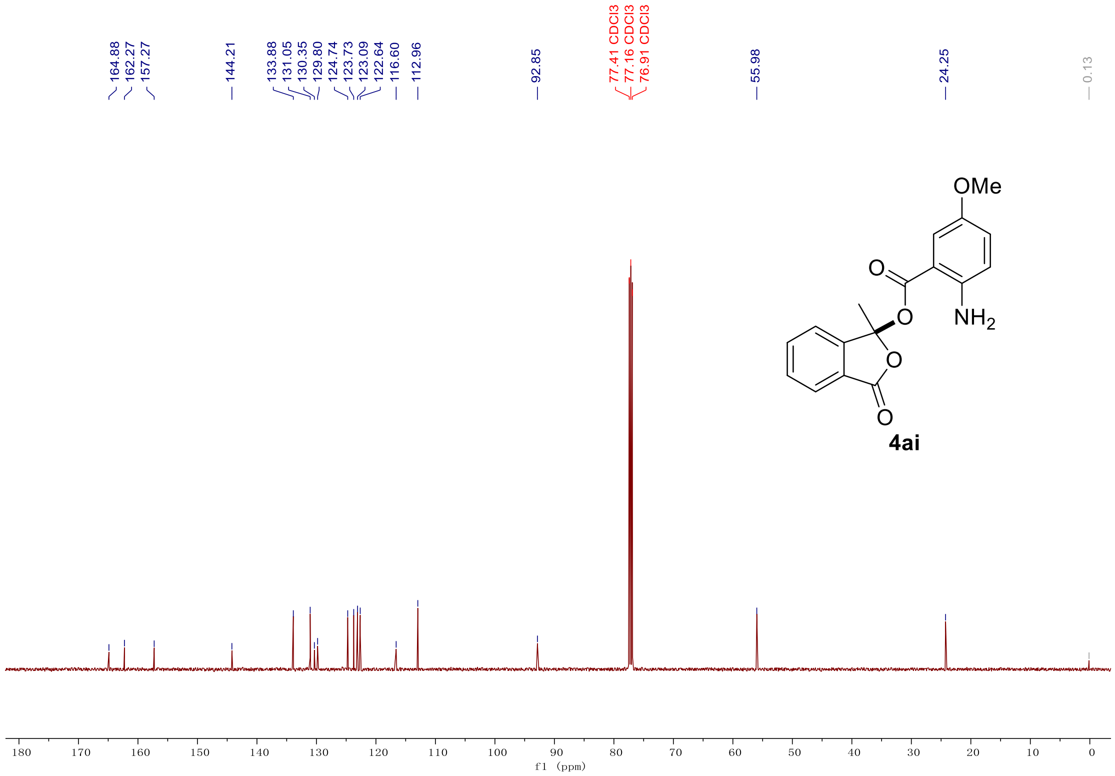

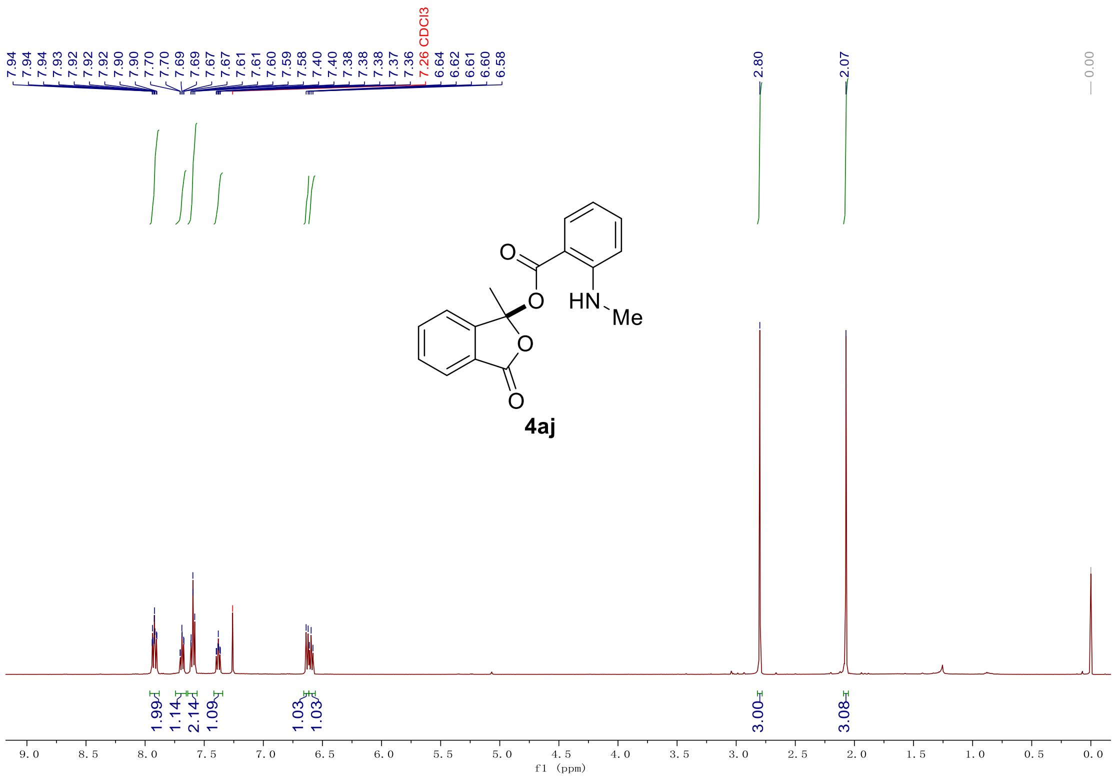

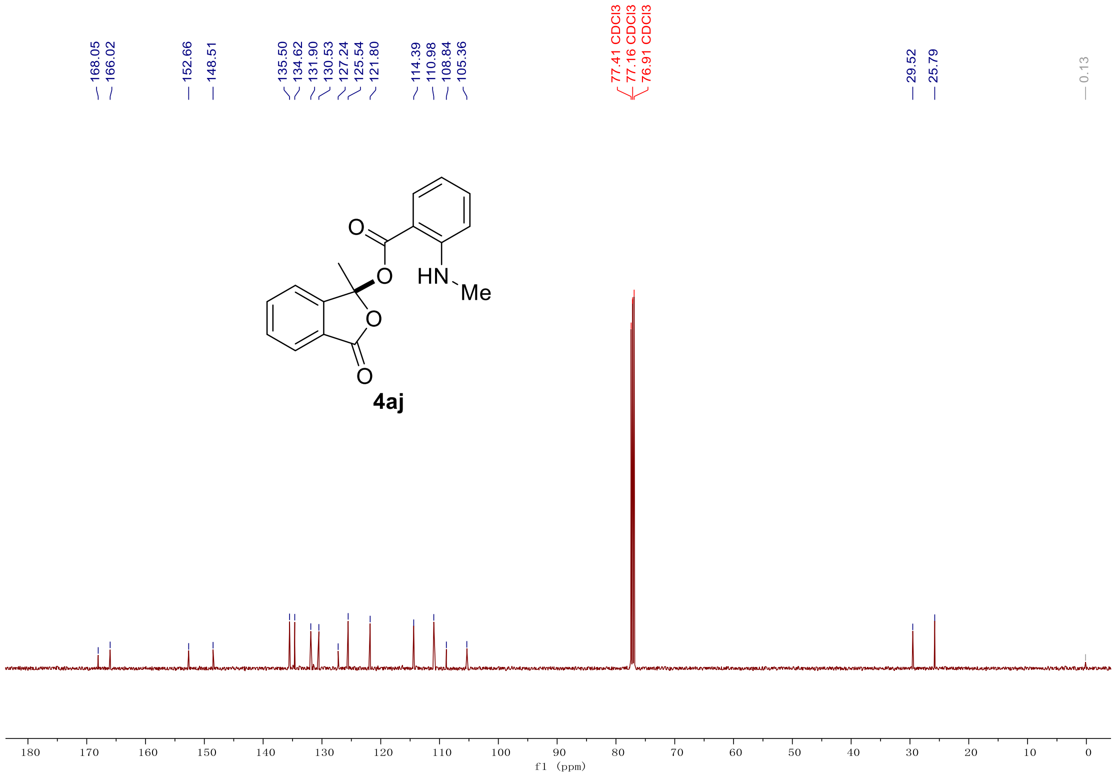

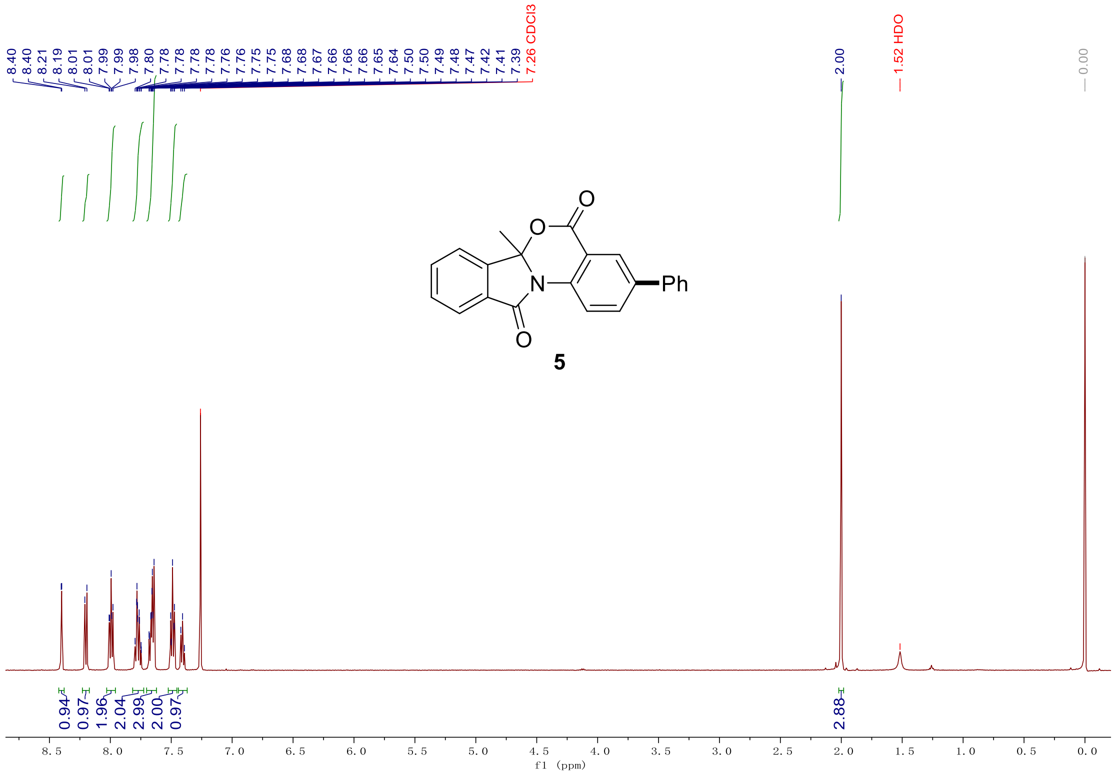
8.40 8.40 8.21 8.19 8.01 8.01 7.99 7.99 7.98 7.80 7.78 7.78 7.78 7.78 7.76 7.76 7.75 7.75 7.68 7.68 7.67 7.66 7.66 7.66 7.65 7.64 7.50 7.50 7.49 7.48 7.47 7.42 7.41 7.39
7.26 CDCl3
2.00
1.52 HDO
0.00
O
O
N
O
Ph
5
0.94 0.97 1.96 2.04 2.99 2.00 0.97
2.88
8.5 8.0 7.5 7.0 6.5 6.0 5.5 5.0 4.5 4.0 3.5 3.0 2.5 2.0 1.5 1.0 0.5 0.0
f1 (ppm)

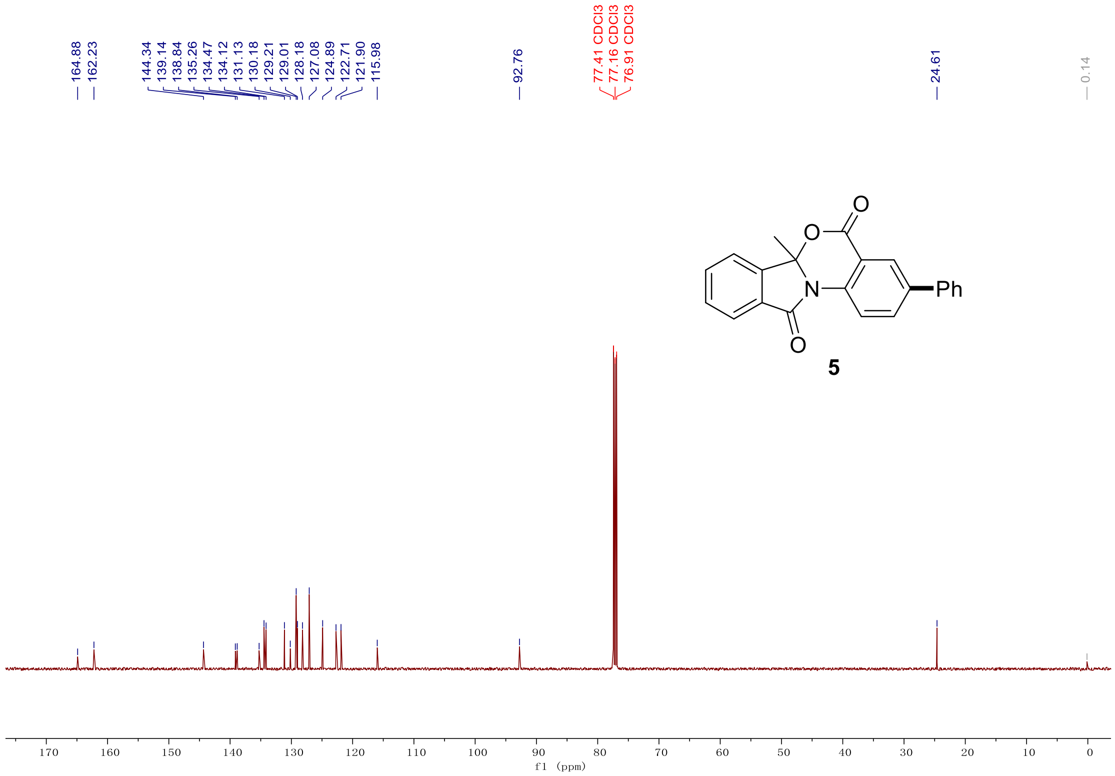
164.88 162.23
144.34 139.14 138.84 135.26 134.47 134.12 131.13 130.18 129.21 129.01 128.18 127.08 124.89 122.71 121.90 115.98
92.76
77.41 CDCl3
77.16 CDCl3
76.91 CDCl3
24.61
0.14
O
O
N
O
Ph
5
170 160 150 140 130 120 110 100 90 80 70 60 50 40 30 20 10 0
f1 (ppm)

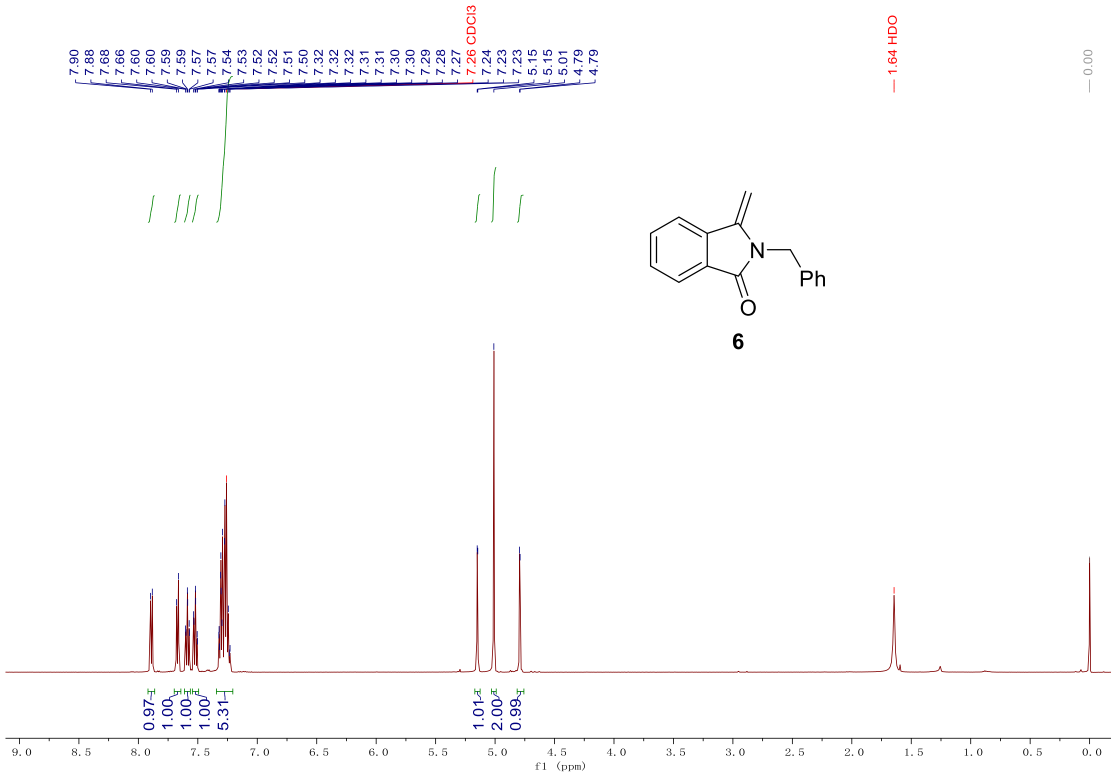
7.90 7.88 7.68 7.66 7.60 7.60 7.59 7.59 7.57 7.57 7.54 7.53 7.52 7.52 7.51 7.50 7.32 7.32 7.32 7.31 7.31 7.30 7.30 7.29 7.28 7.27 7.26 CDCl3 7.24 7.23 7.23 5.15 5.15 5.01 4.79 4.79
1.64 HDO
0.00
N
Ph
O
6
0.97 1.00 1.00 1.00 5.31 1.01 2.00 0.99
f1 (ppm)

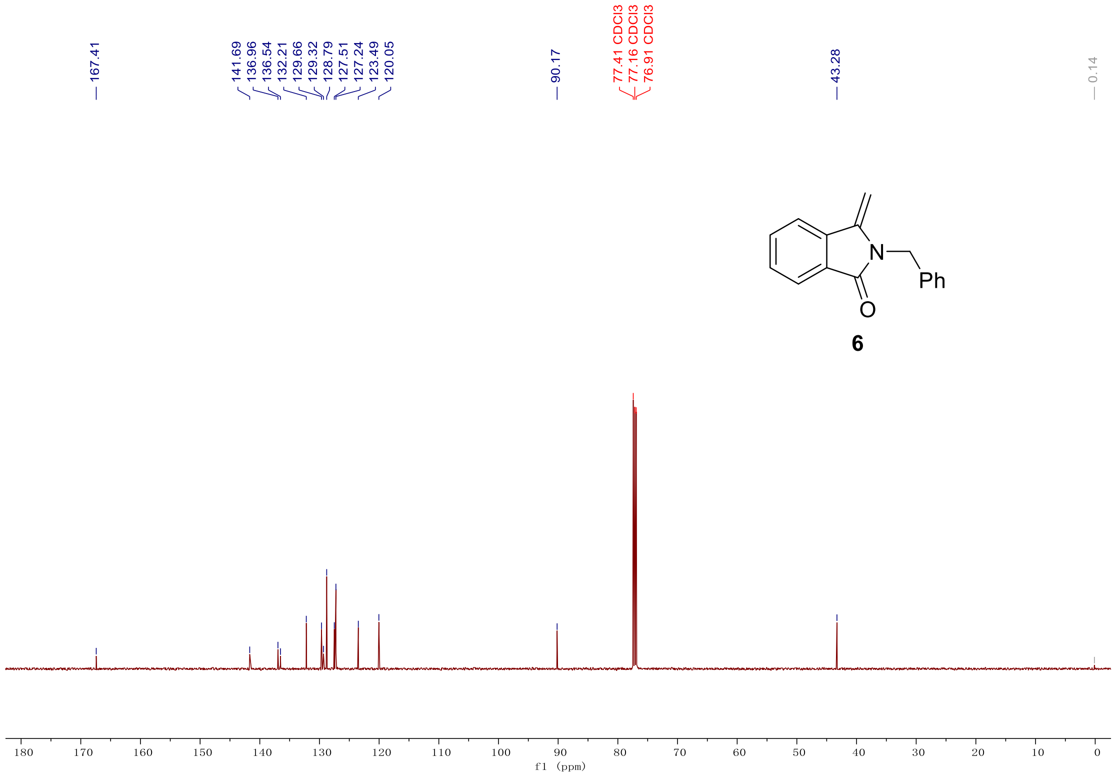
167.41
141.69 136.96 136.54 132.21 129.66 129.32 128.79 127.51 127.24 123.49 120.05
90.17
77.41 CDCl3 77.16 CDCl3 76.91 CDCl3
43.28
0.14
N
Ph
O
6
f1 (ppm)
